# Different drivers, same tick: Effect of host traits, habitat, and climate on the infestation of three rodent specie*s* by larval *Dermacentor* ticks

**DOI:** 10.1016/j.ijppaw.2025.101054

**Published:** 2025-03-07

**Authors:** Gabriel P. Andrade-Ponce, Brandi G. Giles, Brent C. Newman, Andrés M. López-Pérez, Cord B. Eversole

**Affiliations:** a Arthur Temple College of Forestry and Agriculture, Stephen F. Austin State University, Nacogdoches, TX, 75962, USA; b Department of Biology and Chemistry, Texas A&M International University, 5201 University Boulevard, Laredo, TX, 78041, USA; c Department of Agricultural and Environmental Sciences, Tennessee State University, Nashville, TN, 37209, USA; d Red de Biología y Conservación de Vertebrados, Instituto de Ecología A.C, Xalapa, Veracruz, 91073, Mexico

**Keywords:** Arid land ecology, Hard ticks, Host-parasite ecology, Rodents, Small mammals, Texas, Tick-borne diseases, Tick load, Tick presence

## Abstract

Tick-borne diseases (TBDs) pose a growing concern for public and wildlife health. Understanding how host traits and environmental factors influence tick infestation in small mammals is critical for improving TBD management strategies. We investigated the presence and load of *Dermacentor* spp. Larvae on three rodent species: *Peromyscus leucopus, Sigmodon hispidus,* and *Onychomys leucogaster*, in the arid brushland ecosystem of South Texas. We used generalized linear models to quantify how host, habitat structure, and climatic variables impact tick presence and load. Our results show that different drivers influenced tick infestation across species; *O. leucogaster* experienced higher tick loads in smaller individuals and habitats with more leaf litter, whereas for *P. leucopus,* infestation was determined by the reproductive state and sex of the host as well as larval activity throughout the year. None of the variables measured in this study adequately explained the presence and parasite load in *S. hispidus*. These findings highlight the importance of considering species-specific interactions between host traits and environmental factors for understanding the dynamics of ticks infestation in rodents. Our results contribute to a growing body of evidence on the complexity of tick-rodent host dynamics and offer insights for predicting changes in parasitism patterns and managing wildlife health in response to a changing environment in South Texas.

## Highlights

• Species-specific drivers influence *Dermacentor* tick infestation in two rodent species.
• Tick load was more sensitive to detect complex interactions than prevalence analyses.
• Reproductive activity and mild temperatures increase tick load in *Peromyscus leucopus.*
• Leaf litter cover and body size drive tick load in *Onychomys leucogaster.*

## Graphical abstract

**Figure:**
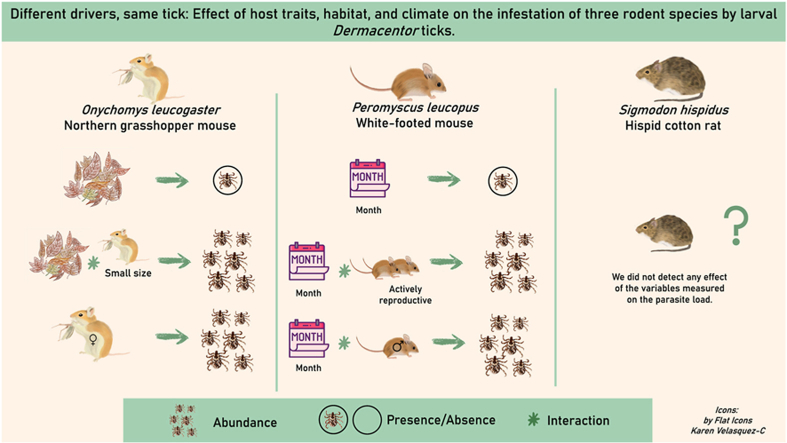

## 1 Introduction

Tick-borne diseases (TBDs) represent a significant emerging global public and veterinary health issue, particularly in the northern hemisphere where various TBDs are increasing in humans, including Lyme disease, rocky mountain spotted fever, and anaplasmosis, among others (Kumar et al., 2021; Pérez de León et al., 2014; Rodino et al., 2020; Swei et al., 2020). TBDs also have important implications for livestock, leading to economic losses (Betancur Hurtado and Giraldo-Rios, 2019; Hadush, 2016), and for wildlife, negatively affecting their survival (Morner and Beasley, 2012). Among tick species, hard ticks (Ixodidae family) encompass approximately 78% of the ⁓900 tick species described worldwide (Guglielmone et al., 2014), and are the most important vectors of TBDs in humans (Bouchard et al., 2019; Eisen et al., 2017). The life cycles of many hard tick species depend on small mammals, such as rodents, as hosts during their larval and nymphal stages (Eisen et al., 2017). Besides, rodents also play a key role in transmission dynamics since they are reservoirs of tick-borne pathogens (Barbour, 2017; Salkeld and Lane, 2010; Tsao et al., 2021), as well as inhabit both natural areas and close to human settlements (Han et al., 2015; Roy-Dufresne et al., 2013).

Understanding the factors that affect rodent susceptibility to tick infestation, is important to understand TBD ecology and is crucial for developing effective strategies to prevent and control TBDs (Randolph, 2008). Tick infestation of rodents is complex and is influenced by intrinsic and extrinsic factors. Intrinsic factors include host species identity, age, sex, and reproductive status. For example, reproductive males are often found to have higher infestation rates than females, likely due to differences in behavior, territorial activities, and hormonal profiles (Butler et al., 2020; Hughes and Randolph, 2001; Ostfeld et al., 1996). Larger body size also increases susceptibility to tick parasitism due to greater surface area for attachment and reduced competition among individual ticks and other ectoparasites (Butler et al., 2020; Poulin, 2004; Randolph, 2004). Extrinsic factors such as habitat structure and weather conditions, also affect rodent-tick interactions (Peralbo-Moreno et al., 2022; Shuai et al., 2022). Vegetation structure can create microhabitats conducive to tick life cycles and enhance concealment opportunities for rodents, potentially increasing host-tick encounters (Randolph, 2004; Randolph and Storey, 1999; Sonenshine, 2005). In addition, the increase in temperature and relative humidity during spring and summer is positively associated with nymphal and larval questing activity and survival (Brunner and Ostfeld, 2008; Gilbert, 2010; Randolph and Storey, 1999). The relative importance of intrinsic and extrinsic factors influencing tick infestation in rodents may vary depending on the life history and ecological characteristics of both ticks and the hosts, as well as the specific context or habitat (Fryxell et al., 2015; Kiffner et al., 2011; Peralbo-Moreno et al., 2022; Shuai et al., 2022). The variation in the relative importance of these factors makes predicting tick infestation a complicated task for ecologists and wildlife managers (Brunner and Ostfeld, 2008; Sumilo et al., 2007; Wilson et al., 2002). Therefore, more observational studies are needed to improve our understanding of tick and rodent infestation dynamics.

Within the United States, South Texas is an area with a relatively low incidence of tick-borne pathogens (Galán et al., 2022; Ginsberg et al., 2021). However, the natural movement of wildlife across state and national borders can influence the transmission and occurrence of pathogens (Feria-Arroyo et al., 2014; Giles et al., 2014; Gordillo-Pérez et al., 2009; Pound et al., 2010). Notably, pathogens such as *Rickettsia* spp and *Babesia* spp have been reported in wildlife within the state (Castellanos et al., 2016; Foley et al., 2017; Hodo et al., 2020; Modarelli et al., 2020; Olafson et al., 2020). Many tick species in the southern United States can remain active year-round under favorable conditions, with reduced activity during winter and summer months due to harsher environmental conditions (Chan and Kaufman, 2009; McEnroe, 1979). In addition, many rodent species in the region have the potential to serve as reservoirs for tick-borne pathogens, including *Peromyscus* species (Galán et al., 2022; Rodriguez et al., 2015), which can breed year-round when resources are sufficient (Lackey et al., 1985). All these characteristics make South Texas a region of constant risk for the spread of TBDs (Williamson et al., 2010). Recognized as an area of concern, South Texas has seen progress in monitoring both tick species and their hosts (Busch et al., 2014; Castellanos et al., 2016; Charles et al., 2012; Galán et al., 2022; Olafson et al., 2018; Rodriguez et al., 2015). However, to date, there are few studies that directly examine how environmental and host variables influence tick infestation in rodents in South Texas.

In this study, we quantified the importance of host and environmental factors in explaining tick presence and tick load and the in rodents inhabiting the arid South Texas scrub brush ecosystem. Specifically, we test how host sex, reproductive status, body mass of rodents, habitat structure, temperature, rainfall, and seasonality influence the *Dermacentor* spp. Larvae infestation of three rodent species including *Onychomys leucogaster* (Northern grasshopper mouse), *Peromyscus leucopus* (White-footed mice), and *Sigmodon hispidus* (Hispid cotton rat). Understanding the interplay between habitat and host factors as well as their broader climatic drivers is imperative to advance the scientific understanding of host-tick interactions, informing one health strategies and adapting management approaches accordingly.

## 2 Materials and methods

### 2.1 Study area

We conducted this study in Laredo, Texas, USA, near the Texas A&M International University campus (27.574132° N, 99.429042° W). This area has an elevation of 155 m and is characterized as the South Texas Plains vegetation region (Gould, 1962), which lies within the Tamaulipan Biotic Province (Judd et al., 2023). Climate is characterized by short mild winters and hot summers. The average temperature for this area is 23^o^ C, with winter months (December–February) ranging from an average low temperature of 9^o^ C to an average high temperature of 20^o^ C and summer months (June–August) ranging from an average low temperature of 25^o^ C to an average high of 37^o^ C (Climate United States, 2024). Rainfall patterns peak slightly in the spring and fall and the average precipitation per year is 52 cm (Beck et al., 2011).

### 2.2 Rodent sampling

**Fig. 1:**
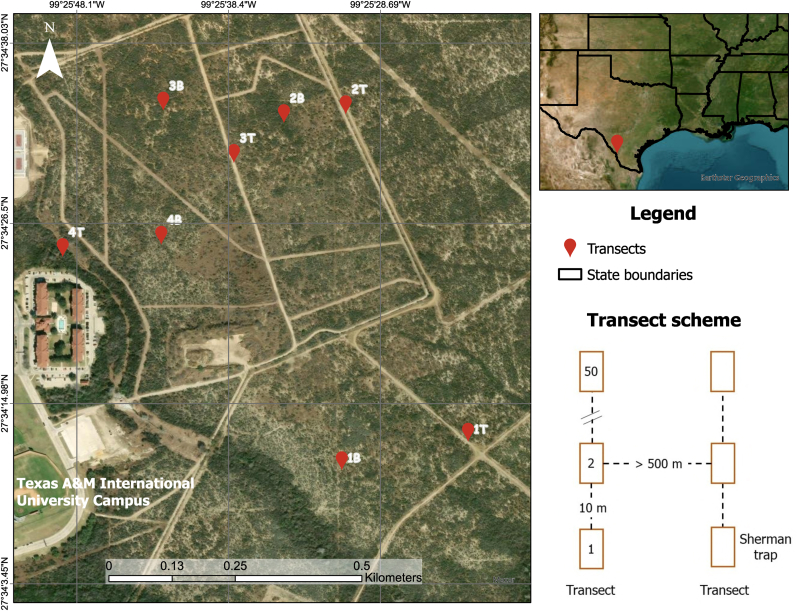
Map of the spatial distribution of Sherman trap transects for sampling rodents. Each transect consisted of 50 traps separated by 10 m.

We sampled rodents and their ectoparasites from September 2019 to November 2020, avoiding days with freezing temperatures (≤0 °C) and excluding the summer months (May–August) to minimize small mammal mortality due to extreme weather conditions (Sikes et al., 2011). Sampling was conducted using eight transects of 50 Sherman live traps spaced 10 m apart, for a total of 400 traps (Fig. 1). Trap transects were spaced ≥500 m apart to minimize spatial autocorrelation issues. We used transect sampling as it has proved to be more successful in previous studies (Pearson and Ruggiero, 2003). Each transect was sampled for 5 consecutive days per month, where two transects were randomly selected and rotated weekly to eliminate any time confounding biases. The total number of sampling nights was 124, with a total effort of 12,400 trap nights.

Traps were baited each evening at 18:00 h with a seed mixture and checked the following morning at 06:30 h. All captured individuals were identified to species, and data were recorded on sex, reproductive status (active vs. inactive, based on traits such as enlarged testes, signs of pregnancy, lactation, or a perforated vagina), tail length (mm), and ear length (mm). To identify recaptured individuals, we marked them using hair dye and toe clipping after data collection, then released them at the capture site. Based on previous evidence (Borremans et al., 2014; Haines et al., 2018), these marking techniques are not expected to affect the survival of captured rodents. Rodent sampling and handling were conducted under the Texas A&M International University IACUC permit (committee approval #2018–2) and the Texas Parks and Wildlife Department Scientific Permit for Research (SPR-0820-220).

The presence and number of tick ectoparasites observed on captured rodents were noted following a ∼120-s whole-body inspection (Hoffmann et al., 2010). Ticks were removed and preserved in 70% ethyl alcohol and stored for subsequent identification. Dichotomous keys were used to identify ticks to genus and life stage (Brinton et al., 1965; Coley, 2015; Cooley and Kohls, 1944; Furman and Loomis, 1984; Keirans and Durden, 1998).

### 2.3 Vegetation and habitat measurement

We utilized a modified point quarter-belt transect sampling technique to sample vegetation along each trapping transect during the spring and fall seasons (Mueller-Dombois and Ellenberg, 1974). We sampled vegetation at five points located every 100 m along each transect. From the center point, belt transects were extended to 10 m, in each cardinal direction. Along each belt transect at every 0.5 m we recorded percentages of litter cover, herbaceous material, and vegetation height. We measured litter depth (cm; via steel tape measure) every 2 m along each transect. Lastly, at five and 10 m away from the center point, we used a spherical densitometer to determine the percentage of canopy cover. We calculate the average values for each measurement for each trapping transect.

### 2.4 Weather information

We measured temperature (^o^C) and rainfall (mm) throughout our study using a single HOBO U30 weather station (Onset Computer Corporation, Bourne, Massachusetts, USA) that was located at our study site. Temperature and rainfall were measured hourly and individual measurements were used to calculate average daily values.

### 2.5 Data analysis

We used generalized linear models (GLMs) to identify which host, habitat, and climate variables affect tick presence or load. Due to the sample sizes of parasitized individuals and the number of ticks per individual, we restricted the analyses to *P. leucopus*, *O. leucogaster,* and *S. hispidus* host species, and *Dermacentor* sp. Tick larvae. We analyzed each rodent species separately. For the analysis of tick presence, we used GLMs with a Bernoulli distribution, as the response variable was the presence or absence of ticks (Forbes et al., 2011). For tick load (counts) analysis, we selected the appropriate error distribution from Poisson, Negative Binomial, and Tweedie families using the Akaike Information Criteria corrected for small sample (AICc) and overdispersion test (Zuur et al., 2009). The model distribution with the lowest AICc value and no signs of overdispersion (i.e., the ratio of residual deviance to residual degrees of freedom ∼1) was chosen for each case.

To select the model structure with the most empirical support for the analyses of tick presence and tick load, we followed the protocol by Zuur et al. (2009). First, we identified possible variation and dependence of space by fitting models with transect as random effects (Table 1). Models with different random intercept effects and no random effects were ranked using AICc. We tested the adequacy of the selected model by inspecting simulated residuals (Hartig and Lohse, 2022) and assessing any potential spatial correlation using Moran's I index (Fox et al., 2015).

**Table 1:** Description of the variables used to model tick presence and load on small mammal hosts in South Texas, USA.

Variable (Code)	Description	Variable type	Hypothesis	Expected relation
Tick presence	Detection or non-detection of ticks in each rodent individual.	Response variable	NA	NA
Tick load	The number of tick counts for each rodent individual captured	Response variable	NA	NA
Body weight (g) (Wght)	The weight of each rodent individual captured (g)	Host explanatory variable - Continuum	Hosts with greater body size represent more habitat and resources for ticks (Kuris et al., 1980; Poulin, 2004). Therefore, bigger individuals will favor greater presence and number of ticks.	Linear/Quadratic
Sex (Sex)	The sex of each rodent captured	Host explanatory variable - Categorical: Male (M), Female (F)	Male mice tend to move more in the landscape compared to females. Therefore, males are expected to have higher tick burdens than females (Butler et al., 2020; Ostfeld et al., 1996).	NA
Reproductive status (Rep)	The reproductive status of each captured rodent. Reproductive and non-reproductive	Host explanatory variable - Categorical: non-reproductive (N), reproductive (Y)	The hormonal profile and the higher activity of sexually active mice make them more susceptible to a higher parasite load. Therefore, we expect that sexually active mice will have a higher chance of having a greater tick load (Ostfeld et al., 1996; Schmidt et al., 1999).	NA
Average litter cover (Avg_LC)	Percent (%) presence of litter cover in each transect	Habitat explanatory variable - Continuum	Higher numbers of questing tick larvae are associated with areas with higher leaf litter cover, as these locations allow them to avoid adverse environmental conditions (Sonenshine, 2005). We expect that individual mice captured in areas of higher cover and depth of leaf litter will have higher tick larvae burdens.	Linear
Average litter depth (Avg_LD)	Average of litter depth in each transect (cm)	Habitat explanatory variable - Continuum		Linear
Average Herbaceous material (Avg_HC)	Percent (%) presence of Herbaceous in each transect	Habitat explanatory variable - Continuum	Herbaceous cover favors microclimatic conditions for tick larval survival and therefore tick abundance (Sonenshine and Stout, 1968). We expect that mice captured in areas with higher grass cover will have higher tick burdens.	Linear
Average Canopy Cover (Avg_CC)	Percent (%) of canopy coverture in each transect	Habitat explanatory variable - Continuum	Greater canopy cover increases humidity and reduces direct sunlight on the ground, which favors climatic conditions for tick larval development (Ginsberg et al., 2020; Leal et al., 2018; Zamora et al., 2020). We expect that individual mice captured in areas with higher canopy cover will have higher tick burdens.	Linear
Average vegetation height (Avg_VH)	Average vegetation height for each transects (cm)	Habitat explanatory variable - Continuum	Tick larvae prefer vegetation with a height of less than 50 cm because it favors climatic conditions for their development (Prusinski et al., 2006). For this reason, we expect that captured mice will have higher tick loads in areas with lower vegetation height.	Linear/Quadratic
Rain (Rain)	Rainfall measure (mm) for each day of the survey with Hobo data loggers	Weather explanatory variable - Continuum	Rainfall can negatively affect the development of tick larvae by washing and flooding microhabitats. In addition, a correlation has been reported between drier areas and periods of the year and tick larval abundance (Randolph and Storey, 1999). Therefore, we expect that rodent captured during periods of higher rainfall will have a lower parasite load.	Linear
Temperature average (Temp)	Temperature (C) measure for each day of the survey with Hobo data loggers	Weather explanatory variable - Continuum	Ticks show a peak of activity during warm weather periods and areas (Gilbert, 2010). However, extreme temperature or drought conditions may affect larval survival by desiccation (Teel et al., 2010). Therefore, we expect that mice captured during periods of very low or very high temperatures will have lower tick burdens.	Linea/Quadratic
Season (Season)	The season in which the survey was conducted	Weather explanatory variable - Categorical: Spring (March 1st – May 31st), Fall (September 1st- November 30th), and Winter (December 1st – February 28th)	Ticks and their larvae exhibit seasonal peaks of activity (Brunner and Ostfeld, 2008). In the southern United States, tick larval activity patterns are greatest during the spring because weather conditions are more favorable than summer and winter (Chan and Kaufman, 2009; McEnroe, 1979). Therefore, we expect that rodents captured during the spring survey will have a higher tick load.	NA
Month (Month)	The month of the year in which the individuals were captured	Time control explanatory variable - Categorical: Month of the year represented in integer form 1: January to 12: December	Variable used as a control for the time effect. The month factor allows to identification of influences of phenological, environmental, or climatic dynamics that are not captured by the other variables used.	NA
Transect (Transect)	ID of the transect in which each individual was captured	Random effects variable	NA	NA

Host, habitat, and climatic variables related to the ecology of tick infestation in rodents were used as fixed effects variables (Table 1). The host variables included sex, reproductive status, and body weight of each captured mouse. Habitat variables comprised average litter cover and depth, herbaceous cover, canopy cover, and vegetation height. Climatic variables encompassed daily rainfall, temperature, and climatic season. Additionally, we examined the effects of the square form of body weight, vegetation height, and temperature on tick presence and tick load (Table 1). To reduce the number of variables and the complexity of the models, we used a two-step approach to select the best fixed-effect model structure. First, we independently identified the most important models of host, habitat, and weather variables separately (Table 1). For each group of variables, we created candidate models with only one predictor and used AICc to rank them. Second, we combined all previously selected variables to rank models with single predictors and second-order additive and multiplicative interactions using AICc. In the final model selection, we also included models with the month alone as a fixed factor and models with selected variables and their interaction with month. These models allow us to evaluate the changes in the effects of the variables over time (i.e., month). Models with a Δ AICc <2 were considered equally supported and selected at each step (Burnham and Anderson, 2002). If the model with only the intercept (no predictor) was included among the best models during the final selection, we concluded that none of the variables could explain tick presence or tick load. Models with uninformative variables (i.e., 85% CI of the regressor coefficient overlaps 0), were excluded from the final inference (Arnold, 2010; Sutherland et al., 2023). Finally, we inspected the adequacy of the selected models using a simulated residual approach (Hartig and Lohse, 2022).

All analyses were conducted using the R programming language version 4.3.3 (R Core team, 2024) (Appendix A). Generalized linear (mixed) models (GLM(M)s) were fit using a frequentist approach with the glmTMB package (Brooks et al., 2017). AICc calculations were performed using the performance (Lüdecke et al., 2021) and AICcmodavg (Mazerolle, 2020) packages. The Moran's I index and splines were calculated using the ncf package (Bjornstad and Cai, 2022) with 5000 resampling iterations. The DHARMa package (Hartig and Lohse, 2022) was used for the estimation and visual inspection of the simulated residuals as well as conducting the dispersion test.

## 3 Results

**Fig. 2:**
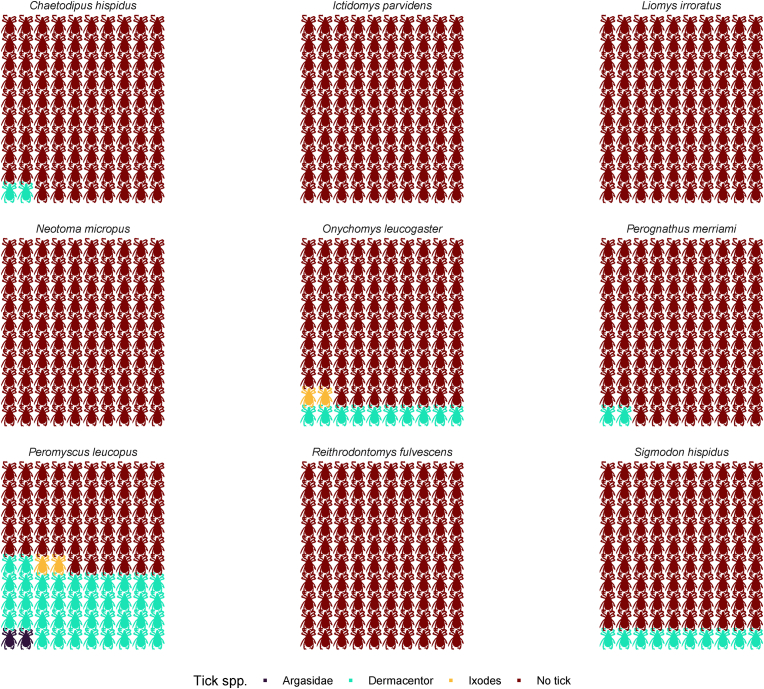
The proportion of rodent individuals infected by different larval tick genera. Note that ticks of the genus *Amblyomma* are not included because only nymphs and adults were recorded.

We captured a total of 1076 unique individual rodents representing nine species: *Peromyscus leucopus*, *Sigmodon hispidus*, *Onychomys leucogaster*, *Chaetodipus hispidus*, *Perognathus merriami*, *Liomys irroratus*, *Reithrodontomys fulvescens*, *Neotoma micropus*, and *Ictidomys parvidens* (Table 2). Across all species, 452 recaptures were recorded. To avoid pseudoreplication, recaptured individuals were not included in the analysis. We collected 717 ticks from rodents belonging to the genera *Dermacentor*, *Ixodes*, *Amblyomma*, and the family Argasidae (Table 2). Across all captured rodents, the mean tick infestation prevalence was 18.7%. The *Dermacentor* sps. Larvae were the most common tick species and were present in 64% of the infested rodents (Fig. 2).

**Table 2:** Number of individual rodents collected and the presence of different tick species, life stages, and abundance (in parentheses), collected from rodents’ species in southern Texas, USA.

Rodent species	Dermacentor		Ixodes		Argasidae	Amblyomma		No tick	Totals
	Larvae	Nymph	Larvae	Nymph	Larvae	Nymph	Adult		
*Chaetodipus hispidus*	1 (1)	0	0	0	1 (1)	0	0	131	133 (1)
*Ictidomys parvidens*	0	0	0	0	0	0	0	4	4 (0)
*Liomys irroratus*	0	0	0	0	0	0	0	22	22 (0)
*Neotoma micropus*	0	0	0	2 (7)	0	0	0	6	8 (7)
*Onychomys leucogaster*	9 (20)	1 (1)	0	0	0	0	0	115	125 (22)
*Peromyscus leucopus*	101 (474)	31 (68)	8 (20)	0	1 (2)	0	0	230	136 (548)
*Perognathus merriami*	1 (2)	0	0	0	0	0	0	141	142 (2)
*Reithrodontomys fulvescens*	1 (1)	0	0	0	0	0	0	15	16 (1)
*Sigmodon hispidus*	21 (68)	24 (45)	0	0	0	4 (10)	7 (12)	485	541 (135)
Total	134 (566)	56 (114)	8 (20)	2 (7)	2 (3)	4 (10)	7 (12)	1149	1362 (732)

**Fig. 3:**
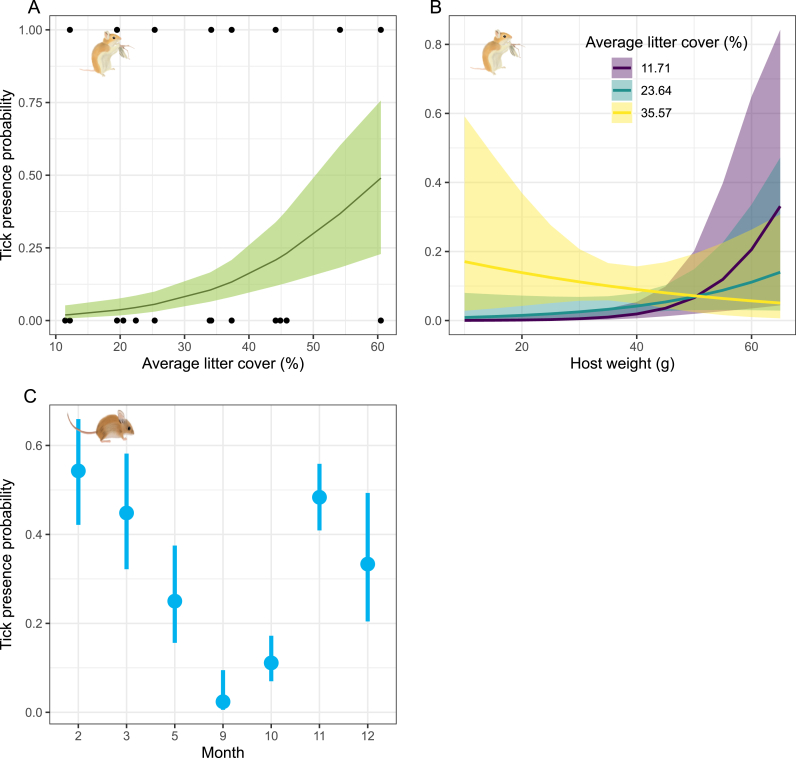
Marginal prediction and 85% confidence intervals of *Dermacentor* larvae presence based on selected models for each rodent species. A.-B. Results for *O. leucogaster*; C. for *P. leucopus*. Rodent illustrations made by Karen Velasquez C.

For the final selection of tick presence models, we ranked 32 models for *O. leucogaster (*Supplementary File 1; Supplementary Table S1), 63 for *P. leucopus (*Supplementary File 1; Supplementary Table S2), and 12 for *S. hispidus (*Supplementary File 1; Supplementary Table S3). Based on AICc, the absence of uninformative variables, and goodness-of-fit, we selected two models for *O. leucogaster*: one including only the mean litter cover and another incorporating the interaction between litter cover and host weight *(*Supplementary Table S4). For *O. leucogaster*, selected models showed that lower-weight individuals and individuals in areas with a higher percentage of litter cover were more likely to carry Dermacentor larvae (Fig. 3 A; B; Supplementary Table S4). For *P. leucopus*, a single model with only the month fixed factor was selected (Supplementary Table S4). Model results indicated that *P. leucopus* is most likely to carry *Dermacentor* larvae in February, March, November, and December, while September and October have the lowest predicted probability of tick presence (Fig. 3 C; Supplementary File 4). In the case of *S. hispidus* among the models selected by AICc, only two had coefficients with 85% confidence intervals that did not overlap zero. These were the model that included reproductive status with the quadratic term of vegetation height, and the model incorporating the interaction between the quadratic term of vegetation height and climatic season. However, both models exhibited signs of heteroscedasticity and were therefore not considered for the inference (Supplementary File 1).

**Fig. 4:**
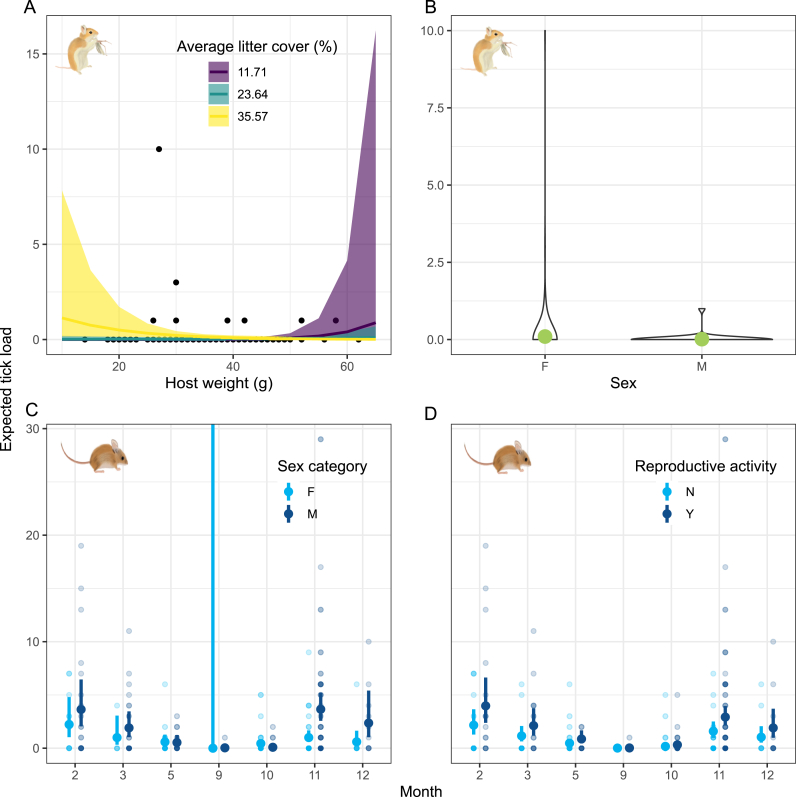
Marginal prediction and 85% confidence intervals of *Dermacentor* larvae load based on selected models for each rodent species. A.-B. Results for *O. leucogaster*; C-D. results for *P. leucopus*. Rodent illustrations made by Karen Velasquez C.

In the final selection of parasite load models, we ranked 28 models for *O. leucogaster (*Supplementary File 2; Supplementary Table S5), 34 for *P. leucopus (*Supplementary File 2; Supplementary Table S6), and 25 for *S. hispidus (*Supplementary File 2; Supplementary Table S7). After filtering models based on confidence interval inspection and goodness-of-fit assessment, our inference on *O. leucogaster* parasite load was based on three models: one including the interaction between host weight and mean litter cover, another incorporating sex and litter cover, and a third considering only litter cover *(*Supplementary Table S8). Similar to the presence analyses, we found that areas with higher percentages of litter cover increased the expected tick load in *O. leucogaster*, especially for individuals with lower body weights (Fig. 4A). Additionally, the model showed that females exhibited a higher parasite load than males (Fig. 4B; Supplementary Table S8). For *P. leucopus*, our inference was based on two models: one where parasite load was explained by the interaction between month and sex, and another that included month and reproductive status (Supplementary Table S8). The models indicated that *Dermacentor* tick larvae load was generally higher in males, particularly in November, December, February, and March (Fig. 4C). Regarding the reproductive status of *P. leucopus*, parasite load was consistently higher in actively reproducing individuals across all months, with this trend being especially pronounced in November, December, February, and March (Fig. 4D). For *S. hispidus*, three models were selected. The first included the month and the quadratic term of vegetation height; however, we could not estimate confidence intervals for this model, preventing us from drawing inferences *(*Supplementary File 2). The second model included only the month fixed factor, while the third incorporated an interaction between month and reproductive status. The latter two models exhibited heteroscedasticity and wide confidence intervals, respectively, making inferences from them unreliable *(*Supplementary File 2).

## 4 Discussion

Understanding how environmental and ecological factors influence tick infestation in rodents is essential for improving the control and prevention of tick-borne diseases (TBDs). In this study, we identify how host traits, habitat, and climate variables affect the presence and load of *Dermacentor* tick larvae on rodent species in southern Texas, USA. Our results indicate that the factors driving tick presence and tick load differ between the three rodent species. In *O. leucogaster*, higher litter cover and lower host weight were associated with increased tick burden, whereas tick load in *P. leucopus* differed among months, and depended on the reproductive status and sex. In contrast, none of the measured variables adequately explained tick infestation in *S. hispidus*. Moreover, we found that tick load in rodents was influenced by interactions between variables, underscoring the complexity of tick-host relationships.

Among the rodent species sampled in this study, *P. leucopus* had the highest *Dermacentor* tick load per individual. Species of *Peromyscus* are common across North America and important hosts for the immature stages of many hard tick species (Kollars, 1996; Kollars et al., 2000; Ostfeld et al., 1996). As suggested by Ostfeld et al. (1996), the important role of *P. leucopus* as a host for tick larvae may be influenced by its population density, as a higher number of rodents in a given area increases the likelihood of tick attachment. (. However, the fact that *P. leucopus* harbors a disproportionately higher number of larval ticks than other abundant sympatric hosts such as *S. hispidus* or *Perognathus merriami*, suggests a host-specific relationship between *Dermacentor* larvae and *P. leucopus*. This could also highlight the role of host life history traits in explaining the parasite load in addition to host density (Brunner and Ostfeld, 2008; Dallas and Foré, 2013).

Our results indicate that *Dermacentor* larval tick infestation in *P. leucopus* is dynamic over time, decreasing in late spring and early autumn, when environmental conditions are likely most detrimental to both rodents and ticks. This seasonal pattern may be influenced by the phenology of both the host and the tick. We captured reproducing *P. leucopus* individuals throughout all sampling months, particularly in September, October, and November, suggesting that the observed patterns are driven by larval activity rather than rodent activity. Notably, in some cases, a reduction in expected parasite load may also result from an increase in host population size (Dobson, 2014). As the number of rodents increases, individual parasite load decreases because the same number of ticks is distributed among a larger pool of hosts. However, in our study, the highest abundance of reproductive *P. leucopus* individuals was recorded in November, coinciding with the highest parasite loads predicted by our models. This pattern also aligns with findings from northern Florida, where *Dermacentor* larval activity increases from autumn through winter (McEnroe, 1979) and, in our study, extends into early spring. Although our results point out that larval activity is responsible for generating these patterns, year-round sampling of questing *Dermacentor* larvae is needed to confirm our hypothesis.

The infestation rate of *P. leucopus* was influenced not only by larval activity but also by the reproductive status and sex of the hosts. In general, reproductively active individuals carried a higher load of *Dermacentor* spp. Larvae. This pattern may be explained by increased mobility, as these individuals actively search for mates (Wolff and Cicirello, 1990). Since tick larvae remain spatially clustered after hatching, more mobile individuals are more likely to encounter and acquire parasites (Brunner and Ostfeld, 2008). Additionally, elevated steroid hormone production in reproductive individuals can suppress the immune system and reduce grooming behaviors, leading to a decreased anti-parasitic response (Grossman, 1985; Schalk and Forbes, 1997; Hughes and Randolph, 2001). Together, these factors explain the higher parasite load observed in actively reproducing *P. leucopus*.

Host sex also plays a crucial role in parasite load. In many species, including small mammals, parasite infestation tends to be male-biased (Wilson et al., 2002; Krasnov et al., 2012). Specifically, in *P. leucopus*, immature ticks have been shown to be attracted to male mice via chemical communication (Dallas and Foré, 2013), a pattern consistent with our general infestation results for this species. In contrast to *P. leucopus*, we found a slight female-biased infestation in *O. leucogaster*. Female-biased tick infestation is uncommon in rodent-tick studies (Schalk and Forbes, 1997; Krasnov et al., 2012), whereby, the mechanisms underlying this pattern remain poorly understood (Krasnov et al., 2012; Wilson et al., 2002). One possible explanation relates to species-specific behavior, as *Onychomys* are highly territorial (McCarty, 1978). Unlike other species, males do not overlap territories with females, whereas females may share burrows during the non-breeding season (Frank and Heske, 1992), potentially increasing their exposure to tick infestation. The small difference in expected parasite load between male and female *O. leucogaster* may be attributed to the fact that this species naturally occurs in low population densities and with low *Dermacentor* tick load (Galán et al., 2022; Stapp, 1999). Under certain conditions, even minor variations in parasite load can impact host survival or reproductive potential (Poulin, 1996; Wilson et al., 2002); however, this does not appear to be the case for tick parasite load in rodents (Hersh et al., 2014). Host body weight is often positively associated with the ectoparasite load, with larger animals having more ticks due to their bigger contact surfaces and hence reduced competition between ectoparasites (Randolph, 2004). Contrary to what we expected, *O. leucogaster* individuals with lower body mass had the highest tick loads. Interestingly, those individuals were also associated with areas of highly dense leaf litter cover. While acquired immunity can aid in explaining this inverse relationship—where parasite load decreases as individuals grow (Wilson et al., 2002)—habitat-specific dependence suggests it may not be the primary factor. Instead, our results may be linked to the differential habitat use by age classes in this rodent species. *O. leucogaster* is typically associated with open habitats with high prey richness, such as arthropods, and tends to avoid shrubs or woody areas (Stapp, 1997), which often have the highest percentages of leaf litter cover. Thus, the habitat use by *O. leucogaster* individuals could follow a despotic ideal distribution, where dominant (i.e., adult) individuals prevent the use of optimal habitat by subordinate or juvenile individuals (Brown, 1969; Calsbeek and Sinervo, 2002). The use of suboptimal habitats by juvenile individuals to avoid direct conflict or competition with adults has been reported for other rodent species (Doyle, 1990; Spencer et al., 2005). This phenomenon could be happening in our study given the territorial nature of *O. leucogaster*, and its interspecific aggression and even lethal behaviors (Pellis and Pellis, 1992; Ruffer, 1968). Thus in *O. leucogaster*, smaller individuals may use sub-optimal habitats to avoid competition with larger individuals, resulting in greater susceptibility to tick parasitism. This is supported by our results since parasite load in small individuals was not evident in areas with lower litter cover.

*Sigmodon hispidus* was the most commonly captured rodent in the study area and the second most infested with *Dermacentor* larvae. This result was expected, as *S. hispidus* has been identified as one of the primary hosts of *Dermacentor* ticks (Gage et al., 1992), due to its larger body size compared to the other rodent species, its preference for microhabitats favorable to questing immature tick stages, and its activity during day and night, which coincide with larval tick activity (Gage et al., 1992). Despite this, none of the host size or habitat variables measured in our study explained infestation in this species. A potential contributing factor is the highly aggregated distribution of larval ticks among the individuals in *S.hispidus* population (Gage et al., 1992). In fact, in our study, a single male harbored 30% (n = 25) of the *Dermacentor* larvae found in *S. hispidus*. This suggests that other host factors, such as health condition and immune response, may influence the infestation rates in this species, although these aspects were not assessed in our study. Another factor that may influenced our results is the high variability in infestation rates by *Dermacentor* larvae in *S. hispidus*, since other studies show it as an infrequent host for *Dermacentor* (Kollars, 1996). One explanation for this variability is the availability of other suitable hosts. Brunner and Ostfeld (2008) showed that when *Tamias striatus* (Eastern chipmunk) and *P. leucopus* inhabit the same area, the former draw part of the *Ixodes scapularis (Blacklegged tick)* larvae load from *P. leucopus*, its primary host. Since *Dermacentor* larvae exhibit some specificity for *P. leucopus* (Dallas et al., 2012), the presence of *P. leucopus* may similarly reduce the importance of *S. hispidus* as a potential host in areas where both species coexist. Given that *S. hispidus* is a relatively abundant rodent and a potential reservoir of tick-borne pathogens such as *Rickettsia* (Cumbie et al., 2020), we recommend continued monitoring of its populations and increased efforts to better understand the dynamics of tick infestation in this species, as published information remains limited.

In our study, we used tick presence (detection/non-detection) and load (tick abundance on each host) to evaluate their relationship with rodent hosts. Both approaches are commonly used to study host-parasite interactions across species (Kollars et al., 2000; Modarelli et al., 2020; Peralbo-Moreno et al., 2022; Stanko et al., 2007). Generally, presence data are easier and less costly to obtain, as they do not require a thorough search or count of each parasite on every host. However, abundance data is more powerful for detecting ecological patterns (Estrada and Arroyo, 2012; Shaw et al., 2018). That is, while both analyses yielded similar results, using parasite load allowed us to detect the effects of variables such as sex, or reproductive activity, and interactions between these variables with the month. The choice between load and presence depends on the logistical capacity and monitoring objectives of each study. Another important consideration is that both tick load and presence are subject to imperfect detection (Kéry and Royle, 2015), which, in the case of ticks, occurs at both the host level (ability to capture both uninfested and infected hosts) and the tick level (ability to detect ticks on each infested host) (MacKenzie et al., 2017). Hierarchical modeling approaches can formally account for imperfect detection, but they require specific sampling designs that we did not meet (Eads et al., 2013). Studies that use hierarchical modeling approaches could enhance the ability to detect the effects of host, habitat, or environmental variables on tick-host and tick-host-pathogen relationships (Eads et al., 2013; Lachish et al., 2012).

In conclusion, our results demonstrate how species-specific factors influenced *Dermacentor* larval tick presence and load in *P. leucopus* and *O. leucogaster* in a locality of southern Texas. In *P. leucopus*, a more generalist and common species, individual traits such as sex and reproductive status influenced tick parasite load, which was further conditioned by the activity of larval ticks and/or mice throughout the year. In contrast, in *O. leucogaster*, a more specialist species, tick parasitism was associated with territorial behaviors that affect habitat use and, consequently, determine its tick load. We were unable to identify predictors associated with the parasite load of *S. hispidus*; however, given its role in the ecology of tick-borne diseases, we recommend continued efforts to understand the factors that determine its parasite load. Our study contributes to a broader understanding of tick-rodent interactions and allows us to anticipate changes in parasitism patterns and manage wildlife health in response to environmental change.

## CRediT authorship contribution statement

**Gabriel P. Andrade-Ponce:** Writing – review & editing, Writing – original draft, Visualization, Validation, Methodology, Formal analysis, Data curation, Conceptualization. **Brandi G. Giles:** Writing – review & editing, Writing – original draft, Methodology, Investigation, Formal analysis, Data curation. **Brent C. Newman:** Writing – review & editing, Writing – original draft, Resources, Investigation, Data curation, Conceptualization. **Andrés M. López-Pérez:** Writing – review & editing, Validation. **Cord B. Eversole:** Writing – review & editing, Writing – original draft, Resources, Project administration, Investigation, Funding acquisition, Data curation, Conceptualization.

## Note

Supplementary data associated with this article'

## Declaration of generative AI and AI-assisted technologies in the writing process

During the preparation of this work the authors used ChatGPT in order to improve the grammar and wording of the text. After using this tool/service, the author(s) reviewed and edited the content as needed and take(s) full responsibility for the content of the publication.

## Declaration of competing interest

The authors declare that they have no known competing financial interests or personal relationships that could have appeared to influence the work reported in this paper.
