# Supplementary material for: Different drivers, same tick: Effect of host traits, habitat, and climate on the infestation of three rodent species by larval Dermacentor ticks
Source: Int J Parasitol Parasites Wildl. 2025 Mar 7;26:101054. doi: 10.1016/j.ijppaw.2025.101054 (PMC11929885; doi:10.1016/j.ijppaw.2025.101054)
Supplement: Multimedia component 1 [file mmc1.zip › Supplementary File 1_revised.html]

Statistical modeling process for the analysis of the prevalence of Dermacentor larvae in two species of small rodents.


## Table of contents

- Packages
- Functions
- Host level data
- Description of variables
- Modeling
  - ***Onychomys leucogaster***
    - ***Dermacentor*** **larvae**
  - ***Peromyscus leucopus***
    - ***Dermacentor*** larvae
  - ***Sigmodon hispidus***
    - ***Dermacentor*** larvae
- Final plots

# Statistical modeling process for the analysis of the prevalence of Dermacentor larvae in two species of small rodents.

 Code

- Show All Code
- Hide All Code
- ---
- View Source

# Packages

List of packages used

Code

```
library(tidyverse)
library(glmmTMB)
library(performance)
library(modelbased)
library(DHARMa)
library(patchwork)
library(parameters)
library(AICcmodavg)
library(kableExtra)
library(ggeffects)
```

# Functions

Load custom functions for data organization, analysis and creation of graphs.

Code

```
source("Code/Functions_host.R")
```

# Host level data

Load the main data table

Code

```
T_coords <- read.csv("Data/Tcoords.csv") # coordinates table

#Main data table
H_data <- read.csv("Data/host_level.csv") %>%
  select(-X, -Count, -ID, -Trap, -Trapline) %>% 
  #Select only the rodents with available data
  filter(SM_Sp %in% c("ONLE", "PELE", "SIHI")) %>%
  #Drop table mising values
  drop_na() %>%
  #Create year as factor
  mutate(year= as.factor(year),
         Month= as.factor(Month)) %>%
  #Join with coordinates
  left_join(T_coords) %>% 
  #Create transect ID
  mutate(T_group= as.factor(gsub("[^0-9]", "", Transect) ),
         # Transform abundances to 1 and 0
         n= if_else(n>0, 1, n))
```

# Description of variables

Description of the used variables

Description of the variables used to model tick presence and load on small mammal hosts in South Texas, USA.

| Variable (Code) | Description | Variable type | Hypothesis | Expected relation |
| --- | --- | --- | --- | --- |
| Tick prevalence | Detection or non-detection of ticks in each rodent individual. | Response variable | NA | NA |
| Tick load | The number of tick counts for each rodent individual captured | Response variable | NA | NA |
| Body weight (g) (Wght) | The weight of each rodent individual captured (g) | Host explanatory variable - Continuum | Hosts with greater body size represent more habitat and resources for ticks (Kuris et al., 1980; Poulin, 2004). Therefore, bigger individuals will favor greater presence and number of ticks. | Linear / Quadratic |
| Sex (Sex) | The sex of each rodent captured | Host explanatory variable - Categorical : Male (M), Female (F) | Male mice tend to move more in the landscape compared to females. Therefore, males are expected to have higher tick burdens than females (Butler et al., 2020; Ostfeld et al., 1996). | NA |
| Reproductive status (Rep) | The reproductive status of each captured rodent. Reproductive and non-reproductive | Host explanatory variable - Categorical: non-reproductive (N), reproductive (Y) | The hormonal profile and the higher activity of sexually active mice make them more susceptible to a higher parasite load. Therefore, we expect that sexually active mice will have a higher chance of having a greater tick load (Ostfeld et al., 1996; Schmidt et al., 1999). | NA |
| Average litter cover (Avg\_LC) | Percent (%) presence of litter cover in each transect | Habitat explanatory variable - Continuum | Higher numbers of questing tick larvae are associated with areas with higher leaf litter cover, as these locations allow them to avoid adverse environmental conditions (Sonenshine, 2005). We expect that individual mice captured in areas of higher cover and depth of leaf litter will have higher tick larvae burdens. | Linear |
| Average litter depth (Avg\_LD) | Average of litter depth in each transect (cm) | Habitat explanatory variable - Continuum | NA | Linear |
| Average Herbaceous material (Avg\_HC) | Percent (%) presence of Herbaceous in each transect | Habitat explanatory variable - Continuum | Herbaceous cover favors microclimatic conditions for tick larval survival and therefore tick abundance (Sonenshine and Stout, 1968). We expect that mice captured in areas with higher grass cover will have higher tick burdens. | Linear |
| Average Canopy Cover (Avg\_CC) | Percent (%) of canopy coverture in each transect | Habitat explanatory variable - Continuum | Greater canopy cover increases humidity and reduces direct sunlight on the ground, which favors climatic conditions for tick larval development (Ginsberg et al., 2020; Leal et al., 2018; Zamora et al., 2020). We expect that individual mice captured in areas with higher canopy cover will have higher tick burdens. | Linear |
| Average vegetation height (Avg\_VH) | Average vegetation height for each transects (cm) | Habitat explanatory variable - Continuum | Tick larvae prefer vegetation with a height of less than 50 cm because it favors climatic conditions for their development (Prusinski et al., 2006). For this reason, we expect that captured mice will have higher tick loads in areas with lower vegetation height. | Linear/ Quadratic |
| Rain (Rain) | Rainfall measure (mm) for each day of the survey with Hobo data loggers | Weather explanatory variable - Continuum | Rainfall can negatively affect the development of tick larvae by washing and flooding microhabitats. In addition, a correlation has been reported between drier areas and periods of the year and tick larval abundance (Randolph and Storey, 1999). Therefore, we expect that rodent captured during periods of higher rainfall will have a lower parasite load. | Linear |
| Temperature average (Temp) | Temperature (C) measure for each day of the survey with Hobo data loggers | Weather explanatory variable - Continuum | Ticks show a peak of activity during warm weather periods and areas (Gilbert, 2010). However, extreme temperature or drought conditions may affect larval survival by desiccation (Teel et al., 2010). Therefore, we expect that mice captured during periods of very low or very high temperatures will have lower tick burdens. | Linea/ Quadratic |
| Season (Season) | The season in which the survey was conducted | Weather explanatory variable - Categorical: Spring (, Fall and Winter | Ticks and their larvae exhibit seasonal peaks of activity (Brunner and Ostfeld, 2008). In the southern United States, tick larval activity patterns are greatest during the spring because weather conditions are more favorable than summer and winter (Chan and Kaufman, 2009; McEnroe, 1979). Therefore, we expect that rodent captured during the spring survey will have a higher tick load. | NA |
| Month (Month) | Month of the year in which the individuals were captured | Time control explanatory variable - Categorical: Month of the year represented in integer form 1: January to 12: December | Variable used as a control for the time effect. The month factor allows to identify influences of phenological, environmental or climatic dynamics that are not captured by the other variables used. | NA |
| Transect (Transect) | ID of the transect in which each individual was captured | Random effects variable | NA | NA |

# Modeling

## ***Onychomys leucogaster***

### ***Dermacentor*** **larvae**

Code

```
ONLE_D <- H_data %>% 
  filter(SM_Sp == "ONLE" & # Filter the rodent species
           Tick_gen %in% c("Dermacentor", "No tick") & # filter de tick sp
           Tick_Age %in% c("Larvae", "No tick") ) # filter the tick age
```

#### Random effects

To evaluate the random effects structure, models with all fixed variables and possible random structures were ranked: transect ID random intercept and a model with no random effects. Models were ranked using the Akaike information criterion corrected for small samples (AICc).

Code

```
OD_random <-random_eff(ONLE_D, binomial())
kbl(compare_performance(OD_random, metrics = "AICc"),
    digits = 3) %>% 
  kable_styling(bootstrap_options = c("striped", "hover"), full_width = F)
```

| Name | Model | AICc | AICc\_wt |
| --- | --- | --- | --- |
| Transect | glmmTMB | 80.804 | 0.337 |
| norandom | glmmTMB | 79.454 | 0.663 |

Subsequently, the residuals of the selected model are inspected.

Code

```
OD_norandom_res <- simulateResiduals(OD_random$norandom, plot = T)
```

Inspection of model residuals without random effects

#### Evaluate any spatial correlation issues

Inspection of the spatial autocorrelation of residuals of the selected model.

Code

```
OD_audf <- get_sau_data(OD_norandom_res, ONLE_D)
OD_groups <- split(OD_audf, OD_audf$survey)
OD_corelog <- map(OD_groups, get_correlog) %>% compact()
OD_corelogbase <- map(OD_corelog, get_correlog_base)
OD_corelogplot <- map(OD_corelogbase, get_correlogplot)
OD_corelogplot$Fall_2019
OD_corelogplot$Fall_2020
OD_corelogplot$Winter_2019
OD_corelogplot$Winter_2020
```

```
500  of  5000 
1000  of  5000 
1500  of  5000 
2000  of  5000 
2500  of  5000 
3000  of  5000 
3500  of  5000 
4000  of  5000 
4500  of  5000 
5000  of  5000 
500  of  5000 
1000  of  5000 
1500  of  5000 
2000  of  5000 
2500  of  5000 
3000  of  5000 
3500  of  5000 
4000  of  5000 
4500  of  5000 
5000  of  5000 
500  of  5000 
1000  of  5000 
1500  of  5000 
2000  of  5000 
2500  of  5000 
3000  of  5000 
3500  of  5000 
4000  of  5000 
4500  of  5000 
5000  of  5000 
500  of  5000 
1000  of  5000 
1500  of  5000 
2000  of  5000 
2500  of  5000 
3000  of  5000 
3500  of  5000 
4000  of  5000 
4500  of  5000 
5000  of  5000
```

-“Fall 2019 correlogram” -“Fall 2020 correlogram” -“Winter 2019 correlogram” -“Winter 2020 correlogram”

-“Fall 2019 correlogram” -“Fall 2020 correlogram” -“Winter 2019 correlogram” -“Winter 2020 correlogram”

-“Fall 2019 correlogram” -“Fall 2020 correlogram” -“Winter 2019 correlogram” -“Winter 2020 correlogram”

-“Fall 2019 correlogram” -“Fall 2020 correlogram” -“Winter 2019 correlogram” -“Winter 2020 correlogram”

According to the inspection of the residuals, the selected model showed adequate goodness-of-fit and non spatial autocorrelation problems.

#### Host variables

Univariate models were created for the selection of host variables. The models were ranked using AICc. Variables from models with Delta <2 were used in the final model selection.

Code

```
OD_H <- get_H_models(ONLE_D, family =binomial(),zi = FALSE)
  
OD_H_sel <-aictab(OD_H, sort = T)

kbl(OD_H_sel,
    caption = "Model selection table for host variables",
    digits = 3) %>% 
  kable_styling(bootstrap_options = c("striped", "hover"), full_width = F)
```

Model selection table for host variables

|  | Modnames | K | AICc | Delta\_AICc | ModelLik | AICcWt | LL | Cum.Wt |
| --- | --- | --- | --- | --- | --- | --- | --- | --- |
| 4 | Rep | 2 | 67.211 | 0.000 | 1.000 | 0.377 | -31.556 | 0.377 |
| 3 | Sex | 2 | 67.339 | 0.128 | 0.938 | 0.354 | -31.620 | 0.731 |
| 1 | Weight | 2 | 68.573 | 1.362 | 0.506 | 0.191 | -32.237 | 0.921 |
| 2 | Weight2 | 3 | 70.343 | 3.132 | 0.209 | 0.079 | -32.071 | 1.000 |

#### Habitat variables

Univariate models were created for the selection of habitat variables. The models were ranked using AICc. Variables from models with Delta <2 were used in the final model selection.

Code

```
OD_Ha <- get_Hamodels(ONLE_D, family = binomial(),zi = FALSE)
  
OD_Ha_sel <- aictab(OD_Ha, sort = T)

  kbl(OD_Ha_sel,
    caption = "Model selection table for habitat variables",
    digits = 3) %>% 
  kable_styling(bootstrap_options = c("striped", "hover"), full_width = F)
```

Model selection table for habitat variables

|  | Modnames | K | AICc | Delta\_AICc | ModelLik | AICcWt | LL | Cum.Wt |
| --- | --- | --- | --- | --- | --- | --- | --- | --- |
| 6 | Litter\_Cover | 2 | 59.589 | 0.000 | 1.000 | 0.873 | -27.745 | 0.873 |
| 4 | Vegetation\_height | 2 | 65.097 | 5.508 | 0.064 | 0.056 | -30.499 | 0.929 |
| 5 | Vegetation\_height2 | 3 | 65.839 | 6.250 | 0.044 | 0.038 | -29.820 | 0.967 |
| 1 | Litter\_depth | 2 | 67.882 | 8.293 | 0.016 | 0.014 | -31.891 | 0.981 |
| 3 | Canopy\_Cover | 2 | 68.633 | 9.044 | 0.011 | 0.009 | -32.267 | 0.991 |
| 2 | Herbaceus\_Cover | 2 | 68.638 | 9.049 | 0.011 | 0.009 | -32.270 | 1.000 |

#### Weather variables

Univariate models were created for the selection of weather variables. The models were ranked using AICc. Variables from models with Delta <2 were used in the final model selection.

Code

```
OD_W <- get_Wmodels(ONLE_D, family = binomial(),zi = FALSE)

OD_W_sel <- aictab(OD_W, sort = T)

  kbl(OD_W_sel,
    caption = "Model selection table for  weather variables",
    digits = 3) %>% 
  kable_styling(bootstrap_options = c("striped", "hover"), full_width = F)
```

Model selection table for weather variables

|  | Modnames | K | AICc | Delta\_AICc | ModelLik | AICcWt | LL | Cum.Wt |
| --- | --- | --- | --- | --- | --- | --- | --- | --- |
| 3 | Rain | 2 | 66.056 | 0.000 | 1.000 | 0.558 | -30.978 | 0.558 |
| 4 | Season | 3 | 67.828 | 1.773 | 0.412 | 0.230 | -30.814 | 0.787 |
| 1 | Temp | 2 | 68.602 | 2.547 | 0.280 | 0.156 | -32.252 | 0.944 |
| 2 | Temp2 | 3 | 70.636 | 4.580 | 0.101 | 0.056 | -32.218 | 1.000 |

#### Final selection

With the variables selected above, we generated candidate models of their possible interactions. We ranked them using AICc.

Code

```
OD_models <- list(
OD_m1 <- glmmTMB(n~ Rep,                       family =binomial, data = ONLE_D),
OD_m2 <- glmmTMB(n~ Rep+ Sex,                  family =binomial, data = ONLE_D),
OD_m3 <- glmmTMB(n~ Rep+ Wght,                 family =binomial, data = ONLE_D),
OD_m4 <- glmmTMB(n~ Rep+ Avg_LC,               family =binomial, data = ONLE_D),
OD_m5 <- glmmTMB(n~ Rep+ Season,               family =binomial, data = ONLE_D),
OD_m6 <- glmmTMB(n~ Rep* Sex,                  family =binomial, data = ONLE_D),
OD_m7 <- glmmTMB(n~ Rep* Wght,                 family =binomial, data = ONLE_D),
OD_m8 <- glmmTMB(n~ Rep* Avg_LC,               family =binomial, data = ONLE_D),
OD_m9 <- glmmTMB(n~ Rep* Season,               family =binomial, data = ONLE_D),

OD_m10 <- glmmTMB(n~ Sex,                       family =binomial, data = ONLE_D),
OD_m11 <- glmmTMB(n~ Sex+ Wght,                 family =binomial, data = ONLE_D),
OD_m12 <- glmmTMB(n~ Sex+ Avg_LC,               family =binomial, data = ONLE_D),
OD_m13 <- glmmTMB(n~ Sex+ Season,               family =binomial, data = ONLE_D),
OD_m14 <- glmmTMB(n~ Sex* Wght,                 family =binomial, data = ONLE_D),
OD_m15 <- glmmTMB(n~ Sex* Avg_LC,               family =binomial, data = ONLE_D),
OD_m16 <- glmmTMB(n~ Sex* Season,               family =binomial, data = ONLE_D),

OD_m17 <- glmmTMB(n~ Wght,                       family =binomial, data = ONLE_D),
OD_m18 <- glmmTMB(n~ Wght+ Avg_LC,               family =binomial, data = ONLE_D),
OD_m19 <- glmmTMB(n~ Wght+ Season,               family =binomial, data = ONLE_D),
OD_m20 <- glmmTMB(n~ Wght* Avg_LC,               family =binomial, data = ONLE_D),
OD_m21 <- glmmTMB(n~ Wght* Season,               family =binomial, data = ONLE_D),


OD_m22 <- glmmTMB(n~ Avg_LC,                    family =binomial, data = ONLE_D),
OD_m23 <- glmmTMB(n~ Avg_LC+ Season,            family =binomial, data = ONLE_D),
OD_m24 <- glmmTMB(n~ Avg_LC* Season,            family =binomial, data = ONLE_D),

OD_m25 <- glmmTMB(n~ Rain,                    family =binomial, data = ONLE_D),
OD_m26 <- glmmTMB(n~ Rain+ Season,            family =binomial, data = ONLE_D),
OD_m27 <- glmmTMB(n~ Rain* Season,            family =binomial, data = ONLE_D),

OD_m28 <- glmmTMB(n~ Season,                    family =binomial, data = ONLE_D),

OD_m29 <- glmmTMB(n~  1,                       family =binomial, data = ONLE_D),

OD_m30 <- glmmTMB(n~ Month,                    family =binomial, data = ONLE_D),
OD_m31 <- glmmTMB(n~ Month*Rep,                family =binomial, data = ONLE_D),
OD_m32 <- glmmTMB(n~ Month*Sex,                family =binomial, data = ONLE_D)

)


OD_sel <- aictab(OD_models, sort = F)

OD_sel_table <- get_table_models(OD_models, OD_sel, "Prevalence")


  kbl(OD_sel_table,
    caption = "Final model selection to identify drivers influencing tick presence in Onychomys leucogaster",
    digits = 2) %>% 
  kable_styling(bootstrap_options = c("striped", "hover"), full_width = F)
```

Final model selection to identify drivers influencing tick presence in Onychomys leucogaster

| Formula | Modnames | K | AICc | Delta\_AICc | ModelLik | AICcWt | LL |
| --- | --- | --- | --- | --- | --- | --- | --- |
| Tick Prevalence ~ Avg\_LC | Mod22 | 2 | 59.59 | 0.00 | 1.00 | 0.20 | -27.74 |
| Tick Prevalence ~ Wght \* Avg\_LC | Mod20 | 4 | 59.85 | 0.26 | 0.88 | 0.18 | -25.76 |
| Tick Prevalence ~ Rep + Avg\_LC | Mod4 | 3 | 60.30 | 0.72 | 0.70 | 0.14 | -27.05 |
| Tick Prevalence ~ Sex + Avg\_LC | Mod12 | 3 | 60.40 | 0.81 | 0.67 | 0.14 | -27.10 |
| Tick Prevalence ~ Sex \* Avg\_LC | Mod15 | 4 | 60.89 | 1.31 | 0.52 | 0.11 | -26.28 |
| Tick Prevalence ~ Wght + Avg\_LC | Mod18 | 3 | 61.45 | 1.86 | 0.39 | 0.08 | -27.62 |
| Tick Prevalence ~ Rep \* Avg\_LC | Mod8 | 4 | 62.32 | 2.73 | 0.25 | 0.05 | -26.99 |
| Tick Prevalence ~ Avg\_LC + Season | Mod23 | 4 | 63.19 | 3.60 | 0.17 | 0.03 | -27.43 |
| Tick Prevalence ~ Avg\_LC \* Season | Mod24 | 6 | 65.49 | 5.90 | 0.05 | 0.01 | -26.39 |
| Tick Prevalence ~ Rain | Mod25 | 2 | 66.06 | 6.47 | 0.04 | 0.01 | -30.98 |
| Tick Prevalence ~ 1 | Mod29 | 1 | 66.58 | 6.99 | 0.03 | 0.01 | -32.27 |
| Tick Prevalence ~ Rep + Sex | Mod2 | 3 | 66.97 | 7.38 | 0.03 | 0.01 | -30.38 |
| Tick Prevalence ~ Rep | Mod1 | 2 | 67.21 | 7.62 | 0.02 | 0.00 | -31.56 |
| Tick Prevalence ~ Sex | Mod10 | 2 | 67.34 | 7.75 | 0.02 | 0.00 | -31.62 |
| Tick Prevalence ~ Rain + Season | Mod26 | 4 | 67.67 | 8.09 | 0.02 | 0.00 | -29.67 |
| Tick Prevalence ~ Season | Mod28 | 3 | 67.83 | 8.24 | 0.02 | 0.00 | -30.81 |
| Tick Prevalence ~ Rep + Season | Mod5 | 4 | 68.12 | 8.53 | 0.01 | 0.00 | -29.89 |
| Tick Prevalence ~ Rep \* Sex | Mod6 | 4 | 68.39 | 8.81 | 0.01 | 0.00 | -30.03 |
| Tick Prevalence ~ Wght | Mod17 | 2 | 68.57 | 8.98 | 0.01 | 0.00 | -32.24 |
| Tick Prevalence ~ Sex + Season | Mod13 | 4 | 69.17 | 9.58 | 0.01 | 0.00 | -30.42 |
| Tick Prevalence ~ Sex + Wght | Mod11 | 3 | 69.22 | 9.63 | 0.01 | 0.00 | -31.51 |
| Tick Prevalence ~ Rep + Wght | Mod3 | 3 | 69.28 | 9.69 | 0.01 | 0.00 | -31.54 |
| Tick Prevalence ~ Rep \* Wght | Mod7 | 4 | 69.44 | 9.85 | 0.01 | 0.00 | -30.55 |
| Tick Prevalence ~ Rain \* Season | Mod27 | 5 | 69.85 | 10.26 | 0.01 | 0.00 | -29.67 |
| Tick Prevalence ~ Wght + Season | Mod19 | 4 | 69.89 | 10.30 | 0.01 | 0.00 | -30.78 |
| Tick Prevalence ~ Month | Mod30 | 7 | 69.96 | 10.37 | 0.01 | 0.00 | -27.49 |
| Tick Prevalence ~ Rep \* Season | Mod9 | 6 | 70.13 | 10.54 | 0.01 | 0.00 | -28.71 |
| Tick Prevalence ~ Wght \* Season | Mod21 | 6 | 70.76 | 11.17 | 0.00 | 0.00 | -29.02 |
| Tick Prevalence ~ Sex \* Season | Mod16 | 5 | 70.83 | 11.25 | 0.00 | 0.00 | -30.16 |
| Tick Prevalence ~ Sex \* Wght | Mod14 | 4 | 70.91 | 11.32 | 0.00 | 0.00 | -31.29 |
| Tick Prevalence ~ Month \* Rep | Mod31 | 12 | 78.68 | 19.09 | 0.00 | 0.00 | -25.94 |
| Tick Prevalence ~ Month \* Sex | Mod32 | 12 | 78.75 | 19.16 | 0.00 | 0.00 | -25.97 |

We inspected the 85% confidence intervals of the regression coefficients of the selected models.

Code

```
OD_final_best <- list(OD_m22=OD_m22, OD_m20=OD_m20, OD_m4=OD_m4, OD_m12=OD_m12, OD_m15=OD_m15, OD_m18=OD_m18)

OD_final_best_ci <- map2_df(names(OD_final_best), OD_final_best, get_ci)

 kbl(OD_final_best_ci,
    caption = "Selected models confidence interval table",
    digits = 2) %>% 
  kable_styling(bootstrap_options = c("striped", "hover"), full_width = F)
```

Selected models confidence interval table

| Parameter | Coefficient | CI\_low | CI\_high | Model | Informative |
| --- | --- | --- | --- | --- | --- |
| Avg\_LC | 0.08 | 0.04 | 0.12 | OD\_m22 | yes |
| Wght | 0.21 | 0.07 | 0.34 | OD\_m20 | yes |
| Avg\_LC | 0.33 | 0.17 | 0.49 | OD\_m20 | yes |
| Wght:Avg\_LC | -0.01 | -0.01 | 0.00 | OD\_m20 | yes |
| RepY | 0.88 | -0.23 | 1.99 | OD\_m4 | no |
| Avg\_LC | 0.08 | 0.04 | 0.12 | OD\_m4 | yes |
| SexM | -0.93 | -2.18 | 0.32 | OD\_m12 | no |
| Avg\_LC | 0.08 | 0.04 | 0.12 | OD\_m12 | yes |
| SexM | -4.67 | -10.17 | 0.83 | OD\_m15 | no |
| Avg\_LC | 0.06 | 0.02 | 0.11 | OD\_m15 | yes |
| SexM:Avg\_LC | 0.10 | -0.03 | 0.23 | OD\_m15 | no |
| Wght | 0.02 | -0.04 | 0.08 | OD\_m18 | no |
| Avg\_LC | 0.08 | 0.04 | 0.12 | OD\_m18 | yes |

Code

```
ci_plot(OD_final_best_ci)+ labs(title= "Onychomys leucogaster models 85%CI")
```

Model 22 (Tick Prevalence ~ Avg\_LC) and 20 (Tick Prevalence ~ Wght \* Avg\_LC ) contain informative estimations

#### Selected models

Subsequently, we inspect the residuals of the models whose confidence intervals do not overlap 0.

Code

```
OD_res <- lapply(list(OD_m22, OD_m20), simulateResiduals, plot= TRUE)
```

Residual inspection of model Tick Prevalence ~ Avg\_LC

Residual inspection of model Tick Prevalence ~ Wght \* Avg\_LC

We build the summary table of the selected models

Code

```
OD_1_sum <- model_parameters(OD_m20, digits=2, ci= 0.85) %>% 
  mutate(Family= "Bernoulli",
         Model= "Model 20") %>% 
  select(-SE, -z, -df_error)
OD_2_sum <- model_parameters(OD_m22, digits=2, ci= 0.85) %>% 
  mutate(Family= "Bernoulli",
         Model= "Model 22") %>% 
  select(-SE, -z, -df_error)

kbl(rbind(OD_1_sum, OD_2_sum),
    caption = "O. leocogaster - Dermacentor",
    digits = 3) %>% 
  kable_styling(bootstrap_options = c("striped", "hover"), full_width = F)
```

O. leocogaster - Dermacentor

| Parameter | Coefficient | CI | CI\_low | CI\_high | p | Effects | Family | Model |
| --- | --- | --- | --- | --- | --- | --- | --- | --- |
| (Intercept) | -12.956 | 0.85 | -18.682 | -7.230 | 0.001 | fixed | Bernoulli | Model 20 |
| Wght | 0.205 | 0.85 | 0.072 | 0.339 | 0.027 | fixed | Bernoulli | Model 20 |
| Avg\_LC | 0.327 | 0.85 | 0.168 | 0.485 | 0.003 | fixed | Bernoulli | Model 20 |
| Wght:Avg\_LC | -0.006 | 0.85 | -0.011 | -0.002 | 0.023 | fixed | Bernoulli | Model 20 |
| (Intercept) | -4.838 | 0.85 | -6.276 | -3.400 | 0.000 | fixed | Bernoulli | Model 22 |
| Avg\_LC | 0.079 | 0.85 | 0.040 | 0.119 | 0.004 | fixed | Bernoulli | Model 22 |

#### Prediction plots

Code

```
OD_Pred1 <- ggeffect(OD_m22, 
                     terms = c("Avg_LC"), 
                     ci_level = 0.85)

OD_Pred2 <- ggeffect(OD_m20, 
                     terms = c("Wght", "Avg_LC"), 
                     ci_level = 0.85)


OD_pred1_plot <- conpred_plot(OD_Pred1, title = NULL,
                              "Average litter cover (%)",
                              ONLE_D, var = Avg_LC,
                              fill = "#A2CD5A")+
  labs(y= "Tick presence probability")

OD_pred2_plot <- ggplot()+
  geom_ribbon(data= OD_Pred2, aes(x= x, y=predicted,
                                  ymin=conf.low, ymax=conf.high,
                                  fill=group), alpha= 0.4)+
  geom_line(data= OD_Pred2, aes(x= x, y= predicted, col= group),
            linewidth= 1)+
  labs(x= "Host weight (g)", y= "Tick presence probability",
       fill= "Average litter cover (%)",
       col= "Average litter cover (%)")+
    guides(col= guide_legend(position = "inside"),
         fill= guide_legend(position = "inside"))+
    scale_color_viridis_d()+
    scale_fill_viridis_d()+
    theme_bw(base_size = 12)+
    theme(legend.position.inside = c(0.7, 0.8),
          legend.background = element_blank())

OD_pred1_plot
OD_pred2_plot
```

-“Prediction plot of model Tick Prevalence ~ Avg\_LC” -“Prediction plot of model Tick Prevalence ~ Wght \* Avg\_LC”

-“Prediction plot of model Tick Prevalence ~ Avg\_LC” -“Prediction plot of model Tick Prevalence ~ Wght \* Avg\_LC”

## ***Peromyscus leucopus***

### ***Dermacentor*** larvae

Code

```
PELE_D <- H_data %>% 
  filter(SM_Sp == "PELE" & 
           Tick_gen %in% c("Dermacentor", "No tick") & 
           Tick_Age %in% c("Larvae", "No tick") )
```

#### Random effects

To evaluate the random effects structure, models with all fixed variables and possible random structures were ranked: transect ID random intercept and a model with no random effects. Models were ranked using the Akaike information criterion corrected for small samples (AICc).

Code

```
PD_random <- random_eff(PELE_D, binomial())
kbl(compare_performance(PD_random, metrics = "AICc"),
    digits = 3) %>% 
  kable_styling(bootstrap_options = c("striped", "hover"), full_width = F)
```

| Name | Model | AICc | AICc\_wt |
| --- | --- | --- | --- |
| Transect | glmmTMB | 378.886 | 0.257 |
| norandom | glmmTMB | 376.759 | 0.743 |

Residual inspection

Code

```
PD_norandom_res <- simulateResiduals(PD_random$norandom, plot = T)
```

#### Evaluate any spatial correlation issues

Code

```
PD_audf <- get_sau_data(PD_norandom_res, PELE_D)
PD_groups <- split(PD_audf, PD_audf$survey)
PD_corelog <- map(PD_groups, get_correlog)
PD_corelogbase <- map(PD_corelog, get_correlog_base)
PD_corelogplot <- map(PD_corelogbase, get_correlogplot)
PD_corelogplot
```

```
500  of  5000 
1000  of  5000 
1500  of  5000 
2000  of  5000 
2500  of  5000 
3000  of  5000 
3500  of  5000 
4000  of  5000 
4500  of  5000 
5000  of  5000 
500  of  5000 
1000  of  5000 
1500  of  5000 
2000  of  5000 
2500  of  5000 
3000  of  5000 
3500  of  5000 
4000  of  5000 
4500  of  5000 
5000  of  5000 
500  of  5000 
1000  of  5000 
1500  of  5000 
2000  of  5000 
2500  of  5000 
3000  of  5000 
3500  of  5000 
4000  of  5000 
4500  of  5000 
5000  of  5000 
500  of  5000 
1000  of  5000 
1500  of  5000 
2000  of  5000 
2500  of  5000 
3000  of  5000 
3500  of  5000 
4000  of  5000 
4500  of  5000 
5000  of  5000 
500  of  5000 
1000  of  5000 
1500  of  5000 
2000  of  5000 
2500  of  5000 
3000  of  5000 
3500  of  5000 
4000  of  5000 
4500  of  5000 
5000  of  5000
```

```
$Fall_2019
```

-“Fall 2019 correlogram” -“Fall 2020 correlogram” -“Spring 2020 correlogram” -“Winter 2019 correlogram” -“Winter 2020 correlogram”

```
$Fall_2020
```

-“Fall 2019 correlogram” -“Fall 2020 correlogram” -“Spring 2020 correlogram” -“Winter 2019 correlogram” -“Winter 2020 correlogram”

```
$Spring_2020
```

-“Fall 2019 correlogram” -“Fall 2020 correlogram” -“Spring 2020 correlogram” -“Winter 2019 correlogram” -“Winter 2020 correlogram”

```
$Winter_2019
```

-“Fall 2019 correlogram” -“Fall 2020 correlogram” -“Spring 2020 correlogram” -“Winter 2019 correlogram” -“Winter 2020 correlogram”

```
$Winter_2020
```

-“Fall 2019 correlogram” -“Fall 2020 correlogram” -“Spring 2020 correlogram” -“Winter 2019 correlogram” -“Winter 2020 correlogram”

According to the inspection of the residuals, the selected model showed adequate goodness-of-fit and non spatial autocorrelation problems.

#### Host variables

Univariate models were created for the selection of host variables. The models were ranked using AICc. Variables from models with Delta <2 were used in the final model selection.

Code

```
PD_H <-  get_H_models(PELE_D, family = binomial(), zi= FALSE)

PD_H_sel <- aictab(PD_H, sort = T)
kbl(PD_H_sel,
    caption = "Model selection table for host variables",
    digits = 3) %>% 
  kable_styling(bootstrap_options = c("striped", "hover"), full_width = F)
```

Model selection table for host variables

|  | Modnames | K | AICc | Delta\_AICc | ModelLik | AICcWt | LL | Cum.Wt |
| --- | --- | --- | --- | --- | --- | --- | --- | --- |
| 2 | Weight2 | 3 | 407.755 | 0.000 | 1.000 | 0.383 | -200.841 | 0.383 |
| 4 | Rep | 2 | 408.718 | 0.963 | 0.618 | 0.237 | -202.341 | 0.619 |
| 3 | Sex | 2 | 409.117 | 1.362 | 0.506 | 0.194 | -202.540 | 0.813 |
| 1 | Weight | 2 | 409.190 | 1.435 | 0.488 | 0.187 | -202.577 | 1.000 |

#### Habitat variables

Univariate models were created for the selection of habitat variables. The models were ranked using AICc. Variables from models with Delta <2 were used in the final model selection.

Code

```
PD_Ha <- get_Hamodels(PELE_D, family = binomial, zi= FALSE)

PD_Ha_sel <- aictab(PD_Ha, sort = T)

  kbl(PD_Ha_sel,
    caption = "Model selection table for habitat variables",
    digits = 3) %>% 
  kable_styling(bootstrap_options = c("striped", "hover"), full_width = F)
```

Model selection table for habitat variables

|  | Modnames | K | AICc | Delta\_AICc | ModelLik | AICcWt | LL | Cum.Wt |
| --- | --- | --- | --- | --- | --- | --- | --- | --- |
| 5 | Vegetation\_height2 | 3 | 405.023 | 0.000 | 1.000 | 0.364 | -199.475 | 0.364 |
| 4 | Vegetation\_height | 2 | 405.359 | 0.337 | 0.845 | 0.307 | -200.661 | 0.671 |
| 6 | Litter\_Cover | 2 | 405.837 | 0.814 | 0.666 | 0.242 | -200.900 | 0.913 |
| 3 | Canopy\_Cover | 2 | 408.841 | 3.818 | 0.148 | 0.054 | -202.402 | 0.967 |
| 1 | Litter\_depth | 2 | 411.177 | 6.154 | 0.046 | 0.017 | -203.570 | 0.983 |
| 2 | Herbaceus\_Cover | 2 | 411.184 | 6.161 | 0.046 | 0.017 | -203.574 | 1.000 |

#### Weather variables

Univariate models were created for the selection of weather variables. The models were ranked using AICc. Variables from models with Delta <2 were used in the final model selection.

Code

```
PD_W <- get_Wmodels(PELE_D, family = binomial(), zi= FALSE)

PD_W_sel <- aictab(PD_W, sort = T)
kbl(PD_W_sel,
    caption = "Model selection table for habitat variables",
    digits = 3) %>% 
  kable_styling(bootstrap_options = c("striped", "hover"), full_width = F)
```

Model selection table for habitat variables

|  | Modnames | K | AICc | Delta\_AICc | ModelLik | AICcWt | LL | Cum.Wt |
| --- | --- | --- | --- | --- | --- | --- | --- | --- |
| 2 | Temp2 | 3 | 388.364 | 0.000 | 1.000 | 0.733 | -191.145 | 0.733 |
| 1 | Temp | 2 | 390.390 | 2.026 | 0.363 | 0.266 | -193.177 | 1.000 |
| 4 | Season | 3 | 403.738 | 15.374 | 0.000 | 0.000 | -198.832 | 1.000 |
| 3 | Rain | 2 | 405.934 | 17.570 | 0.000 | 0.000 | -200.949 | 1.000 |

#### Final selection

With the variables selected above, we generated candidate models of their possible interactions. We ranked them using AICc.

Code

```
PD_models <- list(
PD_m1  <- glmmTMB(n~ Rep,                      family =binomial, data = PELE_D),
PD_m2  <- glmmTMB(n~ Rep+ Sex,                 family =binomial, data = PELE_D),
PD_m3  <- glmmTMB(n~ Rep+ Wght,                family =binomial, data = PELE_D),
PD_m4  <- glmmTMB(n~ Rep+ Wght+I(Wght^2),      family =binomial, data = PELE_D),
PD_m5  <- glmmTMB(n~ Rep+ Avg_VH,              family =binomial, data = PELE_D),
PD_m6  <- glmmTMB(n~ Rep+ Avg_VH+I(Avg_VH^2),  family =binomial, data = PELE_D),
PD_m7  <- glmmTMB(n~ Rep+ Avg_LC,              family =binomial, data = PELE_D),
PD_m8  <- glmmTMB(n~ Rep+ Temp+I(Temp^2),      family =binomial, data = PELE_D),
PD_m9  <- glmmTMB(n~ Rep* Sex,                 family =binomial, data = PELE_D),
PD_m10  <- glmmTMB(n~ Rep* Wght,                family =binomial, data = PELE_D),
PD_m11  <- glmmTMB(n~ Rep* (Wght+I(Wght^2)),    family =binomial, data = PELE_D),
PD_m12  <- glmmTMB(n~ Rep* Avg_VH,              family =binomial, data = PELE_D),
PD_m13 <- glmmTMB(n~ Rep* (Avg_VH+I(Avg_VH^2)),family =binomial, data = PELE_D),
PD_m14  <- glmmTMB(n~ Rep* Avg_LC,              family =binomial, data = PELE_D),
PD_m15 <- glmmTMB(n~ Rep* (Temp+I(Temp^2)),    family =binomial, data = PELE_D),

PD_m16  <- glmmTMB(n~ Sex,                      family =binomial, data = PELE_D),
PD_m17  <- glmmTMB(n~ Sex+ Wght,                family =binomial, data = PELE_D),
PD_m18 <- glmmTMB(n~ Sex+ Wght+I(Wght^2),      family =binomial, data = PELE_D),
PD_m19  <- glmmTMB(n~ Sex+ Avg_VH,              family =binomial, data = PELE_D),
PD_m20  <- glmmTMB(n~ Sex+ Avg_VH+I(Avg_VH^2),  family =binomial, data = PELE_D),
PD_m21 <- glmmTMB(n~ Sex+ Avg_LC,              family =binomial, data = PELE_D),
PD_m22 <- glmmTMB(n~ Sex+ Temp+I(Temp^2),      family =binomial, data = PELE_D),
PD_m23 <- glmmTMB(n~ Sex* Wght,                family =binomial, data = PELE_D),
PD_m24 <- glmmTMB(n~ Sex* (Wght+I(Wght^2)),    family =binomial, data = PELE_D),
PD_m25 <- glmmTMB(n~ Sex* Avg_VH,              family =binomial, data = PELE_D),
PD_m26 <- glmmTMB(n~ Sex* (Avg_VH+I(Avg_VH^2)),family =binomial, data = PELE_D),
PD_m27 <- glmmTMB(n~ Sex* Avg_LC,              family =binomial, data = PELE_D),
PD_m28 <- glmmTMB(n~ Sex* (Temp+I(Temp^2)),    family =binomial, data = PELE_D),


PD_m29 <- glmmTMB(n~ Wght,                        family =binomial, data = PELE_D),
PD_m30 <- glmmTMB(n~ Wght+ Avg_VH,                family =binomial, data = PELE_D),
PD_m31 <- glmmTMB(n~ Wght+ Avg_VH+I(Avg_VH^2),    family =binomial, data = PELE_D),
PD_m32 <- glmmTMB(n~ Wght+ Avg_LC,                family =binomial, data = PELE_D),
PD_m33 <- glmmTMB(n~ Wght+ Temp+I(Temp^2),        family =binomial, data = PELE_D),
PD_m34 <- glmmTMB(n~ Wght* Avg_VH,                family =binomial, data = PELE_D),
PD_m35 <- glmmTMB(n~ Wght* Avg_LC,                family =binomial, data = PELE_D),
PD_m36 <- glmmTMB(n~ Wght* (Temp+I(Temp^2)),      family =binomial, data = PELE_D),

PD_m37 <- glmmTMB(n~ Wght+I(Wght^2),              family =binomial, data = PELE_D),

PD_m38 <- glmmTMB(n~ Wght+I(Wght^2)+ Avg_VH,      family =binomial, data = PELE_D),
PD_m39 <- glmmTMB(n~ Wght+I(Wght^2)+ Avg_VH+I(Avg_VH^2),family =binomial, data = PELE_D),
PD_m40 <- glmmTMB(n~ Wght+I(Wght^2)+ Avg_LC,      family =binomial, data = PELE_D),
PD_m41 <- glmmTMB(n~ Wght+I(Wght^2)+ Temp+I(Temp^2), family =binomial, data = PELE_D),
PD_m42 <- glmmTMB(n~ (Wght+I(Wght^2))* Avg_VH,    family =binomial, data = PELE_D),
PD_m43 <- glmmTMB(n~ Wght+I(Wght^2)* Avg_LC,      family =binomial, data = PELE_D),

PD_m44 <- glmmTMB(n~ Avg_VH,                      family =binomial, data = PELE_D),
PD_m45 <- glmmTMB(n~ Avg_VH+ Avg_LC,              family =binomial, data = PELE_D),
PD_m46 <- glmmTMB(n~ Avg_VH+ Temp+I(Temp^2),      family =binomial, data = PELE_D),
PD_m47 <- glmmTMB(n~ Avg_VH* Avg_LC,              family =binomial, data = PELE_D),
PD_m48 <- glmmTMB(n~ Avg_VH* (Temp+I(Temp^2)),    family =binomial, data = PELE_D),

PD_m49 <- glmmTMB(n~ Avg_VH+ I(Avg_VH^2),         family =binomial, data = PELE_D),
PD_m50 <- glmmTMB(n~ Avg_VH+ I(Avg_VH^2)+Avg_LC,  family =binomial, data = PELE_D),
PD_m51 <- glmmTMB(n~ Avg_VH+ I(Avg_VH^2)+ Temp+I(Temp^2),family =binomial, data = PELE_D),

PD_m52 <- glmmTMB(n~ Avg_LC,                      family =binomial, data = PELE_D),
PD_m53 <- glmmTMB(n~ Avg_LC+ Temp+I(Temp^2),      family =binomial, data = PELE_D),
PD_m54 <- glmmTMB(n~ Avg_LC* Temp+I(Temp^2),      family =binomial, data = PELE_D),

PD_m55 <- glmmTMB(n~ Temp+I(Temp^2),              family =binomial, data = PELE_D),

PD_m56 <- glmmTMB(n~ 1,                           family =binomial, data = PELE_D),

PD_m57 <- glmmTMB(n~ Month,                       family =binomial, data = PELE_D),
PD_m58 <- glmmTMB(n~ Month *Rep,                  family =binomial, data = PELE_D),
PD_m59 <- glmmTMB(n~ Month* Sex,                  family =binomial, data = PELE_D),
PD_m60 <- glmmTMB(n~ Month* Wght,                 family =binomial, data = PELE_D),
PD_m61 <- glmmTMB(n~ Month* (Wght+I(Wght^2)),     family =binomial, data = PELE_D),
PD_m62 <- glmmTMB(n~ Month* Avg_LC,               family =binomial, data = PELE_D),
PD_m63 <- glmmTMB(n~ Month* (Temp+I(Temp^2)),     family =binomial, data = PELE_D)
)

PD_sel <- aictab(PD_models, sort = F)

PD_sel_table <- get_table_models(PD_models, PD_sel, "Prevalence")

  kbl(PD_sel_table,
    caption = "Final model selection to identify drivers influencing tick presence in Peromyscus leucopus",
    digits = 2) %>% 
  kable_styling(bootstrap_options = c("striped", "hover"), full_width = F)
```

Final model selection to identify drivers influencing tick presence in Peromyscus leucopus

| Formula | Modnames | K | AICc | Delta\_AICc | ModelLik | AICcWt | LL |
| --- | --- | --- | --- | --- | --- | --- | --- |
| Tick Prevalence ~ Month | Mod57 | 7 | 357.24 | 0.00 | 1.00 | 0.58 | -171.45 |
| Tick Prevalence ~ Month \* Rep | Mod58 | 14 | 359.72 | 2.48 | 0.29 | 0.17 | -165.19 |
| Tick Prevalence ~ Month \* Sex | Mod59 | 14 | 360.61 | 3.37 | 0.19 | 0.11 | -165.64 |
| Tick Prevalence ~ Month \* Avg\_LC | Mod62 | 14 | 361.33 | 4.09 | 0.13 | 0.08 | -166.00 |
| Tick Prevalence ~ Month \* Wght | Mod60 | 14 | 362.14 | 4.90 | 0.09 | 0.05 | -166.41 |
| Tick Prevalence ~ Month \* (Wght + I(Wght^2)) | Mod61 | 21 | 363.90 | 6.66 | 0.04 | 0.02 | -159.45 |
| Tick Prevalence ~ Month \* (Temp + I(Temp^2)) | Mod63 | 21 | 373.24 | 16.00 | 0.00 | 0.00 | -164.12 |
| Tick Prevalence ~ Avg\_LC + Temp + I(Temp^2) | Mod53 | 4 | 380.87 | 23.63 | 0.00 | 0.00 | -186.37 |
| Tick Prevalence ~ Avg\_VH + I(Avg\_VH^2) + Temp + I(Temp^2) | Mod51 | 5 | 381.71 | 24.47 | 0.00 | 0.00 | -185.76 |
| Tick Prevalence ~ Avg\_VH \* (Temp + I(Temp^2)) | Mod48 | 6 | 381.79 | 24.55 | 0.00 | 0.00 | -184.77 |
| Tick Prevalence ~ Avg\_LC \* Temp + I(Temp^2) | Mod54 | 5 | 382.33 | 25.09 | 0.00 | 0.00 | -186.07 |
| Tick Prevalence ~ Avg\_VH + Temp + I(Temp^2) | Mod46 | 4 | 383.50 | 26.26 | 0.00 | 0.00 | -187.69 |
| Tick Prevalence ~ Wght + I(Wght^2) + Temp + I(Temp^2) | Mod41 | 5 | 385.01 | 27.77 | 0.00 | 0.00 | -187.41 |
| Tick Prevalence ~ Rep \* (Temp + I(Temp^2)) | Mod15 | 6 | 385.15 | 27.91 | 0.00 | 0.00 | -186.45 |
| Tick Prevalence ~ Rep + Temp + I(Temp^2) | Mod8 | 4 | 386.91 | 29.67 | 0.00 | 0.00 | -189.39 |
| Tick Prevalence ~ Temp + I(Temp^2) | Mod55 | 3 | 388.36 | 31.12 | 0.00 | 0.00 | -191.15 |
| Tick Prevalence ~ Sex + Temp + I(Temp^2) | Mod22 | 4 | 388.65 | 31.41 | 0.00 | 0.00 | -190.26 |
| Tick Prevalence ~ Sex \* (Temp + I(Temp^2)) | Mod28 | 6 | 389.11 | 31.87 | 0.00 | 0.00 | -188.42 |
| Tick Prevalence ~ Wght + Temp + I(Temp^2) | Mod33 | 4 | 390.11 | 32.87 | 0.00 | 0.00 | -190.99 |
| Tick Prevalence ~ Wght \* (Temp + I(Temp^2)) | Mod36 | 6 | 393.05 | 35.81 | 0.00 | 0.00 | -190.40 |
| Tick Prevalence ~ Rep + Avg\_VH + I(Avg\_VH^2) | Mod6 | 4 | 400.64 | 43.40 | 0.00 | 0.00 | -196.26 |
| Tick Prevalence ~ Rep + Avg\_VH | Mod5 | 3 | 402.01 | 44.77 | 0.00 | 0.00 | -197.97 |
| Tick Prevalence ~ Rep \* Avg\_LC | Mod14 | 4 | 402.36 | 45.12 | 0.00 | 0.00 | -197.12 |
| Tick Prevalence ~ Rep + Avg\_LC | Mod7 | 3 | 402.84 | 45.60 | 0.00 | 0.00 | -198.39 |
| Tick Prevalence ~ Rep \* Avg\_VH | Mod12 | 4 | 403.65 | 46.41 | 0.00 | 0.00 | -197.76 |
| Tick Prevalence ~ Rep \* (Avg\_VH + I(Avg\_VH^2)) | Mod13 | 6 | 404.02 | 46.78 | 0.00 | 0.00 | -195.88 |
| Tick Prevalence ~ Wght + I(Wght^2) + Avg\_VH | Mod38 | 4 | 404.52 | 47.28 | 0.00 | 0.00 | -198.20 |
| Tick Prevalence ~ Wght \* Avg\_LC | Mod35 | 4 | 404.59 | 47.35 | 0.00 | 0.00 | -198.23 |
| Tick Prevalence ~ Sex + Avg\_VH + I(Avg\_VH^2) | Mod20 | 4 | 404.61 | 47.37 | 0.00 | 0.00 | -198.25 |
| Tick Prevalence ~ Rep \* Wght | Mod10 | 4 | 404.79 | 47.55 | 0.00 | 0.00 | -198.33 |
| Tick Prevalence ~ Wght + I(Wght^2) \* Avg\_LC | Mod43 | 5 | 404.81 | 47.57 | 0.00 | 0.00 | -197.31 |
| Tick Prevalence ~ Wght + I(Wght^2) + Avg\_VH + I(Avg\_VH^2) | Mod39 | 5 | 404.87 | 47.63 | 0.00 | 0.00 | -197.35 |
| Tick Prevalence ~ Avg\_VH + I(Avg\_VH^2) | Mod49 | 3 | 405.02 | 47.78 | 0.00 | 0.00 | -199.47 |
| Tick Prevalence ~ Avg\_VH | Mod44 | 2 | 405.36 | 48.12 | 0.00 | 0.00 | -200.66 |
| Tick Prevalence ~ Sex + Avg\_VH | Mod19 | 3 | 405.42 | 48.18 | 0.00 | 0.00 | -199.68 |
| Tick Prevalence ~ Sex \* (Wght + I(Wght^2)) | Mod24 | 6 | 405.81 | 48.57 | 0.00 | 0.00 | -196.78 |
| Tick Prevalence ~ Sex + Avg\_LC | Mod21 | 3 | 405.81 | 48.57 | 0.00 | 0.00 | -199.87 |
| Tick Prevalence ~ Avg\_LC | Mod52 | 2 | 405.84 | 48.60 | 0.00 | 0.00 | -200.90 |
| Tick Prevalence ~ Wght + I(Wght^2) + Avg\_LC | Mod40 | 4 | 405.84 | 48.60 | 0.00 | 0.00 | -198.86 |
| Tick Prevalence ~ Sex \* Wght | Mod23 | 4 | 405.94 | 48.70 | 0.00 | 0.00 | -198.91 |
| Tick Prevalence ~ Rep + Wght | Mod3 | 3 | 406.30 | 49.06 | 0.00 | 0.00 | -200.12 |
| Tick Prevalence ~ Rep + Wght + I(Wght^2) | Mod4 | 4 | 406.36 | 49.12 | 0.00 | 0.00 | -199.12 |
| Tick Prevalence ~ Wght + Avg\_VH + I(Avg\_VH^2) | Mod31 | 4 | 406.58 | 49.34 | 0.00 | 0.00 | -199.23 |
| Tick Prevalence ~ Avg\_VH \* Avg\_LC | Mod47 | 4 | 406.61 | 49.37 | 0.00 | 0.00 | -199.24 |
| Tick Prevalence ~ Wght + Avg\_VH | Mod30 | 3 | 406.69 | 49.45 | 0.00 | 0.00 | -200.31 |
| Tick Prevalence ~ Avg\_VH + Avg\_LC | Mod45 | 3 | 406.71 | 49.47 | 0.00 | 0.00 | -200.32 |
| Tick Prevalence ~ Avg\_VH + I(Avg\_VH^2) + Avg\_LC | Mod50 | 4 | 407.03 | 49.79 | 0.00 | 0.00 | -199.45 |
| Tick Prevalence ~ Wght + Avg\_LC | Mod32 | 3 | 407.03 | 49.79 | 0.00 | 0.00 | -200.48 |
| Tick Prevalence ~ Sex \* Avg\_VH | Mod25 | 4 | 407.06 | 49.82 | 0.00 | 0.00 | -199.47 |
| Tick Prevalence ~ (Wght + I(Wght^2)) \* Avg\_VH | Mod42 | 6 | 407.45 | 50.21 | 0.00 | 0.00 | -197.59 |
| Tick Prevalence ~ Sex \* Avg\_LC | Mod27 | 4 | 407.68 | 50.44 | 0.00 | 0.00 | -199.78 |
| Tick Prevalence ~ Wght + I(Wght^2) | Mod37 | 3 | 407.75 | 50.51 | 0.00 | 0.00 | -200.84 |
| Tick Prevalence ~ Sex \* (Avg\_VH + I(Avg\_VH^2)) | Mod26 | 6 | 408.01 | 50.77 | 0.00 | 0.00 | -197.88 |
| Tick Prevalence ~ Sex + Wght + I(Wght^2) | Mod18 | 4 | 408.16 | 50.92 | 0.00 | 0.00 | -200.02 |
| Tick Prevalence ~ Wght \* Avg\_VH | Mod34 | 4 | 408.42 | 51.18 | 0.00 | 0.00 | -200.15 |
| Tick Prevalence ~ Rep \* (Wght + I(Wght^2)) | Mod11 | 6 | 408.44 | 51.20 | 0.00 | 0.00 | -198.09 |
| Tick Prevalence ~ Rep \* Sex | Mod9 | 4 | 408.50 | 51.26 | 0.00 | 0.00 | -200.19 |
| Tick Prevalence ~ Rep | Mod1 | 2 | 408.72 | 51.48 | 0.00 | 0.00 | -202.34 |
| Tick Prevalence ~ Sex | Mod16 | 2 | 409.12 | 51.88 | 0.00 | 0.00 | -202.54 |
| Tick Prevalence ~ Wght | Mod29 | 2 | 409.19 | 51.95 | 0.00 | 0.00 | -202.58 |
| Tick Prevalence ~ 1 | Mod56 | 1 | 409.24 | 52.00 | 0.00 | 0.00 | -203.62 |
| Tick Prevalence ~ Sex + Wght | Mod17 | 3 | 409.37 | 52.13 | 0.00 | 0.00 | -201.65 |
| Tick Prevalence ~ Rep + Sex | Mod2 | 3 | 409.62 | 52.38 | 0.00 | 0.00 | -201.77 |

We inspected the 85% confidence intervals of the regression coefficients of the selected models.

Code

```
PD_final_best <- list(PD_m57=PD_m57)

PD_final_best_ci <- map2_df(names(PD_final_best), PD_final_best, get_ci)

 kbl(PD_final_best_ci,
    caption = "Selected models confidence interval table",
    digits = 2) %>% 
  kable_styling(bootstrap_options = c("striped", "hover"), full_width = F)
```

Selected models confidence interval table

| Parameter | Coefficient | CI\_low | CI\_high | Model | Informative |
| --- | --- | --- | --- | --- | --- |
| Month3 | -0.38 | -1.11 | 0.35 | PD\_m57 | no |
| Month5 | -1.27 | -2.03 | -0.51 | PD\_m57 | yes |
| Month9 | -3.89 | -5.42 | -2.35 | PD\_m57 | yes |
| Month10 | -2.25 | -2.96 | -1.55 | PD\_m57 | yes |
| Month11 | -0.24 | -0.81 | 0.34 | PD\_m57 | no |
| Month12 | -0.87 | -1.69 | -0.04 | PD\_m57 | yes |

Code

```
ci_plot(PD_final_best_ci)+ labs(title= "Peromyscus leucopus models 85%CI")
```

#### Selcted models

Subsequently, we inspect the residuals of the models whose confidence intervals do not overlap 0.

Code

```
PD_res <- simulateResiduals(PD_m57, plot=T)
```

We build the summary table of the selected models

Code

```
PD_1_sum <- model_parameters(PD_m57, digits=2, ci= 0.85) %>% 
  mutate(Family= "Bernoulli",
         Model= "Model 57") %>% 
  select(-SE, -z, -df_error)


kbl(PD_1_sum,
    caption = "P. leocopus - Dermacentor",
    digits = 3) %>% 
  kable_styling(bootstrap_options = c("striped", "hover"), full_width = F)
```

P. leocopus - Dermacentor

| Parameter | Coefficient | CI | CI\_low | CI\_high | p | Effects | Family | Model |
| --- | --- | --- | --- | --- | --- | --- | --- | --- |
| (Intercept) | 0.172 | 0.85 | -0.317 | 0.660 | 0.613 | fixed | Bernoulli | Model 57 |
| Month3 | -0.379 | 0.85 | -1.106 | 0.347 | 0.452 | fixed | Bernoulli | Model 57 |
| Month5 | -1.270 | 0.85 | -2.035 | -0.506 | 0.017 | fixed | Bernoulli | Model 57 |
| Month9 | -3.885 | 0.85 | -5.422 | -2.349 | 0.000 | fixed | Bernoulli | Model 57 |
| Month10 | -2.251 | 0.85 | -2.957 | -1.546 | 0.000 | fixed | Bernoulli | Model 57 |
| Month11 | -0.238 | 0.85 | -0.812 | 0.336 | 0.551 | fixed | Bernoulli | Model 57 |
| Month12 | -0.865 | 0.85 | -1.691 | -0.039 | 0.132 | fixed | Bernoulli | Model 57 |

#### Prediction plots

Code

```
PD_Pred1 <- ggeffect(PD_m57, terms = c("Month"), ci_level = 0.85)

(PD_pred1_plot <- catpred2_plot(PD_Pred1, title= NULL, 
                               "Month", 
                               PELE_D, cat=Month, 
                               col = "deepskyblue2")+
  labs(y="Tick presence probability" ))
```

## ***Sigmodon hispidus***

### ***Dermacentor*** larvae

Code

```
SIHI_D <- H_data %>% 
  filter(SM_Sp == "SIHI" & 
           Tick_gen %in% c("Dermacentor", "No tick") & 
           Tick_Age %in% c("Larvae", "No tick") )
```

#### Random effects

To evaluate the random effects structure, models with all fixed variables and possible random structures were ranked: transect ID random intercept and a model with no random effects. Models were ranked using the Akaike information criterion corrected for small samples (AICc).

Code

```
SD_random <- random_eff(SIHI_D, binomial)
kbl(compare_performance(SD_random, metrics = "AICc"),
    digits = 3) %>% 
  kable_styling(bootstrap_options = c("striped", "hover"), full_width = F)
```

| Name | Model | AICc | AICc\_wt |
| --- | --- | --- | --- |
| Transect | glmmTMB | 176.585 | 0.259 |
| norandom | glmmTMB | 174.478 | 0.741 |

Subsequently, the residuals of the selected model are inspected.

Code

```
SD_norandom_res <- simulateResiduals(SD_random$norandom, plot = T)
```

#### Evaluate any spatial correlation issues

Code

```
SD_audf <- get_sau_data(SD_norandom_res, SIHI_D)
SD_groups <- split(SD_audf, SD_audf$survey)
SD_corelog <- map(SD_groups, get_correlog) %>% compact()
SD_corelogbase <- map(SD_corelog, get_correlog_base)
SD_corelogplot <- map(SD_corelogbase, get_correlogplot)
SD_corelogplot
```

```
500  of  5000 
1000  of  5000 
1500  of  5000 
2000  of  5000 
2500  of  5000 
3000  of  5000 
3500  of  5000 
4000  of  5000 
4500  of  5000 
5000  of  5000 
500  of  5000 
1000  of  5000 
1500  of  5000 
2000  of  5000 
2500  of  5000 
3000  of  5000 
3500  of  5000 
4000  of  5000 
4500  of  5000 
5000  of  5000 
500  of  5000 
1000  of  5000 
1500  of  5000 
2000  of  5000 
2500  of  5000 
3000  of  5000 
3500  of  5000 
4000  of  5000 
4500  of  5000 
5000  of  5000 
500  of  5000 
1000  of  5000 
1500  of  5000 
2000  of  5000 
2500  of  5000 
3000  of  5000 
3500  of  5000 
4000  of  5000 
4500  of  5000 
5000  of  5000 
500  of  5000 
1000  of  5000 
1500  of  5000 
2000  of  5000 
2500  of  5000 
3000  of  5000 
3500  of  5000 
4000  of  5000 
4500  of  5000 
5000  of  5000
```

```
$Fall_2019
```

-“Fall 2019 correlogram” -“Fall 2020 correlogram” -“Spring 2020 correlogram” -“Winter 2019 correlogram” -“Winter 2020 correlogram”

```
$Fall_2020
```

-“Fall 2019 correlogram” -“Fall 2020 correlogram” -“Spring 2020 correlogram” -“Winter 2019 correlogram” -“Winter 2020 correlogram”

```
$Spring_2020
```

-“Fall 2019 correlogram” -“Fall 2020 correlogram” -“Spring 2020 correlogram” -“Winter 2019 correlogram” -“Winter 2020 correlogram”

```
$Winter_2019
```

-“Fall 2019 correlogram” -“Fall 2020 correlogram” -“Spring 2020 correlogram” -“Winter 2019 correlogram” -“Winter 2020 correlogram”

```
$Winter_2020
```

-“Fall 2019 correlogram” -“Fall 2020 correlogram” -“Spring 2020 correlogram” -“Winter 2019 correlogram” -“Winter 2020 correlogram”

According to the inspection of the residuals, the selected model showed adequate goodness-of-fit or spatial autocorrelation problems.

#### Host variables

Univariate models were created for the selection of host variables. The models were ranked using AICc. Variables from models with Delta <2 were used in the final model selection.

Code

```
SD_H <-  get_H_models(SIHI_D, family = binomial(), zi= FALSE)

SD_H_sel <-aictab(SD_H, sort = T)

kbl(SD_H_sel,
    caption = "Model selection table for host variables",
    digits = 3) %>% 
  kable_styling(bootstrap_options = c("striped", "hover"), full_width = F)
```

Model selection table for host variables

|  | Modnames | K | AICc | Delta\_AICc | ModelLik | AICcWt | LL | Cum.Wt |
| --- | --- | --- | --- | --- | --- | --- | --- | --- |
| 4 | Rep | 2 | 174.808 | 0.000 | 1.000 | 0.709 | -85.392 | 0.709 |
| 3 | Sex | 2 | 177.969 | 3.161 | 0.206 | 0.146 | -86.973 | 0.855 |
| 1 | Weight | 2 | 178.670 | 3.862 | 0.145 | 0.103 | -87.323 | 0.958 |
| 2 | Weight2 | 3 | 180.437 | 5.629 | 0.060 | 0.042 | -87.195 | 1.000 |

#### Habitat variables

Univariate models were created for the selection of habitat variables. The models were ranked using AICc. Variables from models with Delta <2 were used in the final model selection.

Code

```
SD_Ha <- get_Hamodels(SIHI_D, family = binomial, zi= FALSE)
SD_Ha_sel <- aictab(SD_Ha, sort = T)
  kbl(SD_Ha_sel,
    caption = "Model selection table for habitat variables",
    digits = 3) %>% 
  kable_styling(bootstrap_options = c("striped", "hover"), full_width = F)
```

Model selection table for habitat variables

|  | Modnames | K | AICc | Delta\_AICc | ModelLik | AICcWt | LL | Cum.Wt |
| --- | --- | --- | --- | --- | --- | --- | --- | --- |
| 5 | Vegetation\_height2 | 3 | 162.955 | 0.000 | 1.000 | 0.973 | -78.454 | 0.973 |
| 3 | Canopy\_Cover | 2 | 170.539 | 7.583 | 0.023 | 0.022 | -83.257 | 0.995 |
| 6 | Litter\_Cover | 2 | 174.661 | 11.706 | 0.003 | 0.003 | -85.319 | 0.998 |
| 4 | Vegetation\_height | 2 | 175.697 | 12.742 | 0.002 | 0.002 | -85.837 | 0.999 |
| 1 | Litter\_depth | 2 | 178.367 | 15.411 | 0.000 | 0.000 | -87.172 | 1.000 |
| 2 | Herbaceus\_Cover | 2 | 178.562 | 15.606 | 0.000 | 0.000 | -87.269 | 1.000 |

#### Weather variables

Univariate models were created for the selection of weather variables. The models were ranked using AICc. Variables from models with Delta <2 were used in the final model selection.

Code

```
SD_W <- get_Wmodels(SIHI_D, family = binomial, zi= FALSE)
SD_W_sel <-aictab(SD_W, sort = T)
  kbl(SD_W_sel,
    caption = "Model selection table for  weather variables",
    digits = 3) %>% 
  kable_styling(bootstrap_options = c("striped", "hover"), full_width = F)
```

Model selection table for weather variables

|  | Modnames | K | AICc | Delta\_AICc | ModelLik | AICcWt | LL | Cum.Wt |
| --- | --- | --- | --- | --- | --- | --- | --- | --- |
| 4 | Season | 3 | 174.971 | 0.000 | 1.000 | 0.607 | -84.462 | 0.607 |
| 2 | Temp2 | 3 | 177.653 | 2.682 | 0.262 | 0.159 | -85.803 | 0.765 |
| 1 | Temp | 2 | 178.156 | 3.185 | 0.203 | 0.123 | -87.066 | 0.889 |
| 3 | Rain | 2 | 178.360 | 3.389 | 0.184 | 0.111 | -87.168 | 1.000 |

#### Final selection

With the variables selected above, we generated candidate models of their possible interactions. We ranked them using AICc.

Code

```
SD_models <- list(

SD_m1 <- glmmTMB(n~ Rep,                          family =binomial, data = SIHI_D),
SD_m2 <- glmmTMB(n~ Rep+ Avg_VH+I(Avg_VH^2),      family =binomial, data = SIHI_D),
SD_m3 <- glmmTMB(n~ Rep+ Season,                  family =binomial, data = SIHI_D),
SD_m4 <- glmmTMB(n~ Rep* Season,                  family =binomial, data = SIHI_D),

SD_m5 <- glmmTMB(n~ Avg_VH+I(Avg_VH^2),          family =binomial, data = SIHI_D),
SD_m6 <- glmmTMB(n~ Avg_VH+I(Avg_VH^2)+ Season,  family =binomial, data = SIHI_D),
SD_m7 <- glmmTMB(n~ Avg_VH+I(Avg_VH^2)* Season,  family =binomial, data = SIHI_D),

SD_m8 <- glmmTMB(n~ Season,                      family =binomial, data = SIHI_D),

SD_m9 <- glmmTMB(n~ 1,                           family =binomial, data = SIHI_D),

SD_m10 <- glmmTMB(n~ Month,                       family =binomial, data = SIHI_D),
SD_m11 <- glmmTMB(n~ Month+Rep,                   family =binomial, data = SIHI_D),
SD_m12 <- glmmTMB(n~ Month*Rep,                   family =binomial, data = SIHI_D)


)


SD_sel <- aictab(SD_models, sort = F)
SD_sel_table <- get_table_models(SD_models, SD_sel, "Prevalence")


  kbl(SD_sel_table,
    caption = "Final model selection to identify drivers influencing tick presence in Sigmodon hispidus",
    digits = 2) %>% 
  kable_styling(bootstrap_options = c("striped", "hover"), full_width = F)
```

Final model selection to identify drivers influencing tick presence in Sigmodon hispidus

| Formula | Modnames | K | AICc | Delta\_AICc | ModelLik | AICcWt | LL |
| --- | --- | --- | --- | --- | --- | --- | --- |
| Tick Prevalence ~ Rep + Avg\_VH + I(Avg\_VH^2) | Mod2 | 4 | 160.51 | 0.00 | 1.00 | 0.40 | -76.21 |
| Tick Prevalence ~ Avg\_VH + I(Avg\_VH^2) \* Season | Mod7 | 7 | 162.08 | 1.57 | 0.46 | 0.18 | -73.93 |
| Tick Prevalence ~ Avg\_VH + I(Avg\_VH^2) + Season | Mod6 | 5 | 162.23 | 1.72 | 0.42 | 0.17 | -76.05 |
| Tick Prevalence ~ Avg\_VH + I(Avg\_VH^2) | Mod5 | 3 | 162.96 | 2.45 | 0.29 | 0.12 | -78.45 |
| Tick Prevalence ~ Month + Rep | Mod11 | 8 | 163.24 | 2.73 | 0.26 | 0.10 | -73.47 |
| Tick Prevalence ~ Month | Mod10 | 7 | 166.52 | 6.01 | 0.05 | 0.02 | -76.15 |
| Tick Prevalence ~ Month \* Rep | Mod12 | 13 | 171.87 | 11.36 | 0.00 | 0.00 | -72.56 |
| Tick Prevalence ~ Rep + Season | Mod3 | 4 | 172.83 | 12.32 | 0.00 | 0.00 | -82.38 |
| Tick Prevalence ~ Rep | Mod1 | 2 | 174.81 | 14.30 | 0.00 | 0.00 | -85.39 |
| Tick Prevalence ~ Season | Mod8 | 3 | 174.97 | 14.46 | 0.00 | 0.00 | -84.46 |
| Tick Prevalence ~ Rep \* Season | Mod4 | 6 | 176.20 | 15.69 | 0.00 | 0.00 | -82.02 |
| Tick Prevalence ~ 1 | Mod9 | 1 | 176.77 | 16.26 | 0.00 | 0.00 | -87.38 |

We inspected the 85% confidence intervals of the regression coefficients of the selected models.

Code

```
SD_final_best <- list(SD_m2=SD_m2, SD_m7=SD_m7, SD_m6=SD_m6)

SD_final_best_ci <- map2_df(names(SD_final_best), SD_final_best, get_ci)

 kbl(SD_final_best_ci,
    caption = "Selected models confidence interval table",
    digits = 2) %>% 
  kable_styling(bootstrap_options = c("striped", "hover"), full_width = F)
```

Selected models confidence interval table

| Parameter | Coefficient | CI\_low | CI\_high | Model | Informative |
| --- | --- | --- | --- | --- | --- |
| RepY | -1.73 | -3.24 | -0.23 | SD\_m2 | yes |
| Avg\_VH | -0.05 | -0.07 | -0.03 | SD\_m2 | yes |
| I(Avg\_VH^2) | 0.00 | 0.00 | 0.00 | SD\_m2 | yes |
| Avg\_VH | -0.33 | -0.50 | -0.15 | SD\_m7 | yes |
| I(Avg\_VH^2) | 0.00 | 0.00 | 0.00 | SD\_m7 | yes |
| SeasonSpring | -29.27 | -48.90 | -9.64 | SD\_m7 | yes |
| SeasonWinter | -22.82 | -36.90 | -8.74 | SD\_m7 | yes |
| I(Avg\_VH^2):SeasonSpring | 0.21 | -0.18 | 0.60 | SD\_m7 | no |
| I(Avg\_VH^2):SeasonWinter | 0.00 | 0.00 | 0.00 | SD\_m7 | no |
| Avg\_VH | -0.26 | -0.43 | -0.09 | SD\_m6 | yes |
| I(Avg\_VH^2) | 0.00 | 0.00 | 0.00 | SD\_m6 | yes |
| SeasonSpring | -17.55 | -31.19 | -3.91 | SD\_m6 | yes |
| SeasonWinter | -17.43 | -30.99 | -3.87 | SD\_m6 | yes |

Code

```
ci_plot(SD_final_best_ci)+ labs(title= "Sigmodon hispidus models 85%CI")
```

All models had fairly wide confidence intervals O were not possible to estimate in the SD\_m24 model. In all cases it was not possible to obtain a reliable estimate of the regression coefficients.

#### Selected models

Subsequently, we inspect the residuals of the models whose confidence intervals do not overlap 0.

Code

```
SD_res <- lapply(list(SD_m2, SD_m7), simulateResiduals, plot= TRUE)
```

Residual inspection of model Tick Prevalence ~ Rep + Avg\_VH + I(Avg\_VH^2)

Residual inspection of model Tick Prevalence ~ Avg\_VH + I(Avg\_VH^2) \* Season

Both models present variance homogeneity problems. Since the bernoulli distribution has fixed dispersion, it is not possible to model this variation. However, the residual plot shows that the deviation from the quartile is not so severe.

#### Prediction plots

Code

```
SD_Pred1 <- ggeffect(SD_m2, terms = c("Avg_VH", "Rep"), ci_level = 0.85)

SD_Pred2 <- ggeffect(SD_m7, terms = c("Avg_VH","Season"), ci_level = 0.85)


SD_pred1_plot <- ggplot()+
  geom_ribbon(data= SD_Pred1, aes(x= x, y=predicted,
                                  ymin=conf.low, ymax=conf.high,
                                  fill=group), alpha= 0.4)+
   geom_line(data= SD_Pred1, aes(x= x, y= predicted, col= group),
            linewidth= 1)+
  labs(x= "Average vegetation height", y= "Tick presence probability",
       fill= "Reproductive activity",
       col= "Reproductive activity")+
    guides(col= guide_legend(position = "inside"),
         fill= guide_legend(position = "inside"))+
    scale_color_viridis_d()+
    scale_fill_viridis_d()+
    theme_bw(base_size = 12)+
    theme(legend.position.inside = c(0.7, 0.8),
          legend.background = element_blank())

SD_pred2_plot <- ggplot()+
  geom_ribbon(data= SD_Pred2, aes(x= x, y=predicted,
                                  ymin=conf.low, ymax=conf.high,
                                  fill=group), alpha= 0.4)+
   geom_line(data= SD_Pred2, aes(x= x, y= predicted, col= group),
            linewidth= 1)+
  labs(x= "Average vegetation height", y= "Tick presence probability",
       fill= "Month",
       col= "Month")+
    guides(col= guide_legend(position = "inside"),
         fill= guide_legend(position = "inside"))+
    scale_color_viridis_d()+
    scale_fill_viridis_d()+
    theme_bw(base_size = 12)+
    theme(legend.position.inside = c(0.7, 0.8),
          legend.background = element_blank())

SD_pred1_plot
SD_pred2_plot
```

-“Prediction plot of model Tick Prevalence ~ Rep + Avg\_VH + I(Avg\_VH^2)” -“Prediction plot of model Tick Prevalence ~ Avg\_VH + I(Avg\_VH^2) \* Season”

-“Prediction plot of model Tick Prevalence ~ Rep + Avg\_VH + I(Avg\_VH^2)” -“Prediction plot of model Tick Prevalence ~ Avg\_VH + I(Avg\_VH^2) \* Season”

# Final plots

Code

```
(predplots <- (OD_pred1_plot+ OD_pred2_plot +PD_pred1_plot)+
   plot_layout(ncol = 2, axes = "collect")+
   plot_annotation(tag_levels = 'A'))
```

Code

```
ggsave(plot = predplots, filename = "Figs/Prevalence_predplots.png",
       width=9, height=8.5)

ggsave(plot = predplots, filename = "Figs/Prevalence_predplots.svg",
       width=9, height=8.5)
```


##### Source Code

```
---
title: "Statistical modeling process for the analysis of the prevalence of Dermacentor larvae in two species of small rodents."
format:
  html:
   message: false
   warning: false
   code-fold: true
   code-tools: true
   toc: true
   toc-depth: 3
   self-contained: true
   theme: journal
---

# Packages

List of packages used

```{r}
library(tidyverse)
library(glmmTMB)
library(performance)
library(modelbased)
library(DHARMa)
library(patchwork)
library(parameters)
library(AICcmodavg)
library(kableExtra)
library(ggeffects)

```

# Functions

Load custom functions for data organization, analysis and creation of graphs.

```{r}
source("Code/Functions_host.R")

```

# Host level data

Load the main data table

```{r}
T_coords <- read.csv("Data/Tcoords.csv") # coordinates table

#Main data table
H_data <- read.csv("Data/host_level.csv") %>%
  select(-X, -Count, -ID, -Trap, -Trapline) %>% 
  #Select only the rodents with available data
  filter(SM_Sp %in% c("ONLE", "PELE", "SIHI")) %>%
  #Drop table mising values
  drop_na() %>%
  #Create year as factor
  mutate(year= as.factor(year),
         Month= as.factor(Month)) %>%
  #Join with coordinates
  left_join(T_coords) %>% 
  #Create transect ID
  mutate(T_group= as.factor(gsub("[^0-9]", "", Transect) ),
         # Transform abundances to 1 and 0
         n= if_else(n>0, 1, n))

```

# Description of variables

Description of the used variables

```{r}
#| echo: false
library(readxl)
Table_variables <- read_excel("Table variables.xlsx")

kbl(Table_variables,
    caption = "Description of the variables used to model tick presence and load on small mammal hosts in South Texas, USA.",
    digits = 3) %>% 
  kable_styling(bootstrap_options = c("striped", "hover"), full_width = F)

```

# Modeling

## ***Onychomys leucogaster***

### ***Dermacentor*** **larvae**

```{r}
ONLE_D <- H_data %>% 
  filter(SM_Sp == "ONLE" & # Filter the rodent species
           Tick_gen %in% c("Dermacentor", "No tick") & # filter de tick sp
           Tick_Age %in% c("Larvae", "No tick") ) # filter the tick age
```

#### Random effects

To evaluate the random effects structure, models with all fixed variables and possible random structures were ranked: transect ID random intercept and a model with no random effects. Models were ranked using the Akaike information criterion corrected for small samples (AICc).

```{r}
OD_random <-random_eff(ONLE_D, binomial())
kbl(compare_performance(OD_random, metrics = "AICc"),
    digits = 3) %>% 
  kable_styling(bootstrap_options = c("striped", "hover"), full_width = F)


```

Subsequently, the residuals of the selected model are inspected.

```{r}
#| fig-cap: "Inspection of model residuals without random effects"
OD_norandom_res <- simulateResiduals(OD_random$norandom, plot = T)
```

#### Evaluate any spatial correlation issues

Inspection of the spatial autocorrelation of residuals of the selected model.

```{r}
#| layout-ncol: 2
#| fig-cap: 
#|   -"Fall 2019 correlogram"
#|   -"Fall 2020 correlogram"
#|   -"Winter 2019 correlogram"
#|   -"Winter 2020 correlogram"
  
OD_audf <- get_sau_data(OD_norandom_res, ONLE_D)
OD_groups <- split(OD_audf, OD_audf$survey)
OD_corelog <- map(OD_groups, get_correlog) %>% compact()
OD_corelogbase <- map(OD_corelog, get_correlog_base)
OD_corelogplot <- map(OD_corelogbase, get_correlogplot)
OD_corelogplot$Fall_2019
OD_corelogplot$Fall_2020
OD_corelogplot$Winter_2019
OD_corelogplot$Winter_2020

```

According to the inspection of the residuals, the selected model showed adequate goodness-of-fit and non spatial autocorrelation problems.

#### Host variables

Univariate models were created for the selection of host variables. The models were ranked using AICc. Variables from models with Delta \<2 were used in the final model selection.

```{r}
OD_H <- get_H_models(ONLE_D, family =binomial(),zi = FALSE)
  
OD_H_sel <-aictab(OD_H, sort = T)

kbl(OD_H_sel,
    caption = "Model selection table for host variables",
    digits = 3) %>% 
  kable_styling(bootstrap_options = c("striped", "hover"), full_width = F)

```

#### Habitat variables

Univariate models were created for the selection of habitat variables. The models were ranked using AICc. Variables from models with Delta \<2 were used in the final model selection.

```{r}
OD_Ha <- get_Hamodels(ONLE_D, family = binomial(),zi = FALSE)
  
OD_Ha_sel <- aictab(OD_Ha, sort = T)

  kbl(OD_Ha_sel,
    caption = "Model selection table for habitat variables",
    digits = 3) %>% 
  kable_styling(bootstrap_options = c("striped", "hover"), full_width = F)
```

#### Weather variables

Univariate models were created for the selection of weather variables. The models were ranked using AICc. Variables from models with Delta \<2 were used in the final model selection.

```{r}
OD_W <- get_Wmodels(ONLE_D, family = binomial(),zi = FALSE)

OD_W_sel <- aictab(OD_W, sort = T)

  kbl(OD_W_sel,
    caption = "Model selection table for  weather variables",
    digits = 3) %>% 
  kable_styling(bootstrap_options = c("striped", "hover"), full_width = F)
```

#### Final selection

With the variables selected above, we generated candidate models of their possible interactions. We ranked them using AICc.

```{r}
OD_models <- list(
OD_m1 <- glmmTMB(n~ Rep,                       family =binomial, data = ONLE_D),
OD_m2 <- glmmTMB(n~ Rep+ Sex,                  family =binomial, data = ONLE_D),
OD_m3 <- glmmTMB(n~ Rep+ Wght,                 family =binomial, data = ONLE_D),
OD_m4 <- glmmTMB(n~ Rep+ Avg_LC,               family =binomial, data = ONLE_D),
OD_m5 <- glmmTMB(n~ Rep+ Season,               family =binomial, data = ONLE_D),
OD_m6 <- glmmTMB(n~ Rep* Sex,                  family =binomial, data = ONLE_D),
OD_m7 <- glmmTMB(n~ Rep* Wght,                 family =binomial, data = ONLE_D),
OD_m8 <- glmmTMB(n~ Rep* Avg_LC,               family =binomial, data = ONLE_D),
OD_m9 <- glmmTMB(n~ Rep* Season,               family =binomial, data = ONLE_D),

OD_m10 <- glmmTMB(n~ Sex,                       family =binomial, data = ONLE_D),
OD_m11 <- glmmTMB(n~ Sex+ Wght,                 family =binomial, data = ONLE_D),
OD_m12 <- glmmTMB(n~ Sex+ Avg_LC,               family =binomial, data = ONLE_D),
OD_m13 <- glmmTMB(n~ Sex+ Season,               family =binomial, data = ONLE_D),
OD_m14 <- glmmTMB(n~ Sex* Wght,                 family =binomial, data = ONLE_D),
OD_m15 <- glmmTMB(n~ Sex* Avg_LC,               family =binomial, data = ONLE_D),
OD_m16 <- glmmTMB(n~ Sex* Season,               family =binomial, data = ONLE_D),

OD_m17 <- glmmTMB(n~ Wght,                       family =binomial, data = ONLE_D),
OD_m18 <- glmmTMB(n~ Wght+ Avg_LC,               family =binomial, data = ONLE_D),
OD_m19 <- glmmTMB(n~ Wght+ Season,               family =binomial, data = ONLE_D),
OD_m20 <- glmmTMB(n~ Wght* Avg_LC,               family =binomial, data = ONLE_D),
OD_m21 <- glmmTMB(n~ Wght* Season,               family =binomial, data = ONLE_D),


OD_m22 <- glmmTMB(n~ Avg_LC,                    family =binomial, data = ONLE_D),
OD_m23 <- glmmTMB(n~ Avg_LC+ Season,            family =binomial, data = ONLE_D),
OD_m24 <- glmmTMB(n~ Avg_LC* Season,            family =binomial, data = ONLE_D),

OD_m25 <- glmmTMB(n~ Rain,                    family =binomial, data = ONLE_D),
OD_m26 <- glmmTMB(n~ Rain+ Season,            family =binomial, data = ONLE_D),
OD_m27 <- glmmTMB(n~ Rain* Season,            family =binomial, data = ONLE_D),

OD_m28 <- glmmTMB(n~ Season,                    family =binomial, data = ONLE_D),

OD_m29 <- glmmTMB(n~  1,                       family =binomial, data = ONLE_D),

OD_m30 <- glmmTMB(n~ Month,                    family =binomial, data = ONLE_D),
OD_m31 <- glmmTMB(n~ Month*Rep,                family =binomial, data = ONLE_D),
OD_m32 <- glmmTMB(n~ Month*Sex,                family =binomial, data = ONLE_D)

)


OD_sel <- aictab(OD_models, sort = F)

OD_sel_table <- get_table_models(OD_models, OD_sel, "Prevalence")


  kbl(OD_sel_table,
    caption = "Final model selection to identify drivers influencing tick presence in Onychomys leucogaster",
    digits = 2) %>% 
  kable_styling(bootstrap_options = c("striped", "hover"), full_width = F)
```

We inspected the 85% confidence intervals of the regression coefficients of the selected models.

```{r}
OD_final_best <- list(OD_m22=OD_m22, OD_m20=OD_m20, OD_m4=OD_m4, OD_m12=OD_m12, OD_m15=OD_m15, OD_m18=OD_m18)

OD_final_best_ci <- map2_df(names(OD_final_best), OD_final_best, get_ci)

 kbl(OD_final_best_ci,
    caption = "Selected models confidence interval table",
    digits = 2) %>% 
  kable_styling(bootstrap_options = c("striped", "hover"), full_width = F)

```

```{r}
ci_plot(OD_final_best_ci)+ labs(title= "Onychomys leucogaster models 85%CI")
```

Model 22 (Tick Prevalence \~ Avg_LC) and 20 (Tick Prevalence \~ Wght \* Avg_LC ) contain informative estimations

#### Selected models

Subsequently, we inspect the residuals of the models whose confidence intervals do not overlap 0.

```{r}
#| layout-ncol: 2
#| fig-cap: 
#|  - "Residual inspection of model Tick Prevalence ~ Avg_LC"
#|  - "Residual inspection of model Tick Prevalence ~ Wght * Avg_LC"

OD_res <- lapply(list(OD_m22, OD_m20), simulateResiduals, plot= TRUE)


```

We build the summary table of the selected models

```{r}
OD_1_sum <- model_parameters(OD_m20, digits=2, ci= 0.85) %>% 
  mutate(Family= "Bernoulli",
         Model= "Model 20") %>% 
  select(-SE, -z, -df_error)
OD_2_sum <- model_parameters(OD_m22, digits=2, ci= 0.85) %>% 
  mutate(Family= "Bernoulli",
         Model= "Model 22") %>% 
  select(-SE, -z, -df_error)

kbl(rbind(OD_1_sum, OD_2_sum),
    caption = "O. leocogaster - Dermacentor",
    digits = 3) %>% 
  kable_styling(bootstrap_options = c("striped", "hover"), full_width = F)
```

#### Prediction plots

```{r}
#| layout-ncol: 2
#| fig-cap: 
#|   -"Prediction plot of model Tick Prevalence ~ Avg_LC"
#|   -"Prediction plot of model Tick Prevalence ~ Wght * Avg_LC"

OD_Pred1 <- ggeffect(OD_m22, 
                     terms = c("Avg_LC"), 
                     ci_level = 0.85)

OD_Pred2 <- ggeffect(OD_m20, 
                     terms = c("Wght", "Avg_LC"), 
                     ci_level = 0.85)


OD_pred1_plot <- conpred_plot(OD_Pred1, title = NULL,
                              "Average litter cover (%)",
                              ONLE_D, var = Avg_LC,
                              fill = "#A2CD5A")+
  labs(y= "Tick presence probability")

OD_pred2_plot <- ggplot()+
  geom_ribbon(data= OD_Pred2, aes(x= x, y=predicted,
                                  ymin=conf.low, ymax=conf.high,
                                  fill=group), alpha= 0.4)+
  geom_line(data= OD_Pred2, aes(x= x, y= predicted, col= group),
            linewidth= 1)+
  labs(x= "Host weight (g)", y= "Tick presence probability",
       fill= "Average litter cover (%)",
       col= "Average litter cover (%)")+
    guides(col= guide_legend(position = "inside"),
         fill= guide_legend(position = "inside"))+
    scale_color_viridis_d()+
    scale_fill_viridis_d()+
    theme_bw(base_size = 12)+
    theme(legend.position.inside = c(0.7, 0.8),
          legend.background = element_blank())

OD_pred1_plot
OD_pred2_plot 

```

## ***Peromyscus leucopus***

### ***Dermacentor*** larvae

```{r}
PELE_D <- H_data %>% 
  filter(SM_Sp == "PELE" & 
           Tick_gen %in% c("Dermacentor", "No tick") & 
           Tick_Age %in% c("Larvae", "No tick") )
```

#### Random effects

To evaluate the random effects structure, models with all fixed variables and possible random structures were ranked: transect ID random intercept and a model with no random effects. Models were ranked using the Akaike information criterion corrected for small samples (AICc).

```{r}
PD_random <- random_eff(PELE_D, binomial())
kbl(compare_performance(PD_random, metrics = "AICc"),
    digits = 3) %>% 
  kable_styling(bootstrap_options = c("striped", "hover"), full_width = F)

```

Residual inspection

```{r}
PD_norandom_res <- simulateResiduals(PD_random$norandom, plot = T)
```

#### Evaluate any spatial correlation issues

```{r}
#| layout-ncol: 2
#| fig-cap: 
#|  -"Fall 2019 correlogram"
#|  -"Fall 2020 correlogram"
#|  -"Spring 2020 correlogram"
#|  -"Winter 2019 correlogram"
#|  -"Winter 2020 correlogram"

PD_audf <- get_sau_data(PD_norandom_res, PELE_D)
PD_groups <- split(PD_audf, PD_audf$survey)
PD_corelog <- map(PD_groups, get_correlog)
PD_corelogbase <- map(PD_corelog, get_correlog_base)
PD_corelogplot <- map(PD_corelogbase, get_correlogplot)
PD_corelogplot
```

According to the inspection of the residuals, the selected model showed adequate goodness-of-fit and non spatial autocorrelation problems.

#### Host variables

Univariate models were created for the selection of host variables. The models were ranked using AICc. Variables from models with Delta \<2 were used in the final model selection.

```{r}
PD_H <-  get_H_models(PELE_D, family = binomial(), zi= FALSE)

PD_H_sel <- aictab(PD_H, sort = T)
kbl(PD_H_sel,
    caption = "Model selection table for host variables",
    digits = 3) %>% 
  kable_styling(bootstrap_options = c("striped", "hover"), full_width = F)

```

#### Habitat variables

Univariate models were created for the selection of habitat variables. The models were ranked using AICc. Variables from models with Delta \<2 were used in the final model selection.

```{r}
PD_Ha <- get_Hamodels(PELE_D, family = binomial, zi= FALSE)

PD_Ha_sel <- aictab(PD_Ha, sort = T)

  kbl(PD_Ha_sel,
    caption = "Model selection table for habitat variables",
    digits = 3) %>% 
  kable_styling(bootstrap_options = c("striped", "hover"), full_width = F)
```

#### Weather variables

Univariate models were created for the selection of weather variables. The models were ranked using AICc. Variables from models with Delta \<2 were used in the final model selection.

```{r}
PD_W <- get_Wmodels(PELE_D, family = binomial(), zi= FALSE)

PD_W_sel <- aictab(PD_W, sort = T)
kbl(PD_W_sel,
    caption = "Model selection table for habitat variables",
    digits = 3) %>% 
  kable_styling(bootstrap_options = c("striped", "hover"), full_width = F)
```

#### Final selection

With the variables selected above, we generated candidate models of their possible interactions. We ranked them using AICc.

```{r}
PD_models <- list(
PD_m1  <- glmmTMB(n~ Rep,                      family =binomial, data = PELE_D),
PD_m2  <- glmmTMB(n~ Rep+ Sex,                 family =binomial, data = PELE_D),
PD_m3  <- glmmTMB(n~ Rep+ Wght,                family =binomial, data = PELE_D),
PD_m4  <- glmmTMB(n~ Rep+ Wght+I(Wght^2),      family =binomial, data = PELE_D),
PD_m5  <- glmmTMB(n~ Rep+ Avg_VH,              family =binomial, data = PELE_D),
PD_m6  <- glmmTMB(n~ Rep+ Avg_VH+I(Avg_VH^2),  family =binomial, data = PELE_D),
PD_m7  <- glmmTMB(n~ Rep+ Avg_LC,              family =binomial, data = PELE_D),
PD_m8  <- glmmTMB(n~ Rep+ Temp+I(Temp^2),      family =binomial, data = PELE_D),
PD_m9  <- glmmTMB(n~ Rep* Sex,                 family =binomial, data = PELE_D),
PD_m10  <- glmmTMB(n~ Rep* Wght,                family =binomial, data = PELE_D),
PD_m11  <- glmmTMB(n~ Rep* (Wght+I(Wght^2)),    family =binomial, data = PELE_D),
PD_m12  <- glmmTMB(n~ Rep* Avg_VH,              family =binomial, data = PELE_D),
PD_m13 <- glmmTMB(n~ Rep* (Avg_VH+I(Avg_VH^2)),family =binomial, data = PELE_D),
PD_m14  <- glmmTMB(n~ Rep* Avg_LC,              family =binomial, data = PELE_D),
PD_m15 <- glmmTMB(n~ Rep* (Temp+I(Temp^2)),    family =binomial, data = PELE_D),

PD_m16  <- glmmTMB(n~ Sex,                      family =binomial, data = PELE_D),
PD_m17  <- glmmTMB(n~ Sex+ Wght,                family =binomial, data = PELE_D),
PD_m18 <- glmmTMB(n~ Sex+ Wght+I(Wght^2),      family =binomial, data = PELE_D),
PD_m19  <- glmmTMB(n~ Sex+ Avg_VH,              family =binomial, data = PELE_D),
PD_m20  <- glmmTMB(n~ Sex+ Avg_VH+I(Avg_VH^2),  family =binomial, data = PELE_D),
PD_m21 <- glmmTMB(n~ Sex+ Avg_LC,              family =binomial, data = PELE_D),
PD_m22 <- glmmTMB(n~ Sex+ Temp+I(Temp^2),      family =binomial, data = PELE_D),
PD_m23 <- glmmTMB(n~ Sex* Wght,                family =binomial, data = PELE_D),
PD_m24 <- glmmTMB(n~ Sex* (Wght+I(Wght^2)),    family =binomial, data = PELE_D),
PD_m25 <- glmmTMB(n~ Sex* Avg_VH,              family =binomial, data = PELE_D),
PD_m26 <- glmmTMB(n~ Sex* (Avg_VH+I(Avg_VH^2)),family =binomial, data = PELE_D),
PD_m27 <- glmmTMB(n~ Sex* Avg_LC,              family =binomial, data = PELE_D),
PD_m28 <- glmmTMB(n~ Sex* (Temp+I(Temp^2)),    family =binomial, data = PELE_D),


PD_m29 <- glmmTMB(n~ Wght,                        family =binomial, data = PELE_D),
PD_m30 <- glmmTMB(n~ Wght+ Avg_VH,                family =binomial, data = PELE_D),
PD_m31 <- glmmTMB(n~ Wght+ Avg_VH+I(Avg_VH^2),    family =binomial, data = PELE_D),
PD_m32 <- glmmTMB(n~ Wght+ Avg_LC,                family =binomial, data = PELE_D),
PD_m33 <- glmmTMB(n~ Wght+ Temp+I(Temp^2),        family =binomial, data = PELE_D),
PD_m34 <- glmmTMB(n~ Wght* Avg_VH,                family =binomial, data = PELE_D),
PD_m35 <- glmmTMB(n~ Wght* Avg_LC,                family =binomial, data = PELE_D),
PD_m36 <- glmmTMB(n~ Wght* (Temp+I(Temp^2)),      family =binomial, data = PELE_D),

PD_m37 <- glmmTMB(n~ Wght+I(Wght^2),              family =binomial, data = PELE_D),

PD_m38 <- glmmTMB(n~ Wght+I(Wght^2)+ Avg_VH,      family =binomial, data = PELE_D),
PD_m39 <- glmmTMB(n~ Wght+I(Wght^2)+ Avg_VH+I(Avg_VH^2),family =binomial, data = PELE_D),
PD_m40 <- glmmTMB(n~ Wght+I(Wght^2)+ Avg_LC,      family =binomial, data = PELE_D),
PD_m41 <- glmmTMB(n~ Wght+I(Wght^2)+ Temp+I(Temp^2), family =binomial, data = PELE_D),
PD_m42 <- glmmTMB(n~ (Wght+I(Wght^2))* Avg_VH,    family =binomial, data = PELE_D),
PD_m43 <- glmmTMB(n~ Wght+I(Wght^2)* Avg_LC,      family =binomial, data = PELE_D),

PD_m44 <- glmmTMB(n~ Avg_VH,                      family =binomial, data = PELE_D),
PD_m45 <- glmmTMB(n~ Avg_VH+ Avg_LC,              family =binomial, data = PELE_D),
PD_m46 <- glmmTMB(n~ Avg_VH+ Temp+I(Temp^2),      family =binomial, data = PELE_D),
PD_m47 <- glmmTMB(n~ Avg_VH* Avg_LC,              family =binomial, data = PELE_D),
PD_m48 <- glmmTMB(n~ Avg_VH* (Temp+I(Temp^2)),    family =binomial, data = PELE_D),

PD_m49 <- glmmTMB(n~ Avg_VH+ I(Avg_VH^2),         family =binomial, data = PELE_D),
PD_m50 <- glmmTMB(n~ Avg_VH+ I(Avg_VH^2)+Avg_LC,  family =binomial, data = PELE_D),
PD_m51 <- glmmTMB(n~ Avg_VH+ I(Avg_VH^2)+ Temp+I(Temp^2),family =binomial, data = PELE_D),

PD_m52 <- glmmTMB(n~ Avg_LC,                      family =binomial, data = PELE_D),
PD_m53 <- glmmTMB(n~ Avg_LC+ Temp+I(Temp^2),      family =binomial, data = PELE_D),
PD_m54 <- glmmTMB(n~ Avg_LC* Temp+I(Temp^2),      family =binomial, data = PELE_D),

PD_m55 <- glmmTMB(n~ Temp+I(Temp^2),              family =binomial, data = PELE_D),

PD_m56 <- glmmTMB(n~ 1,                           family =binomial, data = PELE_D),

PD_m57 <- glmmTMB(n~ Month,                       family =binomial, data = PELE_D),
PD_m58 <- glmmTMB(n~ Month *Rep,                  family =binomial, data = PELE_D),
PD_m59 <- glmmTMB(n~ Month* Sex,                  family =binomial, data = PELE_D),
PD_m60 <- glmmTMB(n~ Month* Wght,                 family =binomial, data = PELE_D),
PD_m61 <- glmmTMB(n~ Month* (Wght+I(Wght^2)),     family =binomial, data = PELE_D),
PD_m62 <- glmmTMB(n~ Month* Avg_LC,               family =binomial, data = PELE_D),
PD_m63 <- glmmTMB(n~ Month* (Temp+I(Temp^2)),     family =binomial, data = PELE_D)
)

PD_sel <- aictab(PD_models, sort = F)

PD_sel_table <- get_table_models(PD_models, PD_sel, "Prevalence")

  kbl(PD_sel_table,
    caption = "Final model selection to identify drivers influencing tick presence in Peromyscus leucopus",
    digits = 2) %>% 
  kable_styling(bootstrap_options = c("striped", "hover"), full_width = F)
```

We inspected the 85% confidence intervals of the regression coefficients of the selected models.

```{r}
PD_final_best <- list(PD_m57=PD_m57)

PD_final_best_ci <- map2_df(names(PD_final_best), PD_final_best, get_ci)

 kbl(PD_final_best_ci,
    caption = "Selected models confidence interval table",
    digits = 2) %>% 
  kable_styling(bootstrap_options = c("striped", "hover"), full_width = F)


```

```{r}
ci_plot(PD_final_best_ci)+ labs(title= "Peromyscus leucopus models 85%CI")
```

#### Selcted models

Subsequently, we inspect the residuals of the models whose confidence intervals do not overlap 0.

```{r}
PD_res <- simulateResiduals(PD_m57, plot=T)


```

We build the summary table of the selected models

```{r}
PD_1_sum <- model_parameters(PD_m57, digits=2, ci= 0.85) %>% 
  mutate(Family= "Bernoulli",
         Model= "Model 57") %>% 
  select(-SE, -z, -df_error)


kbl(PD_1_sum,
    caption = "P. leocopus - Dermacentor",
    digits = 3) %>% 
  kable_styling(bootstrap_options = c("striped", "hover"), full_width = F)
```

#### Prediction plots

```{r}
PD_Pred1 <- ggeffect(PD_m57, terms = c("Month"), ci_level = 0.85)

(PD_pred1_plot <- catpred2_plot(PD_Pred1, title= NULL, 
                               "Month", 
                               PELE_D, cat=Month, 
                               col = "deepskyblue2")+
  labs(y="Tick presence probability" ))


```

## ***Sigmodon hispidus***

### ***Dermacentor*** larvae

```{r}
SIHI_D <- H_data %>% 
  filter(SM_Sp == "SIHI" & 
           Tick_gen %in% c("Dermacentor", "No tick") & 
           Tick_Age %in% c("Larvae", "No tick") )
```

#### Random effects

To evaluate the random effects structure, models with all fixed variables and possible random structures were ranked: transect ID random intercept and a model with no random effects. Models were ranked using the Akaike information criterion corrected for small samples (AICc).

```{r}
SD_random <- random_eff(SIHI_D, binomial)
kbl(compare_performance(SD_random, metrics = "AICc"),
    digits = 3) %>% 
  kable_styling(bootstrap_options = c("striped", "hover"), full_width = F)
```

Subsequently, the residuals of the selected model are inspected.

```{r}
SD_norandom_res <- simulateResiduals(SD_random$norandom, plot = T)


```

#### Evaluate any spatial correlation issues

```{r}
#| layout-ncol: 2
#| fig-cap: 
#|   -"Fall 2019 correlogram"
#|   -"Fall 2020 correlogram"
#|   -"Spring 2020 correlogram"
#|   -"Winter 2019 correlogram"
#|   -"Winter 2020 correlogram"
SD_audf <- get_sau_data(SD_norandom_res, SIHI_D)
SD_groups <- split(SD_audf, SD_audf$survey)
SD_corelog <- map(SD_groups, get_correlog) %>% compact()
SD_corelogbase <- map(SD_corelog, get_correlog_base)
SD_corelogplot <- map(SD_corelogbase, get_correlogplot)
SD_corelogplot
```

According to the inspection of the residuals, the selected model showed adequate goodness-of-fit or spatial autocorrelation problems.

#### Host variables

Univariate models were created for the selection of host variables. The models were ranked using AICc. Variables from models with Delta \<2 were used in the final model selection.

```{r}
SD_H <-  get_H_models(SIHI_D, family = binomial(), zi= FALSE)

SD_H_sel <-aictab(SD_H, sort = T)

kbl(SD_H_sel,
    caption = "Model selection table for host variables",
    digits = 3) %>% 
  kable_styling(bootstrap_options = c("striped", "hover"), full_width = F)

```

#### Habitat variables

Univariate models were created for the selection of habitat variables. The models were ranked using AICc. Variables from models with Delta \<2 were used in the final model selection.

```{r}
SD_Ha <- get_Hamodels(SIHI_D, family = binomial, zi= FALSE)
SD_Ha_sel <- aictab(SD_Ha, sort = T)
  kbl(SD_Ha_sel,
    caption = "Model selection table for habitat variables",
    digits = 3) %>% 
  kable_styling(bootstrap_options = c("striped", "hover"), full_width = F)
```

#### Weather variables

Univariate models were created for the selection of weather variables. The models were ranked using AICc. Variables from models with Delta \<2 were used in the final model selection.

```{r}
SD_W <- get_Wmodels(SIHI_D, family = binomial, zi= FALSE)
SD_W_sel <-aictab(SD_W, sort = T)
  kbl(SD_W_sel,
    caption = "Model selection table for  weather variables",
    digits = 3) %>% 
  kable_styling(bootstrap_options = c("striped", "hover"), full_width = F)
```

#### Final selection

With the variables selected above, we generated candidate models of their possible interactions. We ranked them using AICc.

```{r}
SD_models <- list(

SD_m1 <- glmmTMB(n~ Rep,                          family =binomial, data = SIHI_D),
SD_m2 <- glmmTMB(n~ Rep+ Avg_VH+I(Avg_VH^2),      family =binomial, data = SIHI_D),
SD_m3 <- glmmTMB(n~ Rep+ Season,                  family =binomial, data = SIHI_D),
SD_m4 <- glmmTMB(n~ Rep* Season,                  family =binomial, data = SIHI_D),

SD_m5 <- glmmTMB(n~ Avg_VH+I(Avg_VH^2),          family =binomial, data = SIHI_D),
SD_m6 <- glmmTMB(n~ Avg_VH+I(Avg_VH^2)+ Season,  family =binomial, data = SIHI_D),
SD_m7 <- glmmTMB(n~ Avg_VH+I(Avg_VH^2)* Season,  family =binomial, data = SIHI_D),

SD_m8 <- glmmTMB(n~ Season,                      family =binomial, data = SIHI_D),

SD_m9 <- glmmTMB(n~ 1,                           family =binomial, data = SIHI_D),

SD_m10 <- glmmTMB(n~ Month,                       family =binomial, data = SIHI_D),
SD_m11 <- glmmTMB(n~ Month+Rep,                   family =binomial, data = SIHI_D),
SD_m12 <- glmmTMB(n~ Month*Rep,                   family =binomial, data = SIHI_D)


)


SD_sel <- aictab(SD_models, sort = F)
SD_sel_table <- get_table_models(SD_models, SD_sel, "Prevalence")


  kbl(SD_sel_table,
    caption = "Final model selection to identify drivers influencing tick presence in Sigmodon hispidus",
    digits = 2) %>% 
  kable_styling(bootstrap_options = c("striped", "hover"), full_width = F)
```

We inspected the 85% confidence intervals of the regression coefficients of the selected models.

```{r}
SD_final_best <- list(SD_m2=SD_m2, SD_m7=SD_m7, SD_m6=SD_m6)

SD_final_best_ci <- map2_df(names(SD_final_best), SD_final_best, get_ci)

 kbl(SD_final_best_ci,
    caption = "Selected models confidence interval table",
    digits = 2) %>% 
  kable_styling(bootstrap_options = c("striped", "hover"), full_width = F)


```

```{r}
ci_plot(SD_final_best_ci)+ labs(title= "Sigmodon hispidus models 85%CI")
```

All models had fairly wide confidence intervals O were not possible to estimate in the SD_m24 model. In all cases it was not possible to obtain a reliable estimate of the regression coefficients.

#### Selected models

Subsequently, we inspect the residuals of the models whose confidence intervals do not overlap 0.

```{r}
#| layout-ncol: 2
#| fig-cap: 
#|  - "Residual inspection of model Tick Prevalence ~ Rep + Avg_VH + I(Avg_VH^2)"
#|  - "Residual inspection of model Tick Prevalence ~ Avg_VH + I(Avg_VH^2) * Season"

SD_res <- lapply(list(SD_m2, SD_m7), simulateResiduals, plot= TRUE)


```

Both models present variance homogeneity problems. Since the bernoulli distribution has fixed dispersion, it is not possible to model this variation. However, the residual plot shows that the deviation from the quartile is not so severe.

#### Prediction plots

```{r}
#| layout-ncol: 2
#| fig-cap: 
#|  -"Prediction plot of model Tick Prevalence ~ Rep + Avg_VH + I(Avg_VH^2)"
#|  -"Prediction plot of model Tick Prevalence ~ Avg_VH + I(Avg_VH^2) * Season"

SD_Pred1 <- ggeffect(SD_m2, terms = c("Avg_VH", "Rep"), ci_level = 0.85)

SD_Pred2 <- ggeffect(SD_m7, terms = c("Avg_VH","Season"), ci_level = 0.85)


SD_pred1_plot <- ggplot()+
  geom_ribbon(data= SD_Pred1, aes(x= x, y=predicted,
                                  ymin=conf.low, ymax=conf.high,
                                  fill=group), alpha= 0.4)+
   geom_line(data= SD_Pred1, aes(x= x, y= predicted, col= group),
            linewidth= 1)+
  labs(x= "Average vegetation height", y= "Tick presence probability",
       fill= "Reproductive activity",
       col= "Reproductive activity")+
    guides(col= guide_legend(position = "inside"),
         fill= guide_legend(position = "inside"))+
    scale_color_viridis_d()+
    scale_fill_viridis_d()+
    theme_bw(base_size = 12)+
    theme(legend.position.inside = c(0.7, 0.8),
          legend.background = element_blank())

SD_pred2_plot <- ggplot()+
  geom_ribbon(data= SD_Pred2, aes(x= x, y=predicted,
                                  ymin=conf.low, ymax=conf.high,
                                  fill=group), alpha= 0.4)+
   geom_line(data= SD_Pred2, aes(x= x, y= predicted, col= group),
            linewidth= 1)+
  labs(x= "Average vegetation height", y= "Tick presence probability",
       fill= "Month",
       col= "Month")+
    guides(col= guide_legend(position = "inside"),
         fill= guide_legend(position = "inside"))+
    scale_color_viridis_d()+
    scale_fill_viridis_d()+
    theme_bw(base_size = 12)+
    theme(legend.position.inside = c(0.7, 0.8),
          legend.background = element_blank())

SD_pred1_plot
SD_pred2_plot
```

# Final plots

```{r}
#| fig-height: 8.5
#| fig-width: 9

(predplots <- (OD_pred1_plot+ OD_pred2_plot +PD_pred1_plot)+
   plot_layout(ncol = 2, axes = "collect")+
   plot_annotation(tag_levels = 'A'))


ggsave(plot = predplots, filename = "Figs/Prevalence_predplots.png",
       width=9, height=8.5)

ggsave(plot = predplots, filename = "Figs/Prevalence_predplots.svg",
       width=9, height=8.5)
```
```
