# Supplementary material for: Different drivers, same tick: Effect of host traits, habitat, and climate on the infestation of three rodent species by larval Dermacentor ticks
Source: Int J Parasitol Parasites Wildl. 2025 Mar 7;26:101054. doi: 10.1016/j.ijppaw.2025.101054 (PMC11929885; doi:10.1016/j.ijppaw.2025.101054)
Supplement: Multimedia component 2 [file mmc2.zip › Supplementary File 2_revised.html]

Statistical modeling process for the analysis of the load of Dermacentor larvae in two species of small rodents.


## Table of contents

- Packages
- Functions
- Host level data
- Description of variables
- Modeling
  - ***O. leucogaster***
    - ***Dermacentor*** **larvae**
  - ***Peromyscus leucopus***
    - ***Dermacentor*** larvae
  - ***Sigmodon hispidus***
    - \***Dermacentor** larvae
- Final Plots

# Statistical modeling process for the analysis of the load of Dermacentor larvae in two species of small rodents.

 Code

- Show All Code
- Hide All Code
- ---
- View Source

# Packages

List of packages used

Code

```
library(tidyverse)
library(glmmTMB)
library(performance)
library(modelbased)
library(DHARMa)
library(patchwork)
library(parameters)
library(AICcmodavg)
library(kableExtra)
library(ggeffects)
```

# Functions

Load custom functions for data organization, analysis and creation of graphs.

Code

```
source("Code/Functions_host.R")
```

# Host level data

Load the main data table

Code

```
T_coords <- read.csv("Data/Tcoords.csv") # coordinate table

#Main data table
H_data <- read.csv("Data/host_level.csv") %>%
  select(-X, -Count, -ID, -Trap, -Trapline, -Date ) %>% 
  #Select only the usable rodents
  filter(SM_Sp %in% c("ONLE", "PELE", "SIHI")) %>% 
  #Drop table mising values
  drop_na() %>%
  #Create year as factor
  mutate(year= as.factor(year),
         Month= as.factor(Month)) %>% 
  #Join with coordinates
  left_join(T_coords) %>% 
  #Create transect ID
  mutate(T_group= as.factor(gsub("[^0-9]", "", Transect) ))
```

# Description of variables

Description of the used variables

Description of the variables used to model tick presence and load on small mammal hosts in South Texas, USA.

| Variable (Code) | Description | Variable type | Hypothesis | Expected relation |
| --- | --- | --- | --- | --- |
| Tick prevalence | Detection or non-detection of ticks in each rodent individual. | Response variable | NA | NA |
| Tick load | The number of tick counts for each rodent individual captured | Response variable | NA | NA |
| Body weight (g) (Wght) | The weight of each rodent individual captured (g) | Host - Continuum | Hosts with greater body size represent more habitat and resources for ticks (Kuris et al., 1980; Poulin, 2004). Therefore, bigger individuals will favor greater presence and number of ticks. | Linear / Quadratic |
| Sex (Sex) | The sex of each rodent captured | Host - Categorical : Male (M), Female (F) | Male mice tend to move more in the landscape compared to females. Therefore, males are expected to have higher tick burdens than females (Butler et al., 2020; Ostfeld et al., 1996). | NA |
| Reproductive status (Rep) | The reproductive status of each captured rodent. Reproductive and non-reproductive | Host - Categorical: non-reproductive (N), reproductive (Y) | The hormonal profile and the higher activity of sexually active mice make them more susceptible to a higher parasite load. Therefore, we expect that sexually active mice will have a higher chance of having a greater tick load (Ostfeld et al., 1996; Schmidt et al., 1999). | NA |
| Average litter cover (Avg\_LC) | Percent (%) presence of litter cover in each transect | Habitat - Continuum | Higher numbers of questing tick larvae are associated with areas with higher leaf litter cover, as these locations allow them to avoid adverse environmental conditions (Sonenshine, 2005). We expect that individual mice captured in areas of higher cover and depth of leaf litter will have higher tick larvae burdens. | Linear |
| Average litter depth (Avg\_LD) | Average of litter depth in each transect (cm) | Habitat - Continuum | NA | Linear |
| Average Herbaceous material (Avg\_HC) | Percent (%) presence of Herbaceous in each transect | Habitat - Continuum | Herbaceous cover favors microclimatic conditions for tick larval survival and therefore tick abundance (Sonenshine and Stout, 1968). We expect that mice captured in areas with higher grass cover will have higher tick burdens. | Linear |
| Average Canopy Cover (Avg\_CC) | Percent (%) of canopy coverture in each transect | Habitat - Continuum | Greater canopy cover increases humidity and reduces direct sunlight on the ground, which favors climatic conditions for tick larval development (Ginsberg et al., 2020; Leal et al., 2018; Zamora et al., 2020). We expect that individual mice captured in areas with higher canopy cover will have higher tick burdens. | Linear |
| Average vegetation height (Avg\_VH) | Average vegetation height for each transects (cm) | Habitat - Continuum | Tick larvae prefer vegetation with a height of less than 50 cm because it favors climatic conditions for their development (Prusinski et al., 2006). For this reason, we expect that captured mice will have higher tick loads in areas with lower vegetation height. | Linear/ Quadratic |
| Rain (Rain) | Rainfall measure (mm) for each day of the survey with Hobo data loggers | Weather - Continuum | Rainfall can negatively affect the development of tick larvae by washing and flooding microhabitats. In addition, a correlation has been reported between drier areas and periods of the year and tick larval abundance (Randolph and Storey, 1999). Therefore, we expect that rodent captured during periods of higher rainfall will have a lower parasite load. | Linear |
| Temperature average (Temp) | Temperature (C) measure for each day of the survey with Hobo data loggers | Weather - Continuum | Ticks show a peak of activity during warm weather periods and areas (Gilbert, 2010). However, extreme temperature or drought conditions may affect larval survival by desiccation (Teel et al., 2010). Therefore, we expect that mice captured during periods of very low or very high temperatures will have lower tick burdens. | Linea/ Quadratic |
| Season (Season) | The season in which the survey was conducted | Weather - Categorical: Spring, Fall and Winter | Ticks and their larvae exhibit seasonal peaks of activity (Brunner and Ostfeld, 2008). In the southern United States, tick larval activity patterns are greatest during the spring because weather conditions are more favorable than summer and winter (Chan and Kaufman, 2009; McEnroe, 1979). Therefore, we expect that rodent captured during the spring survey will have a higher tick load. | NA |
| Month (Month) | Month of the year in which the individuals were captured | Time control variable - Categorical: Month of the year represented in integer form 1: January to 12: December | Variable used as a control for the time effect. The month factor allows to identify influences of phenological, environmental or climatic dynamics that are not captured by the other variables used. | NA |
| Transect (Transect) | ID of the transect in which each individual was captured | Random effects variable | NA | NA |

# Modeling

## ***O. leucogaster***

### ***Dermacentor*** **larvae**

Code

```
ONLE_D <- H_data %>% 
  filter(SM_Sp == "ONLE" & 
           Tick_gen %in% c("Dermacentor", "No tick") & 
           Tick_Age %in% c("Larvae", "No tick") )
```

#### Distribution

We selected the most appropriate distribution for the analysis. Because of the count data, we used models that included all variables and used the Poisson, Negative Binomial, and Tweedie distributions. We used the Akaike information criterion for small samples, zero inflated test, and the over dispersion parameter to select the most appropriate distribution.

Code

```
OD_dis <- distri(ONLE_D)
kbl(compare_performance(OD_dis, metrics = "AICc"), digits = 3) %>% 
    kable_styling(bootstrap_options = c("striped", "hover"), full_width = F)
```

| Name | Model | AICc | AICc\_wt |
| --- | --- | --- | --- |
| PO | glmmTMB | 94.364 | NA |
| NB | glmmTMB | 95.691 | NA |
| TWD | glmmTMB | NA | NA |

Code

```
testDispersion(OD_dis$PO)
testDispersion(OD_dis$NB)
testDispersion(OD_dis$TWD)
```

-“Dispersion test for Poisson model” -“Dispersion test for Negative Binomial model” -“Dispersion test for Tweedie model”

```
    DHARMa nonparametric dispersion test via sd of residuals fitted vs.
    simulated

data:  simulationOutput
dispersion = 0.91179, p-value = 0.824
alternative hypothesis: two.sided
```

-“Dispersion test for Poisson model” -“Dispersion test for Negative Binomial model” -“Dispersion test for Tweedie model”

```
    DHARMa nonparametric dispersion test via sd of residuals fitted vs.
    simulated

data:  simulationOutput
dispersion = 0.5026, p-value = 0.872
alternative hypothesis: two.sided
```

-“Dispersion test for Poisson model” -“Dispersion test for Negative Binomial model” -“Dispersion test for Tweedie model”

```
    DHARMa nonparametric dispersion test via sd of residuals fitted vs.
    simulated

data:  simulationOutput
dispersion = 0.9097, p-value = 0.856
alternative hypothesis: two.sided
```

Code

```
testZeroInflation(OD_dis$PO)
testZeroInflation(OD_dis$NB)
```

-“Zero inflation test for Poisson model” -“Zero inflation test for Negative Binomial model”

```
    DHARMa zero-inflation test via comparison to expected zeros with
    simulation under H0 = fitted model

data:  simulationOutput
ratioObsSim = 1.0073, p-value = 0.944
alternative hypothesis: two.sided
```

-“Zero inflation test for Poisson model” -“Zero inflation test for Negative Binomial model”

```
    DHARMa zero-inflation test via comparison to expected zeros with
    simulation under H0 = fitted model

data:  simulationOutput
ratioObsSim = 1.0007, p-value = 1
alternative hypothesis: two.sided
```

Based on the test results, we choose the Binomial Negative family model.

#### Random effects

To evaluate the random effects structure, models with all fixed variables and possible random structures were ranked: transect ID random effect and a model with no random effects. Models were ranked using the Akaike information criterion corrected for small samples (AICc).

Code

```
OD_random <-random_eff(ONLE_D, nbinom2())

kbl(compare_performance(OD_random, metrics = "AICc"), digits = 3) %>% 
    kable_styling(bootstrap_options = c("striped", "hover"), full_width = F)
```

| Name | Model | AICc | AICc\_wt |
| --- | --- | --- | --- |
| Transect | glmmTMB | 105.925 | 0.219 |
| norandom | glmmTMB | 103.381 | 0.781 |

Residual inspection

Code

```
OD_norandom_res <- simulateResiduals(OD_random$norandom, plot = T)
```

Evaluate any spatial correlation issues

Code

```
OD_audf <- get_sau_data(OD_norandom_res, ONLE_D)
OD_groups <- split(OD_audf, OD_audf$survey)
OD_corelog <- map(OD_groups, get_correlog)%>% compact()
OD_corelogbase <- map(OD_corelog, get_correlog_base)
OD_corelogplot <- map(OD_corelogbase, get_correlogplot)
OD_corelogplot$Fall_2019
OD_corelogplot$Fall_2020
OD_corelogplot$Winter_2019
OD_corelogplot$Winter_2020
```

```
500  of  5000 
1000  of  5000 
1500  of  5000 
2000  of  5000 
2500  of  5000 
3000  of  5000 
3500  of  5000 
4000  of  5000 
4500  of  5000 
5000  of  5000 
500  of  5000 
1000  of  5000 
1500  of  5000 
2000  of  5000 
2500  of  5000 
3000  of  5000 
3500  of  5000 
4000  of  5000 
4500  of  5000 
5000  of  5000 
500  of  5000 
1000  of  5000 
1500  of  5000 
2000  of  5000 
2500  of  5000 
3000  of  5000 
3500  of  5000 
4000  of  5000 
4500  of  5000 
5000  of  5000 
500  of  5000 
1000  of  5000 
1500  of  5000 
2000  of  5000 
2500  of  5000 
3000  of  5000 
3500  of  5000 
4000  of  5000 
4500  of  5000 
5000  of  5000
```

-“Fall 2019 correlogram” -“Fall 2020 correlogram” -“Winter 2019 correlogram” -“Winter 2020 correlogram”

-“Fall 2019 correlogram” -“Fall 2020 correlogram” -“Winter 2019 correlogram” -“Winter 2020 correlogram”

-“Fall 2019 correlogram” -“Fall 2020 correlogram” -“Winter 2019 correlogram” -“Winter 2020 correlogram”

-“Fall 2019 correlogram” -“Fall 2020 correlogram” -“Winter 2019 correlogram” -“Winter 2020 correlogram”

According to the inspection of the residuals, the selected model showed adequate goodness-of-fit or spatial autocorrelation problems.

#### Host variables

Univariate models were created for the selection of host variables. The models were ranked using AICc. Variables from models with Delta <2 were used in the final model selection.

Code

```
OD_H <- get_H_models(ONLE_D, family =nbinom2(),zi = FALSE)
  
OD_H_sel <-aictab(OD_H, sort = T)

kbl(OD_H_sel,
    caption = "Model selection table for host variables",
    digits = 3) %>% 
  kable_styling(bootstrap_options = c("striped", "hover"), full_width = F)
```

Model selection table for host variables

|  | Modnames | K | AICc | Delta\_AICc | ModelLik | AICcWt | LL | Cum.Wt |
| --- | --- | --- | --- | --- | --- | --- | --- | --- |
| 3 | Sex | 3 | 92.259 | 0.000 | 1.000 | 0.518 | -43.029 | 0.518 |
| 1 | Weight | 3 | 94.033 | 1.774 | 0.412 | 0.213 | -43.916 | 0.731 |
| 4 | Rep | 3 | 94.474 | 2.215 | 0.330 | 0.171 | -44.137 | 0.903 |
| 2 | Weight2 | 4 | 95.599 | 3.341 | 0.188 | 0.097 | -43.632 | 1.000 |

#### Habitat variables

Univariate models were created for the selection of habitat variables. The models were ranked using AICc. Variables from models with Delta <2 were used in the final model selection.

Code

```
OD_Ha <- get_Hamodels(ONLE_D, family = nbinom2(),zi = FALSE)
OD_Ha_sel <- aictab(OD_Ha, sort = T)

kbl(OD_Ha_sel,
    caption = "Model selection table for habitat variables",
    digits = 3) %>% 
  kable_styling(bootstrap_options = c("striped", "hover"), full_width = F)
```

Model selection table for habitat variables

|  | Modnames | K | AICc | Delta\_AICc | ModelLik | AICcWt | LL | Cum.Wt |
| --- | --- | --- | --- | --- | --- | --- | --- | --- |
| 6 | Litter\_Cover | 3 | 84.457 | 0.000 | 1.000 | 0.804 | -39.128 | 0.804 |
| 4 | Vegetation\_height | 3 | 88.637 | 4.180 | 0.124 | 0.099 | -41.219 | 0.904 |
| 5 | Vegetation\_height2 | 4 | 89.481 | 5.025 | 0.081 | 0.065 | -40.573 | 0.969 |
| 3 | Canopy\_Cover | 3 | 92.165 | 7.708 | 0.021 | 0.017 | -42.982 | 0.986 |
| 1 | Litter\_depth | 3 | 93.606 | 9.149 | 0.010 | 0.008 | -43.703 | 0.994 |
| 2 | Herbaceus\_Cover | 3 | 94.261 | 9.804 | 0.007 | 0.006 | -44.031 | 1.000 |

#### Weather variables

Univariate models were created for the selection of weather variables. The models were ranked using AICc. Variables from models with Delta <2 were used in the final model selection.

Code

```
OD_W <- get_Wmodels(ONLE_D, family = nbinom2(),zi = FALSE)
OD_W_sel <- aictab(OD_W, sort = T)

kbl(OD_W_sel,
    caption = "Model selection table for  weather variables",
    digits = 3) %>% 
  kable_styling(bootstrap_options = c("striped", "hover"), full_width = F)
```

Model selection table for weather variables

|  | Modnames | K | AICc | Delta\_AICc | ModelLik | AICcWt | LL | Cum.Wt |
| --- | --- | --- | --- | --- | --- | --- | --- | --- |
| 3 | Rain | 3 | 92.600 | 0.000 | 1.000 | 0.504 | -43.200 | 0.504 |
| 1 | Temp | 3 | 94.131 | 1.531 | 0.465 | 0.235 | -43.965 | 0.739 |
| 4 | Season | 4 | 94.771 | 2.171 | 0.338 | 0.170 | -43.217 | 0.909 |
| 2 | Temp2 | 4 | 96.028 | 3.428 | 0.180 | 0.091 | -43.846 | 1.000 |

#### Final selection

With the variables selected above, we generated candidate models of their possible interactions. We ranked them using AICc.

Code

```
OD_models <- list(
OD_m1 <- glmmTMB(n~ Sex,                       family =nbinom2, data = ONLE_D),
OD_m2 <- glmmTMB(n~ Sex+ Wght,                 family =nbinom2, data = ONLE_D),
OD_m3 <- glmmTMB(n~ Sex+ Avg_LC,               family =nbinom2, data = ONLE_D),
OD_m4 <- glmmTMB(n~ Sex+ Rain,               family =nbinom2, data = ONLE_D),
OD_m5 <- glmmTMB(n~ Sex+ Temp,               family =nbinom2, data = ONLE_D),
OD_m6 <- glmmTMB(n~ Rep* Wght,                 family =nbinom2, data = ONLE_D),
OD_m7 <- glmmTMB(n~ Sex* Avg_LC,               family =nbinom2, data = ONLE_D),
OD_m8 <- glmmTMB(n~ Sex* Rain,               family =nbinom2, data = ONLE_D),
OD_m9 <- glmmTMB(n~ Sex* Temp,               family =nbinom2, data = ONLE_D),

OD_m10 <- glmmTMB(n~ Wght,                      family =nbinom2, data = ONLE_D),
OD_m11 <- glmmTMB(n~ Wght+ Avg_LC,              family =nbinom2, data = ONLE_D),
OD_m12 <- glmmTMB(n~ Wght+ Rain,               family =nbinom2, data = ONLE_D),
OD_m13 <- glmmTMB(n~ Wght+ Temp,               family =nbinom2, data = ONLE_D),
OD_m14 <- glmmTMB(n~ Wght* Avg_LC,              family =nbinom2, data = ONLE_D),
OD_m15 <- glmmTMB(n~ Wght* Rain,              family =nbinom2, data = ONLE_D),
OD_m16 <- glmmTMB(n~ Wght* Temp,              family =nbinom2, data = ONLE_D),

OD_m17 <- glmmTMB(n~ Avg_LC,                    family =nbinom2, data = ONLE_D),
OD_m18 <- glmmTMB(n~ Avg_LC+ Rain,            family =nbinom2, data = ONLE_D),
OD_m19 <- glmmTMB(n~ Avg_LC+ Temp,            family =nbinom2, data = ONLE_D),
#OD_m20 <- glmmTMB(n~ Avg_LC* Rain,            family =nbinom2, data = ONLE_D),
OD_m21 <- glmmTMB(n~ Avg_LC* Temp,            family =nbinom2, data = ONLE_D),

OD_m22 <- glmmTMB(n~ Rain,                    family =nbinom2, data = ONLE_D),
OD_m23 <- glmmTMB(n~ Rain+ Temp,                    family =nbinom2, data = ONLE_D),
OD_m24 <- glmmTMB(n~ Rain* Temp,                    family =nbinom2, data = ONLE_D),

OD_m25 <- glmmTMB(n~ Temp,                    family =nbinom2, data = ONLE_D),

OD_m26 <- glmmTMB(n~ 1,                         family =nbinom2, data = ONLE_D),

OD_m27 <- glmmTMB(n~ Month,                         family =nbinom2, data = ONLE_D),
OD_m28 <- glmmTMB(n~ Month*Sex,                         family =nbinom2, data = ONLE_D),
#OD_m29 <- glmmTMB(n~ Month*Wght,                         family =nbinom2, data = ONLE_D),
#OD_m30 <- glmmTMB(n~ Month*Avg_LC,                         family =nbinom2, data = ONLE_D),
#OD_m31 <- glmmTMB(n~ Month*Rain,                         family =nbinom2, data = ONLE_D),
OD_m32 <- glmmTMB(n~ Month*Temp,                         family =nbinom2, data = ONLE_D)
 
)


OD_sel <- aictab(OD_models, sort = F)

OD_sel_table <- get_table_models(OD_models, OD_sel, "Load")


  kbl(OD_sel_table,
    caption = "Final model selection to identify drivers influencing tick load in Onychomys leucogaster",
    digits = 2) %>% 
  kable_styling(bootstrap_options = c("striped", "hover"), full_width = F)
```

Final model selection to identify drivers influencing tick load in Onychomys leucogaster

| Formula | Modnames | K | AICc | Delta\_AICc | ModelLik | AICcWt | LL |
| --- | --- | --- | --- | --- | --- | --- | --- |
| Tick Load ~ Wght \* Avg\_LC | Mod14 | 5 | 82.70 | 0.00 | 1.00 | 0.28 | -36.10 |
| Tick Load ~ Sex + Avg\_LC | Mod3 | 4 | 82.98 | 0.28 | 0.87 | 0.24 | -37.32 |
| Tick Load ~ Avg\_LC + Rain | Mod18 | 4 | 83.76 | 1.06 | 0.59 | 0.17 | -37.71 |
| Tick Load ~ Avg\_LC | Mod17 | 3 | 84.46 | 1.76 | 0.42 | 0.12 | -39.13 |
| Tick Load ~ Sex \* Avg\_LC | Mod7 | 5 | 85.15 | 2.45 | 0.29 | 0.08 | -37.32 |
| Tick Load ~ Avg\_LC + Temp | Mod19 | 4 | 86.57 | 3.87 | 0.14 | 0.04 | -39.12 |
| Tick Load ~ Wght + Avg\_LC | Mod11 | 4 | 86.57 | 3.87 | 0.14 | 0.04 | -39.12 |
| Tick Load ~ Avg\_LC \* Temp | Mod20 | 5 | 88.65 | 5.95 | 0.05 | 0.01 | -39.07 |
| Tick Load ~ Sex | Mod1 | 3 | 92.26 | 9.56 | 0.01 | 0.00 | -43.03 |
| Tick Load ~ Sex + Rain | Mod4 | 4 | 92.40 | 9.69 | 0.01 | 0.00 | -42.03 |
| Tick Load ~ Rain | Mod21 | 3 | 92.60 | 9.90 | 0.01 | 0.00 | -43.20 |
| Tick Load ~ 1 | Mod25 | 2 | 93.05 | 10.35 | 0.01 | 0.00 | -44.48 |
| Tick Load ~ Rain + Temp | Mod22 | 4 | 93.27 | 10.57 | 0.01 | 0.00 | -42.47 |
| Tick Load ~ Wght + Rain | Mod12 | 4 | 93.58 | 10.88 | 0.00 | 0.00 | -42.62 |
| Tick Load ~ Sex + Wght | Mod2 | 4 | 93.76 | 11.06 | 0.00 | 0.00 | -42.71 |
| Tick Load ~ Sex + Temp | Mod5 | 4 | 93.95 | 11.25 | 0.00 | 0.00 | -42.81 |
| Tick Load ~ Wght | Mod10 | 3 | 94.03 | 11.33 | 0.00 | 0.00 | -43.92 |
| Tick Load ~ Temp | Mod24 | 3 | 94.13 | 11.43 | 0.00 | 0.00 | -43.97 |
| Tick Load ~ Sex \* Rain | Mod8 | 5 | 94.57 | 11.87 | 0.00 | 0.00 | -42.03 |
| Tick Load ~ Rain \* Temp | Mod23 | 5 | 95.44 | 12.74 | 0.00 | 0.00 | -42.47 |
| Tick Load ~ Wght + Temp | Mod13 | 4 | 95.48 | 12.78 | 0.00 | 0.00 | -43.57 |
| Tick Load ~ Month | Mod26 | 8 | 95.62 | 12.92 | 0.00 | 0.00 | -39.19 |
| Tick Load ~ Wght \* Rain | Mod15 | 5 | 95.75 | 13.05 | 0.00 | 0.00 | -42.62 |
| Tick Load ~ Sex \* Temp | Mod9 | 5 | 95.92 | 13.22 | 0.00 | 0.00 | -42.70 |
| Tick Load ~ Wght \* Temp | Mod16 | 5 | 97.41 | 14.71 | 0.00 | 0.00 | -43.45 |
| Tick Load ~ Rep \* Wght | Mod6 | 5 | 98.09 | 15.39 | 0.00 | 0.00 | -43.79 |
| Tick Load ~ Month \* Sex | Mod27 | 13 | 102.15 | 19.45 | 0.00 | 0.00 | -36.42 |
| Tick Load ~ Month \* Temp | Mod28 | 13 | 103.69 | 20.99 | 0.00 | 0.00 | -37.19 |

We inspected the 85% confidence intervals of the regression coefficients of the selected models.

Code

```
OD_final_best <- list(OD_m14= OD_m14, OD_m3=OD_m3, OD_m18=OD_m18, OD_m17=OD_m17)

OD_final_best_ci <- map2_df(names(OD_final_best), OD_final_best, get_ci)

 kbl(OD_final_best_ci,
    caption = "Selected models confidence interval table",
    digits = 2) %>% 
  kable_styling(bootstrap_options = c("striped", "hover"), full_width = F)
```

Selected models confidence interval table

| Parameter | Coefficient | CI\_low | CI\_high | Model | Informative |
| --- | --- | --- | --- | --- | --- |
| Wght | 0.27 | 0.08 | 0.47 | OD\_m14 | yes |
| Avg\_LC | 0.47 | 0.23 | 0.72 | OD\_m14 | yes |
| Wght:Avg\_LC | -0.01 | -0.02 | 0.00 | OD\_m14 | yes |
| SexM | -2.00 | -3.60 | -0.39 | OD\_m3 | yes |
| Avg\_LC | 0.11 | 0.06 | 0.16 | OD\_m3 | yes |
| Avg\_LC | 0.11 | 0.06 | 0.16 | OD\_m18 | yes |
| Rain | -720.64 | -301438.74 | 299997.46 | OD\_m18 | no |
| Avg\_LC | 0.11 | 0.06 | 0.16 | OD\_m17 | yes |

Code

```
ci_plot(OD_final_best_ci)+ labs(title= "Onychomys leucogaster models 85%CI")
```

#### Selected models

Subsequently, we inspect the residuals of the models whose confidence intervals do not overlap 0.

Code

```
OD_res <- lapply(list(OD_m14, OD_m3, OD_m17), simulateResiduals, plot= TRUE)
```

Residual inspection of model Tick Load ~ Wght \* Avg\_LC

Residual inspection of model Tick Load ~ Sex + Avg\_LC

Residual inspection of model Tick Load ~ Avg\_LC

We build the summary table of the selected models

Code

```
OD_1_sum <- model_parameters(OD_m14, digits=2, ci= 0.85) %>% 
  mutate(Family= "Negative Binomial") %>% 
  select(-SE, -z, -df_error)

OD_2_sum <- model_parameters(OD_m3, digits=2, ci= 0.85) %>% 
  mutate(Family= "Negative Binomial") %>% 
  select(-SE, -z, -df_error)

OD_3_sum <- model_parameters(OD_m17, digits=2, ci= 0.85) %>% 
  mutate(Family= "Negative Binomial") %>% 
  select(-SE, -z, -df_error)

kbl(rbind(OD_1_sum, OD_2_sum, OD_3_sum),
    caption = "O. leocogaster - Dermacentor",
    digits = 3) %>% 
  kable_styling(bootstrap_options = c("striped", "hover"), full_width = F)
```

O. leocogaster - Dermacentor

| Parameter | Coefficient | CI | CI\_low | CI\_high | p | Component | Effects | Family |
| --- | --- | --- | --- | --- | --- | --- | --- | --- |
| (Intercept) | -15.835 | 0.85 | -24.308 | -7.362 | 0.007 | conditional | fixed | Negative Binomial |
| Wght | 0.274 | 0.85 | 0.082 | 0.466 | 0.040 | conditional | fixed | Negative Binomial |
| Avg\_LC | 0.472 | 0.85 | 0.225 | 0.718 | 0.006 | conditional | fixed | Negative Binomial |
| Wght:Avg\_LC | -0.010 | 0.85 | -0.016 | -0.004 | 0.017 | conditional | fixed | Negative Binomial |
| (Intercept) | 0.225 | 0.85 | 0.095 | 0.529 | NA | dispersion | fixed | Negative Binomial |
| (Intercept) | -4.892 | 0.85 | -6.555 | -3.229 | 0.000 | conditional | fixed | Negative Binomial |
| SexM | -1.997 | 0.85 | -3.604 | -0.391 | 0.073 | conditional | fixed | Negative Binomial |
| Avg\_LC | 0.108 | 0.85 | 0.060 | 0.156 | 0.001 | conditional | fixed | Negative Binomial |
| (Intercept) | 0.179 | 0.85 | 0.077 | 0.414 | NA | dispersion | fixed | Negative Binomial |
| (Intercept) | -5.328 | 0.85 | -7.053 | -3.603 | 0.000 | conditional | fixed | Negative Binomial |
| Avg\_LC | 0.110 | 0.85 | 0.059 | 0.160 | 0.002 | conditional | fixed | Negative Binomial |
| (Intercept) | 0.135 | 0.85 | 0.061 | 0.300 | NA | dispersion | fixed | Negative Binomial |

#### Prediction plots

Code

```
OD_Pred1 <- ggeffect(OD_m14, terms = c( "Wght", "Avg_LC"), ci_level = 0.85)

OD_Pred2 <- ggeffect(OD_m3, terms = "Sex", ci_level = 0.85)

OD_Pred3 <- ggeffect(OD_m3, terms = "Avg_LC", ci_level = 0.85)


OD_pred1_plot <- ggplot()+
  geom_point(data = ONLE_D, aes(x= Wght, y= n))+
  geom_ribbon(data= OD_Pred1, aes(x= x, y=predicted,
                                  ymin=conf.low, ymax=conf.high,
                                  fill=group), alpha= 0.6)+
  geom_line(data= OD_Pred1, aes(x= x, y= predicted, col= group),
            linewidth= 1)+
  labs(x= "Host weitght (g)", y= "Expected tick load",
       fill= "Average litter cover (%)",
       col= "Average litter cover (%)")+
    guides(col= guide_legend(position = "inside"),
         fill= guide_legend(position = "inside"))+
    scale_color_viridis_d()+
    scale_fill_viridis_d()+
    theme_bw(base_size = 12)+
    theme(legend.position.inside = c(0.7, 0.8),
          legend.background = element_blank())

OD_pred2_plot <- catpred_plot(OD_Pred2, title = NULL, 
                               "Sex", ONLE_D, cat=Sex, col= "#A2CD5A")

OD_pred3_plot <- conpred_plot(OD_Pred3, title = NULL, 
                               "Average litter cover (%)",var = Avg_LC, ONLE_D, fill= "#A2CD5A")

OD_pred1_plot
OD_pred2_plot
OD_pred3_plot
```

-“Prediction plot of model Tick Load ~ Wght \* Avg\_LC” -“Prediction plot of model Tick Load ~ Sex + Avg\_LC” -“Prediction plot of model Tick Load ~ Avg\_LC”

-“Prediction plot of model Tick Load ~ Wght \* Avg\_LC” -“Prediction plot of model Tick Load ~ Sex + Avg\_LC” -“Prediction plot of model Tick Load ~ Avg\_LC”

-“Prediction plot of model Tick Load ~ Wght \* Avg\_LC” -“Prediction plot of model Tick Load ~ Sex + Avg\_LC” -“Prediction plot of model Tick Load ~ Avg\_LC”

## ***Peromyscus leucopus***

### ***Dermacentor*** larvae

Code

```
PELE_D <- H_data %>% 
  filter(SM_Sp == "PELE" & 
           Tick_gen %in% c("Dermacentor", "No tick") & 
           Tick_Age %in% c("Larvae", "No tick") )
```

#### Distribution

We selected the most appropriate distribution for the analysis. Because of the count data, we used models that included all variables and used the Poisson, Negative Binomial, and Tweedie distributions. We used the Akaike information criterion for small samples, zero inflated test, and the over dispersion parameter to select the most appropriate distribution.

Code

```
PD_dis <- distri(PELE_D)

kbl(compare_performance(PD_dis, metrics = "AICc"),
    digits = 3) %>% 
  kable_styling(bootstrap_options = c("striped", "hover"), full_width = F)
```

| Name | Model | AICc | AICc\_wt |
| --- | --- | --- | --- |
| PO | glmmTMB | 1425.654 | 0.000 |
| NB | glmmTMB | 872.638 | 0.836 |
| TWD | glmmTMB | 875.898 | 0.164 |

Code

```
testDispersion(PD_dis$PO)
testDispersion(PD_dis$NB)
testDispersion(PD_dis$TWD)
```

-“Dispersion test for Poisson model” -“Dispersion test for Negative Binomial model” -“Dispersion test for Tweedie model”

```
    DHARMa nonparametric dispersion test via sd of residuals fitted vs.
    simulated

data:  simulationOutput
dispersion = 6.973, p-value < 2.2e-16
alternative hypothesis: two.sided
```

-“Dispersion test for Poisson model” -“Dispersion test for Negative Binomial model” -“Dispersion test for Tweedie model”

```
    DHARMa nonparametric dispersion test via sd of residuals fitted vs.
    simulated

data:  simulationOutput
dispersion = 0.49324, p-value = 0.368
alternative hypothesis: two.sided
```

-“Dispersion test for Poisson model” -“Dispersion test for Negative Binomial model” -“Dispersion test for Tweedie model”

```
    DHARMa nonparametric dispersion test via sd of residuals fitted vs.
    simulated

data:  simulationOutput
dispersion = 1.2431, p-value = 0.28
alternative hypothesis: two.sided
```

Code

```
testZeroInflation(PD_dis$PO)
testZeroInflation(PD_dis$NB)
```

-“Zero inflated test for Poisson model” -“Zero inflated for Negative Binomial model”

```
    DHARMa zero-inflation test via comparison to expected zeros with
    simulation under H0 = fitted model

data:  simulationOutput
ratioObsSim = 1.6851, p-value < 2.2e-16
alternative hypothesis: two.sided
```

-“Zero inflated test for Poisson model” -“Zero inflated for Negative Binomial model”

```
    DHARMa zero-inflation test via comparison to expected zeros with
    simulation under H0 = fitted model

data:  simulationOutput
ratioObsSim = 1.016, p-value = 0.68
alternative hypothesis: two.sided
```

Based on the test results, we choose the Binomial Negative family model.

#### Random effects

To evaluate the random effects structure, models with all fixed variables and possible random structures were ranked: Season random intercept, season nested transect, non-nested transect and season, transect and a model with no random effects. Models were ranked using the Akaike information criterion corrected for small samples (AICc).

Code

```
PD_random <- random_eff(PELE_D, nbinom2)
kbl(compare_performance(PD_random, metrics = "AICc"),
    digits = 3) %>% 
  kable_styling(bootstrap_options = c("striped", "hover"), full_width = F)
```

| Name | Model | AICc | AICc\_wt |
| --- | --- | --- | --- |
| Transect | glmmTMB | 874.009 | 0.252 |
| norandom | glmmTMB | 871.827 | 0.748 |

Residual inspection

Code

```
PD_norandom_res <- simulateResiduals(PD_random$norandom, plot = T)
```

Evaluate any spatial correlation issues

Code

```
PD_audf <- get_sau_data(PD_norandom_res, PELE_D)
PD_groups <- split(PD_audf, PD_audf$survey)
PD_corelog <- map(PD_groups, get_correlog) %>% compact()
PD_corelogbase <- map(PD_corelog, get_correlog_base)
PD_corelogplot <- map(PD_corelogbase, get_correlogplot)
PD_corelogplot$Fall_2019
PD_corelogplot$Fall_2020
PD_corelogplot$Spring_2020
PD_corelogplot$Winter_2019
PD_corelogplot$Winter_2020
```

```
500  of  5000 
1000  of  5000 
1500  of  5000 
2000  of  5000 
2500  of  5000 
3000  of  5000 
3500  of  5000 
4000  of  5000 
4500  of  5000 
5000  of  5000 
500  of  5000 
1000  of  5000 
1500  of  5000 
2000  of  5000 
2500  of  5000 
3000  of  5000 
3500  of  5000 
4000  of  5000 
4500  of  5000 
5000  of  5000 
500  of  5000 
1000  of  5000 
1500  of  5000 
2000  of  5000 
2500  of  5000 
3000  of  5000 
3500  of  5000 
4000  of  5000 
4500  of  5000 
5000  of  5000 
500  of  5000 
1000  of  5000 
1500  of  5000 
2000  of  5000 
2500  of  5000 
3000  of  5000 
3500  of  5000 
4000  of  5000 
4500  of  5000 
5000  of  5000 
500  of  5000 
1000  of  5000 
1500  of  5000 
2000  of  5000 
2500  of  5000 
3000  of  5000 
3500  of  5000 
4000  of  5000 
4500  of  5000 
5000  of  5000
```

-“Fall 2019 correlogram” -“Fall 2020 correlogram” -“Spring 2020 correlogram” -“Winter 2019 correlogram” -“Winter 2020 correlogram”

-“Fall 2019 correlogram” -“Fall 2020 correlogram” -“Spring 2020 correlogram” -“Winter 2019 correlogram” -“Winter 2020 correlogram”

-“Fall 2019 correlogram” -“Fall 2020 correlogram” -“Spring 2020 correlogram” -“Winter 2019 correlogram” -“Winter 2020 correlogram”

-“Fall 2019 correlogram” -“Fall 2020 correlogram” -“Spring 2020 correlogram” -“Winter 2019 correlogram” -“Winter 2020 correlogram”

-“Fall 2019 correlogram” -“Fall 2020 correlogram” -“Spring 2020 correlogram” -“Winter 2019 correlogram” -“Winter 2020 correlogram”

According to the inspection of the residuals, the selected model showed adequate goodness-of-fit or spatial autocorrelation problems.

#### Host variables

Univariate models were created for the selection of host variables. The models were ranked using AICc. Variables from models with Delta <2 were used in the final model selection.

Code

```
PD_H <-  get_H_models(PELE_D, family = nbinom2(), zi= FALSE)

PD_H_sel <- aictab(PD_H, sort = T)
kbl(PD_H_sel,
    caption = "Model selection table for host variables",
    digits = 3) %>% 
  kable_styling(bootstrap_options = c("striped", "hover"), full_width = F)
```

Model selection table for host variables

|  | Modnames | K | AICc | Delta\_AICc | ModelLik | AICcWt | LL | Cum.Wt |
| --- | --- | --- | --- | --- | --- | --- | --- | --- |
| 3 | Sex | 3 | 894.125 | 0.000 | 1.000 | 0.669 | -444.026 | 0.669 |
| 4 | Rep | 3 | 896.001 | 1.876 | 0.391 | 0.262 | -444.964 | 0.931 |
| 2 | Weight2 | 4 | 899.129 | 5.004 | 0.082 | 0.055 | -445.503 | 0.986 |
| 1 | Weight | 3 | 901.862 | 7.737 | 0.021 | 0.014 | -447.895 | 1.000 |

#### Habitat variables

Univariate models were created for the selection of habitat variables. The models were ranked using AICc. Variables from models with Delta <2 were used in the final model selection.

Code

```
PD_Ha <- get_Hamodels(PELE_D, family = nbinom2(), zi= FALSE)
PD_Ha_sel <- aictab(PD_Ha, sort = T)

  kbl(PD_Ha_sel,
    caption = "Model selection table for habitat variables",
    digits = 3) %>% 
  kable_styling(bootstrap_options = c("striped", "hover"), full_width = F)
```

Model selection table for habitat variables

|  | Modnames | K | AICc | Delta\_AICc | ModelLik | AICcWt | LL | Cum.Wt |
| --- | --- | --- | --- | --- | --- | --- | --- | --- |
| 6 | Litter\_Cover | 3 | 896.577 | 0.000 | 1.000 | 0.657 | -445.252 | 0.657 |
| 1 | Litter\_depth | 3 | 900.007 | 3.430 | 0.180 | 0.118 | -446.967 | 0.775 |
| 2 | Herbaceus\_Cover | 3 | 900.687 | 4.110 | 0.128 | 0.084 | -447.307 | 0.860 |
| 3 | Canopy\_Cover | 3 | 901.450 | 4.873 | 0.087 | 0.057 | -447.688 | 0.917 |
| 4 | Vegetation\_height | 3 | 901.491 | 4.914 | 0.086 | 0.056 | -447.709 | 0.973 |
| 5 | Vegetation\_height2 | 4 | 902.982 | 6.405 | 0.041 | 0.027 | -447.430 | 1.000 |

#### Weather variables

Univariate models were created for the selection of weather variables. The models were ranked using AICc. Variables from models with Delta <2 were used in the final model selection.

Code

```
PD_W <- get_Wmodels(PELE_D, family = nbinom2(), zi= FALSE)
PD_W_sel <- aictab(PD_W, sort = T)
kbl(PD_W_sel,
    caption = "Model selection table for habitat variables",
    digits = 3) %>% 
  kable_styling(bootstrap_options = c("striped", "hover"), full_width = F)
```

Model selection table for habitat variables

|  | Modnames | K | AICc | Delta\_AICc | ModelLik | AICcWt | LL | Cum.Wt |
| --- | --- | --- | --- | --- | --- | --- | --- | --- |
| 2 | Temp2 | 4 | 880.367 | 0.000 | 1.000 | 0.696 | -436.122 | 0.696 |
| 1 | Temp | 3 | 882.030 | 1.663 | 0.435 | 0.303 | -437.978 | 1.000 |
| 3 | Rain | 3 | 897.044 | 16.676 | 0.000 | 0.000 | -445.485 | 1.000 |
| 4 | Season | 4 | 900.052 | 19.684 | 0.000 | 0.000 | -445.964 | 1.000 |

#### Final selection

With the variables selected above, we generated candidate models of their possible interactions. We ranked them using AICc.

Code

```
PD_models <- list(

PD_m1  <- glmmTMB(n~ Rep,                       family =nbinom2, data = PELE_D),
PD_m2  <- glmmTMB(n~ Rep+ Sex,                  family =nbinom2, data = PELE_D),
PD_m3  <- glmmTMB(n~ Rep+ Avg_LC,               family =nbinom2, data = PELE_D),
PD_m4  <- glmmTMB(n~ Rep+ Temp,                 family =nbinom2, data = PELE_D),
PD_m5  <- glmmTMB(n~ Rep+ Temp+I(Temp^2),       family =nbinom2, data = PELE_D),
PD_m6  <- glmmTMB(n~ Rep* Sex,                  family =nbinom2, data = PELE_D),
PD_m7 <- glmmTMB(n~ Rep* Avg_LC,                family =nbinom2, data = PELE_D),
PD_m8 <- glmmTMB(n~ Rep* Temp,                  family =nbinom2, data = PELE_D),
PD_m9 <- glmmTMB(n~ Rep* (Temp+I(Temp^2)),      family =nbinom2, data = PELE_D),

PD_m10 <- glmmTMB(n~ Sex,                       family =nbinom2, data = PELE_D),
PD_m11 <- glmmTMB(n~ Sex+ Avg_LC,               family =nbinom2, data = PELE_D),
PD_m12 <- glmmTMB(n~ Sex+ Temp,                 family =nbinom2, data = PELE_D),
PD_m13 <- glmmTMB(n~ Sex+ Temp+ I(Temp^2),      family =nbinom2, data = PELE_D),
PD_m14 <- glmmTMB(n~ Sex* Avg_LC,               family =nbinom2, data = PELE_D),
PD_m15 <- glmmTMB(n~ Sex* Temp,                 family =nbinom2, data = PELE_D),
PD_m16 <- glmmTMB(n~ Sex* (Temp+I(Temp^2)),     family =nbinom2, data = PELE_D),

PD_m17 <- glmmTMB(n~ Avg_LC,                    family =nbinom2, data = PELE_D),
PD_m18 <- glmmTMB(n~ Avg_LC+ Temp,              family =nbinom2, data = PELE_D),
PD_m19 <- glmmTMB(n~ Avg_LC+ Temp+ I(Temp^2),   family =nbinom2, data = PELE_D),
PD_m20 <- glmmTMB(n~ Avg_LC* Temp,              family =nbinom2, data = PELE_D),
PD_m21 <- glmmTMB(n~ Avg_LC* (Temp+ I(Temp^2)), family =nbinom2, data = PELE_D),

PD_m22 <- glmmTMB(n~ Temp+ I(Temp^2),           family =nbinom2, data = PELE_D),

PD_m23 <- glmmTMB(n~ 1,                         family =nbinom2, data = PELE_D),

PD_m24 <- glmmTMB(n~ Month,                     family =nbinom2, data = PELE_D),
PD_m25 <- glmmTMB(n~ Month+Sex,                 family =nbinom2, data = PELE_D),
PD_m26 <- glmmTMB(n~ Month+Rep,                 family =nbinom2, data = PELE_D),
PD_m27 <- glmmTMB(n~ Month+Avg_LC,              family =nbinom2, data = PELE_D),
PD_m28 <- glmmTMB(n~ Month+Temp,                family =nbinom2, data = PELE_D),
PD_m29 <- glmmTMB(n~ Month+(Temp+ I(Temp^2)),   family =nbinom2, data = PELE_D),
PD_m30 <- glmmTMB(n~ Month*Sex,                 family =nbinom2, data = PELE_D),
PD_m31 <- glmmTMB(n~ Month*Rep,                 family =nbinom2, data = PELE_D),
PD_m32 <- glmmTMB(n~ Month*Avg_LC,              family =nbinom2, data = PELE_D),
PD_m33 <- glmmTMB(n~ Month*Temp,                family =nbinom2, data = PELE_D),
PD_m34 <- glmmTMB(n~ Month* (Temp+ I(Temp^2)),  family =nbinom2, data = PELE_D)

)


PD_sel <- aictab(PD_models, sort = F)

PD_sel_table <- get_table_models(PD_models, PD_sel, "Load")

  kbl(PD_sel_table,
    caption = "Final model selection to identify drivers influencing tick load in Peromyscus leucopus",
    digits = 2) %>% 
  kable_styling(bootstrap_options = c("striped", "hover"), full_width = F)
```

Final model selection to identify drivers influencing tick load in Peromyscus leucopus

| Formula | Modnames | K | AICc | Delta\_AICc | ModelLik | AICcWt | LL |
| --- | --- | --- | --- | --- | --- | --- | --- |
| Tick Load ~ Month \* Sex | Mod30 | 15 | 839.18 | 0.00 | 1.00 | 0.26 | -403.83 |
| Tick Load ~ Month + Rep | Mod26 | 9 | 839.49 | 0.31 | 0.86 | 0.22 | -410.46 |
| Tick Load ~ Month \* Avg\_LC | Mod32 | 15 | 839.85 | 0.67 | 0.72 | 0.19 | -404.16 |
| Tick Load ~ Month | Mod24 | 8 | 841.64 | 2.46 | 0.29 | 0.08 | -412.60 |
| Tick Load ~ Month + Sex | Mod25 | 9 | 841.79 | 2.61 | 0.27 | 0.07 | -411.61 |
| Tick Load ~ Month + Avg\_LC | Mod27 | 9 | 842.23 | 3.05 | 0.22 | 0.06 | -411.83 |
| Tick Load ~ Month \* Rep | Mod31 | 15 | 843.05 | 3.87 | 0.14 | 0.04 | -405.76 |
| Tick Load ~ Month + Temp | Mod28 | 9 | 843.39 | 4.21 | 0.12 | 0.03 | -412.41 |
| Tick Load ~ Month \* Temp | Mod33 | 15 | 843.47 | 4.29 | 0.12 | 0.03 | -405.97 |
| Tick Load ~ Month + (Temp + I(Temp^2)) | Mod29 | 10 | 844.35 | 5.17 | 0.08 | 0.02 | -411.83 |
| Tick Load ~ Month \* (Temp + I(Temp^2)) | Mod34 | 22 | 857.43 | 18.25 | 0.00 | 0.00 | -405.07 |
| Tick Load ~ Avg\_LC + Temp + I(Temp^2) | Mod19 | 5 | 869.97 | 30.78 | 0.00 | 0.00 | -429.89 |
| Tick Load ~ Rep \* (Temp + I(Temp^2)) | Mod9 | 7 | 873.22 | 34.04 | 0.00 | 0.00 | -429.44 |
| Tick Load ~ Avg\_LC \* (Temp + I(Temp^2)) | Mod21 | 7 | 873.55 | 34.37 | 0.00 | 0.00 | -429.60 |
| Tick Load ~ Avg\_LC + Temp | Mod18 | 4 | 873.67 | 34.48 | 0.00 | 0.00 | -432.77 |
| Tick Load ~ Sex \* (Temp + I(Temp^2)) | Mod16 | 7 | 875.00 | 35.82 | 0.00 | 0.00 | -430.33 |
| Tick Load ~ Avg\_LC \* Temp | Mod20 | 5 | 875.72 | 36.54 | 0.00 | 0.00 | -432.77 |
| Tick Load ~ Rep \* Temp | Mod8 | 5 | 877.03 | 37.85 | 0.00 | 0.00 | -433.42 |
| Tick Load ~ Sex \* Temp | Mod15 | 5 | 877.51 | 38.33 | 0.00 | 0.00 | -433.67 |
| Tick Load ~ Sex + Temp | Mod12 | 4 | 877.85 | 38.67 | 0.00 | 0.00 | -434.86 |
| Tick Load ~ Sex + Temp + I(Temp^2) | Mod13 | 5 | 877.96 | 38.78 | 0.00 | 0.00 | -433.89 |
| Tick Load ~ Rep + Temp + I(Temp^2) | Mod5 | 5 | 878.72 | 39.54 | 0.00 | 0.00 | -434.27 |
| Tick Load ~ Rep + Temp | Mod4 | 4 | 878.79 | 39.61 | 0.00 | 0.00 | -435.33 |
| Tick Load ~ Temp + I(Temp^2) | Mod22 | 4 | 880.37 | 41.18 | 0.00 | 0.00 | -436.12 |
| Tick Load ~ Rep \* Avg\_LC | Mod7 | 5 | 889.38 | 50.19 | 0.00 | 0.00 | -439.60 |
| Tick Load ~ Rep + Avg\_LC | Mod3 | 4 | 890.70 | 51.52 | 0.00 | 0.00 | -441.29 |
| Tick Load ~ Sex + Avg\_LC | Mod11 | 4 | 891.25 | 52.07 | 0.00 | 0.00 | -441.56 |
| Tick Load ~ Rep \* Sex | Mod6 | 5 | 892.71 | 53.53 | 0.00 | 0.00 | -441.26 |
| Tick Load ~ Sex \* Avg\_LC | Mod14 | 5 | 892.77 | 53.59 | 0.00 | 0.00 | -441.29 |
| Tick Load ~ Sex | Mod10 | 3 | 894.13 | 54.94 | 0.00 | 0.00 | -444.03 |
| Tick Load ~ Rep + Sex | Mod2 | 4 | 894.26 | 55.07 | 0.00 | 0.00 | -443.07 |
| Tick Load ~ Rep | Mod1 | 3 | 896.00 | 56.82 | 0.00 | 0.00 | -444.96 |
| Tick Load ~ Avg\_LC | Mod17 | 3 | 896.58 | 57.39 | 0.00 | 0.00 | -445.25 |
| Tick Load ~ 1 | Mod23 | 2 | 900.39 | 61.21 | 0.00 | 0.00 | -448.18 |

We inspected the 85% confidence intervals of the regression coefficients of the selected models.

Code

```
PD_final_best <- list(PD_m30=PD_m30, PD_m26=PD_m26, PD_m32=PD_m32)

PD_final_best_ci <- map2_df(names(PD_final_best), PD_final_best, get_ci)

 kbl(PD_final_best_ci,
    caption = "Selected models confidence interval table",
    digits = 2) %>% 
  kable_styling(bootstrap_options = c("striped", "hover"), full_width = F)
```

Selected models confidence interval table

| Parameter | Coefficient | CI\_low | CI\_high | Model | Informative |
| --- | --- | --- | --- | --- | --- |
| Month3 | -0.80 | -2.15 | 0.55 | PD\_m30 | no |
| Month5 | -1.33 | -2.42 | -0.25 | PD\_m30 | yes |
| Month9 | -20.38 | -6632.92 | 6592.17 | PD\_m30 | no |
| Month10 | -1.61 | -2.56 | -0.67 | PD\_m30 | yes |
| Month11 | -0.80 | -1.72 | 0.12 | PD\_m30 | no |
| Month12 | -1.31 | -2.57 | -0.05 | PD\_m30 | yes |
| SexM | 0.49 | -0.46 | 1.44 | PD\_m30 | no |
| Month3:SexM | 0.16 | -1.42 | 1.74 | PD\_m30 | no |
| Month5:SexM | -0.59 | -2.07 | 0.90 | PD\_m30 | no |
| Month9:SexM | 15.79 | -6596.76 | 6628.34 | PD\_m30 | no |
| Month10:SexM | -2.10 | -3.47 | -0.72 | PD\_m30 | yes |
| Month11:SexM | 0.81 | -0.33 | 1.95 | PD\_m30 | no |
| Month12:SexM | 0.88 | -0.73 | 2.49 | PD\_m30 | no |
| Month3 | -0.63 | -1.35 | 0.09 | PD\_m26 | no |
| Month5 | -1.52 | -2.29 | -0.76 | PD\_m26 | yes |
| Month9 | -5.03 | -6.61 | -3.45 | PD\_m26 | yes |
| Month10 | -2.53 | -3.18 | -1.88 | PD\_m26 | yes |
| Month11 | -0.31 | -0.88 | 0.26 | PD\_m26 | no |
| Month12 | -0.73 | -1.53 | 0.06 | PD\_m26 | no |
| RepY | 0.61 | 0.20 | 1.02 | PD\_m26 | yes |
| Month3 | -0.40 | -4.04 | 3.24 | PD\_m32 | no |
| Month5 | -2.51 | -6.73 | 1.71 | PD\_m32 | no |
| Month9 | -6.05 | -10.81 | -1.29 | PD\_m32 | yes |
| Month10 | -5.09 | -8.48 | -1.70 | PD\_m32 | yes |
| Month11 | -3.19 | -6.38 | 0.00 | PD\_m32 | yes |
| Month12 | -139.70 | -32796.72 | 32517.33 | PD\_m32 | no |
| Avg\_LC | -0.03 | -0.10 | 0.03 | PD\_m32 | no |
| Month3:Avg\_LC | -0.01 | -0.08 | 0.06 | PD\_m32 | no |
| Month5:Avg\_LC | 0.02 | -0.07 | 0.10 | PD\_m32 | no |
| Month9:Avg\_LC | 0.01 | -0.17 | 0.19 | PD\_m32 | no |
| Month10:Avg\_LC | 0.07 | -0.01 | 0.15 | PD\_m32 | no |
| Month11:Avg\_LC | 0.08 | 0.02 | 0.15 | PD\_m32 | yes |
| Month12:Avg\_LC | 6.17 | -1449.18 | 1461.52 | PD\_m32 | no |

Code

```
ci_plot(PD_final_best_ci)+ labs(title= "Peromyscus leucopus models 85%CI")
```

#### Selected models

Subsequently, we inspect the residuals of the models whose confidence intervals do not overlap 0.

Code

```
PD_res <- lapply(list(PD_m30,PD_m26, PD_m32), simulateResiduals, plot= TRUE)
```

Residual inspection of model Tick Load ~ Month \* Sex

Residual inspection of model Tick Load ~ Month + Rep

Residual inspection of model Tick Load ~ Month \* Avg\_LC

Model PD\_m32 n ~ Month \* Avg\_LC Showed a deviaton of homogenity deviance

We build the summary table of the selected models

Code

```
PD_1_sum <- model_parameters(PD_m30, digits=2, ci= 0.85) %>% 
  mutate(Family= "Negative Binomial") %>% 
  select(-SE, -z, -df_error)

PD_2_sum <- model_parameters(PD_m26, digits=2, ci= 0.85) %>% 
  mutate(Family= "Negative Binomial") %>% 
  select(-SE, -z, -df_error)

kbl(rbind(PD_1_sum, PD_2_sum),
    caption = "P. leocopus - Dermacentor",
    digits = 3) %>% 
  kable_styling(bootstrap_options = c("striped", "hover"), full_width = F)
```

P. leocopus - Dermacentor

| Parameter | Coefficient | CI | CI\_low | CI\_high | p | Component | Effects | Family |
| --- | --- | --- | --- | --- | --- | --- | --- | --- |
| (Intercept) | 0.802 | 0.85 | 0.040 | 1.565 | 0.130 | conditional | fixed | Negative Binomial |
| Month3 | -0.802 | 0.85 | -2.153 | 0.548 | 0.393 | conditional | fixed | Negative Binomial |
| Month5 | -1.333 | 0.85 | -2.419 | -0.247 | 0.077 | conditional | fixed | Negative Binomial |
| Month9 | -20.375 | 0.85 | -6632.924 | 6592.174 | 0.996 | conditional | fixed | Negative Binomial |
| Month10 | -1.613 | 0.85 | -2.559 | -0.667 | 0.014 | conditional | fixed | Negative Binomial |
| Month11 | -0.802 | 0.85 | -1.722 | 0.117 | 0.209 | conditional | fixed | Negative Binomial |
| Month12 | -1.313 | 0.85 | -2.574 | -0.052 | 0.134 | conditional | fixed | Negative Binomial |
| SexM | 0.489 | 0.85 | -0.465 | 1.442 | 0.461 | conditional | fixed | Negative Binomial |
| Month3:SexM | 0.158 | 0.85 | -1.424 | 1.740 | 0.886 | conditional | fixed | Negative Binomial |
| Month5:SexM | -0.587 | 0.85 | -2.072 | 0.899 | 0.570 | conditional | fixed | Negative Binomial |
| Month9:SexM | 15.788 | 0.85 | -6596.761 | 6628.337 | 0.997 | conditional | fixed | Negative Binomial |
| Month10:SexM | -2.098 | 0.85 | -3.472 | -0.724 | 0.028 | conditional | fixed | Negative Binomial |
| Month11:SexM | 0.807 | 0.85 | -0.331 | 1.946 | 0.307 | conditional | fixed | Negative Binomial |
| Month12:SexM | 0.882 | 0.85 | -0.730 | 2.495 | 0.431 | conditional | fixed | Negative Binomial |
| (Intercept) | 0.313 | 0.85 | 0.251 | 0.389 | NA | dispersion | fixed | Negative Binomial |
| (Intercept) | 0.772 | 0.85 | 0.248 | 1.296 | 0.034 | conditional | fixed | Negative Binomial |
| Month3 | -0.629 | 0.85 | -1.348 | 0.091 | 0.208 | conditional | fixed | Negative Binomial |
| Month5 | -1.523 | 0.85 | -2.285 | -0.760 | 0.004 | conditional | fixed | Negative Binomial |
| Month9 | -5.031 | 0.85 | -6.610 | -3.452 | 0.000 | conditional | fixed | Negative Binomial |
| Month10 | -2.530 | 0.85 | -3.179 | -1.880 | 0.000 | conditional | fixed | Negative Binomial |
| Month11 | -0.308 | 0.85 | -0.880 | 0.265 | 0.439 | conditional | fixed | Negative Binomial |
| Month12 | -0.734 | 0.85 | -1.531 | 0.063 | 0.185 | conditional | fixed | Negative Binomial |
| RepY | 0.608 | 0.85 | 0.196 | 1.020 | 0.034 | conditional | fixed | Negative Binomial |
| (Intercept) | 0.290 | 0.85 | 0.232 | 0.361 | NA | dispersion | fixed | Negative Binomial |

#### Prediction plots

Code

```
PD_Pred1 <- ggeffect(PD_m30, terms = c("Month", "Sex"), ci_level = 0.85)


PD_Pred2 <- ggeffect(PD_m26, terms = c("Month","Rep"), ci_level = 0.85)


PD_pred1_plot <-ggplot()+
  geom_jitter(data=PELE_D, aes(x= Month, y=n, col= Sex),
              alpha= 0.2,
              position = position_dodge(width = 0.5))+
  geom_pointrange(data = PD_Pred1, aes(x=x, y=predicted, 
                                   ymin= conf.low, ymax= conf.high,
                                   col=group), 
                  position = position_dodge(width = 0.5),
                  size=0.5, linewidth=1.2)+
  labs(y= "Expected tick load", 
       x= "Month", col= "Sex category")+
  guides( col=guide_legend(position = "inside"),
         fill= guide_legend(position = "inside"))+
  scale_color_manual(values = c("deepskyblue2", "dodgerblue4" ))+
  scale_fill_manual(values = c("deepskyblue2", "dodgerblue4" ))+
  theme_bw(base_size = 12)+
  theme(legend.position.inside = c(0.8, 0.8),
        legend.background = element_blank())

PD_pred2_plot <-ggplot()+
  geom_jitter(data=PELE_D, aes(x= Month, y=n, col= Rep),
              alpha= 0.2,
              position = position_dodge(width = 0.5))+
  geom_pointrange(data = PD_Pred2, aes(x=x, y=predicted, 
                                   ymin= conf.low, ymax= conf.high,
                                   col=group), 
                  position = position_dodge(width = 0.5),
                  size=0.5, linewidth=1.2)+
  labs(y= "Expected tick load", 
       x= "Month", col= "Reproductive activity")+
  guides( col=guide_legend(position = "inside"),
         fill= guide_legend(position = "inside"))+
  scale_color_manual(values = c("deepskyblue2", "dodgerblue4" ))+
  scale_fill_manual(values = c("deepskyblue2", "dodgerblue4" ))+
  theme_bw(base_size = 12)+
  theme(legend.position.inside = c(0.8, 0.8),
        legend.background = element_blank())

PD_pred1_plot
PD_pred2_plot
```

-“Prediction plot of model Tick Load ~ Month \* Sex” -“Prediction plot of model Tick Load ~ Month + Rep”

-“Prediction plot of model Tick Load ~ Month \* Sex” -“Prediction plot of model Tick Load ~ Month + Rep”

## ***Sigmodon hispidus***

### \***Dermacentor** larvae

Code

```
SIHI_D <- H_data %>% 
  filter(SM_Sp == "SIHI" & 
           Tick_gen %in% c("Dermacentor", "No tick") & 
           Tick_Age %in% c("Larvae", "No tick") )
```

#### Distribution

We selected the most appropriate distribution for the analysis. Because of the count data, we used models that included all variables and used the Poisson, Negative Binomial, and Tweedie distributions. We used the Akaike information criterion for small samples, zero inflated test, and the over dispersion parameter to select the most appropriate distribution.

Code

```
SD_dis <- distri(SIHI_D)


kbl(compare_performance(SD_dis, metrics = "AICc"),
    digits = 3) %>% 
  kable_styling(bootstrap_options = c("striped", "hover"), full_width = F)
```

| Name | Model | AICc | AICc\_wt |
| --- | --- | --- | --- |
| PO | glmmTMB | 363.048 | 0.000 |
| NB | glmmTMB | 250.219 | 0.962 |
| TWD | glmmTMB | 256.657 | 0.038 |

Code

```
testDispersion(SD_dis$PO)
testDispersion(SD_dis$NB)
testDispersion(SD_dis$TWD)
```

-“Dispersion test for Poisson model” -“Dispersion test for Negative Binomial model” -“Dispersion test for Tweedie model”

```
    DHARMa nonparametric dispersion test via sd of residuals fitted vs.
    simulated

data:  simulationOutput
dispersion = 5.978, p-value < 2.2e-16
alternative hypothesis: two.sided
```

-“Dispersion test for Poisson model” -“Dispersion test for Negative Binomial model” -“Dispersion test for Tweedie model”

```
    DHARMa nonparametric dispersion test via sd of residuals fitted vs.
    simulated

data:  simulationOutput
dispersion = 1.3599, p-value = 0.344
alternative hypothesis: two.sided
```

-“Dispersion test for Poisson model” -“Dispersion test for Negative Binomial model” -“Dispersion test for Tweedie model”

```
    DHARMa nonparametric dispersion test via sd of residuals fitted vs.
    simulated

data:  simulationOutput
dispersion = 1.7668, p-value = 0.16
alternative hypothesis: two.sided
```

Code

```
testZeroInflation(SD_dis$PO)
testZeroInflation(SD_dis$NB)
```

-“Zero inflated test for Poisson model” -“Zero inflated test for Negative Binomial model”

```
    DHARMa zero-inflation test via comparison to expected zeros with
    simulation under H0 = fitted model

data:  simulationOutput
ratioObsSim = 1.0426, p-value < 2.2e-16
alternative hypothesis: two.sided
```

-“Zero inflated test for Poisson model” -“Zero inflated test for Negative Binomial model”

```
    DHARMa zero-inflation test via comparison to expected zeros with
    simulation under H0 = fitted model

data:  simulationOutput
ratioObsSim = 1.0004, p-value = 1
alternative hypothesis: two.sided
```

Based on the test results, we choose the Binomial Negative family model.

#### Random effects

To evaluate the random effects structure, models with all fixed variables and possible random structures were ranked: Season random intercept, season nested transect, non-nested transect and season, transect and a model with no random effects. Models were ranked using the Akaike information criterion corrected for small samples (AICc).

Code

```
SD_random <- random_eff(SIHI_D, nbinom2)

kbl(compare_performance(SD_random, metrics = "AICc"),
    digits = 3) %>% 
  kable_styling(bootstrap_options = c("striped", "hover"), full_width = F)
```

| Name | Model | AICc | AICc\_wt |
| --- | --- | --- | --- |
| Transect | glmmTMB | 250.262 | 0.258 |
| norandom | glmmTMB | 248.147 | 0.742 |

Residual inspection

Code

```
SD_norandom_res <- simulateResiduals(SD_random$norandom, plot = T)
```

Evaluate any spatial correlation issues

Code

```
SD_audf <- get_sau_data(SD_norandom_res, SIHI_D)
SD_groups <- split(SD_audf, SD_audf$survey)
SD_corelog <- map(SD_groups, get_correlog) %>% compact()
SD_corelogbase <- map(SD_corelog, get_correlog_base)
SD_corelogplot <- map(SD_corelogbase, get_correlogplot)
SD_corelogplot$Fall_2019
SD_corelogplot$Fall_2020
SD_corelogplot$Spring_2020
SD_corelogplot$Winter_2019
SD_corelogplot$Winter_2020
```

```
500  of  5000 
1000  of  5000 
1500  of  5000 
2000  of  5000 
2500  of  5000 
3000  of  5000 
3500  of  5000 
4000  of  5000 
4500  of  5000 
5000  of  5000 
500  of  5000 
1000  of  5000 
1500  of  5000 
2000  of  5000 
2500  of  5000 
3000  of  5000 
3500  of  5000 
4000  of  5000 
4500  of  5000 
5000  of  5000 
500  of  5000 
1000  of  5000 
1500  of  5000 
2000  of  5000 
2500  of  5000 
3000  of  5000 
3500  of  5000 
4000  of  5000 
4500  of  5000 
5000  of  5000 
500  of  5000 
1000  of  5000 
1500  of  5000 
2000  of  5000 
2500  of  5000 
3000  of  5000 
3500  of  5000 
4000  of  5000 
4500  of  5000 
5000  of  5000 
500  of  5000 
1000  of  5000 
1500  of  5000 
2000  of  5000 
2500  of  5000 
3000  of  5000 
3500  of  5000 
4000  of  5000 
4500  of  5000 
5000  of  5000
```

-“Fall 2019 correlogram” -“Fall 2020 correlogram” -“Spring 2020 correlogram” -“Winter 2019 correlogram” -“Winter 2020 correlogram”

-“Fall 2019 correlogram” -“Fall 2020 correlogram” -“Spring 2020 correlogram” -“Winter 2019 correlogram” -“Winter 2020 correlogram”

-“Fall 2019 correlogram” -“Fall 2020 correlogram” -“Spring 2020 correlogram” -“Winter 2019 correlogram” -“Winter 2020 correlogram”

-“Fall 2019 correlogram” -“Fall 2020 correlogram” -“Spring 2020 correlogram” -“Winter 2019 correlogram” -“Winter 2020 correlogram”

-“Fall 2019 correlogram” -“Fall 2020 correlogram” -“Spring 2020 correlogram” -“Winter 2019 correlogram” -“Winter 2020 correlogram”

According to the inspection of the residuals, the selected model showed adequate goodness-of-fit or spatial autocorrelation problems.

#### Host variables

Univariate models were created for the selection of host variables. The models were ranked using AICc. Variables from models with Delta <2 were used in the final model selection.

Code

```
SD_H <-  get_H_models(SIHI_D, family = nbinom2(), zi= FALSE)

SD_H_sel <-aictab(SD_H, sort = T)

kbl(SD_H_sel,
    caption = "Model selection table for host variables",
    digits = 3) %>% 
  kable_styling(bootstrap_options = c("striped", "hover"), full_width = F)
```

Model selection table for host variables

|  | Modnames | K | AICc | Delta\_AICc | ModelLik | AICcWt | LL | Cum.Wt |
| --- | --- | --- | --- | --- | --- | --- | --- | --- |
| 4 | Rep | 3 | 254.926 | 0.000 | 1.000 | 0.427 | -124.439 | 0.427 |
| 3 | Sex | 3 | 255.796 | 0.869 | 0.647 | 0.277 | -124.874 | 0.704 |
| 1 | Weight | 3 | 256.358 | 1.432 | 0.489 | 0.209 | -125.155 | 0.913 |
| 2 | Weight2 | 4 | 258.100 | 3.174 | 0.205 | 0.087 | -125.010 | 1.000 |

#### Habitat variables

Univariate models were created for the selection of habitat variables. The models were ranked using AICc. Variables from models with Delta <2 were used in the final model selection.

Code

```
SD_Ha <- get_Hamodels(SIHI_D, family = nbinom2(), zi= FALSE)
SD_Ha_sel <- aictab(SD_Ha, sort = T)
  kbl(SD_Ha_sel,
    caption = "Model selection table for habitat variables",
    digits = 3) %>% 
  kable_styling(bootstrap_options = c("striped", "hover"), full_width = F)
```

Model selection table for habitat variables

|  | Modnames | K | AICc | Delta\_AICc | ModelLik | AICcWt | LL | Cum.Wt |
| --- | --- | --- | --- | --- | --- | --- | --- | --- |
| 5 | Vegetation\_height2 | 4 | 243.222 | 0.000 | 1.000 | 0.837 | -117.571 | 0.837 |
| 3 | Canopy\_Cover | 3 | 248.474 | 5.251 | 0.072 | 0.061 | -121.213 | 0.897 |
| 4 | Vegetation\_height | 3 | 248.602 | 5.379 | 0.068 | 0.057 | -121.277 | 0.954 |
| 6 | Litter\_Cover | 3 | 249.161 | 5.938 | 0.051 | 0.043 | -121.556 | 0.997 |
| 1 | Litter\_depth | 3 | 255.381 | 12.159 | 0.002 | 0.002 | -124.667 | 0.999 |
| 2 | Herbaceus\_Cover | 3 | 256.380 | 13.158 | 0.001 | 0.001 | -125.166 | 1.000 |

#### Weather variables

Univariate models were created for the selection of weather variables. The models were ranked using AICc. Variables from models with Delta <2 were used in the final model selection.

Code

```
SD_W <- get_Wmodels(SIHI_D, family = nbinom2(), zi= FALSE)
SD_W_sel <-aictab(SD_W, sort = T)
  kbl(SD_W_sel,
    caption = "Model selection table for  weather variables",
    digits = 3) %>% 
  kable_styling(bootstrap_options = c("striped", "hover"), full_width = F)
```

Model selection table for weather variables

|  | Modnames | K | AICc | Delta\_AICc | ModelLik | AICcWt | LL | Cum.Wt |
| --- | --- | --- | --- | --- | --- | --- | --- | --- |
| 4 | Season | 4 | 249.058 | 0.000 | 1.000 | 0.942 | -120.489 | 0.942 |
| 2 | Temp2 | 4 | 256.515 | 7.456 | 0.024 | 0.023 | -124.218 | 0.965 |
| 1 | Temp | 3 | 256.798 | 7.740 | 0.021 | 0.020 | -125.375 | 0.985 |
| 3 | Rain | 3 | 257.275 | 8.216 | 0.016 | 0.015 | -125.614 | 1.000 |

#### Final selection

With the variables selected above, we generated candidate models of their possible interactions. We ranked them using AICc.

Code

```
SD_models <- list(

SD_m1 <- glmmTMB(n~ Rep,                        family =nbinom2, data = SIHI_D),
SD_m2 <- glmmTMB(n~ Rep+ Sex,                   family =nbinom2, data = SIHI_D),
SD_m3 <- glmmTMB(n~ Rep+ Wght,                  family =nbinom2, data = SIHI_D),
SD_m4 <- glmmTMB(n~ Rep+ Avg_VH+I(Avg_VH^2),    family =nbinom2, data = SIHI_D),
SD_m5 <- glmmTMB(n~ Rep+ Season,                family =nbinom2, data = SIHI_D),
SD_m6 <- glmmTMB(n~ Rep* Sex,                   family =nbinom2, data = SIHI_D),
SD_m7 <- glmmTMB(n~ Rep* Wght,                  family =nbinom2, data = SIHI_D),
SD_m8 <- glmmTMB(n~ Rep* (Avg_VH+I(Avg_VH^2)),  family =nbinom2, data = SIHI_D),
SD_m9 <- glmmTMB(n~ Rep* Season,                family =nbinom2, data = SIHI_D),


SD_m10 <- glmmTMB(n~ Sex,                       family =nbinom2, data = SIHI_D),
SD_m11 <- glmmTMB(n~ Sex+ Wght,                 family =nbinom2, data = SIHI_D),
SD_m12 <- glmmTMB(n~ Sex+ Avg_VH+I(Avg_VH^2),   family =nbinom2, data = SIHI_D),
SD_m13 <- glmmTMB(n~ Sex+ Season,               family =nbinom2, data = SIHI_D),
SD_m14 <- glmmTMB(n~ Sex* Wght,                 family =nbinom2, data = SIHI_D),
SD_m15 <- glmmTMB(n~ Sex* (Avg_VH+I(Avg_VH^2)), family =nbinom2, data = SIHI_D),
SD_m16 <- glmmTMB(n~ Sex* Season,               family =nbinom2, data = SIHI_D),

SD_m17 <- glmmTMB(n~ Avg_VH+I(Avg_VH^2),          family =nbinom2, data = SIHI_D),
SD_m18 <- glmmTMB(n~ Avg_VH+I(Avg_VH^2)+ Season,  family =nbinom2, data = SIHI_D),

SD_m19 <- glmmTMB(n~ Season,                      family =nbinom2, data = SIHI_D),

SD_m20 <- glmmTMB(n~ 1,                           family =nbinom2, data = SIHI_D),

SD_m21 <- glmmTMB(n~ Month,                       family =nbinom2, data = SIHI_D),
SD_m22 <- glmmTMB(n~ Month+Sex,                   family =nbinom2, data = SIHI_D),
SD_m23 <- glmmTMB(n~ Month+Rep,                   family =nbinom2, data = SIHI_D),
SD_m24 <- glmmTMB(n~ Month+(Avg_VH+I(Avg_VH^2)),  family =nbinom2, data = SIHI_D),
SD_m25 <- glmmTMB(n~ Month*Rep,                   family =nbinom2, data = SIHI_D)


)


SD_sel <- aictab(SD_models, sort = F)
SD_sel_table <- get_table_models(SD_models, SD_sel, "Load")


  kbl(SD_sel_table,
    caption = "Final model selection to identify drivers influencing tick Load in Sigmodon hispidus",
    digits = 2) %>% 
  kable_styling(bootstrap_options = c("striped", "hover"), full_width = F)
```

Final model selection to identify drivers influencing tick Load in Sigmodon hispidus

| Formula | Modnames | K | AICc | Delta\_AICc | ModelLik | AICcWt | LL |
| --- | --- | --- | --- | --- | --- | --- | --- |
| Tick Load ~ Month + (Avg\_VH + I(Avg\_VH^2)) | Mod24 | 10 | 235.45 | 0.00 | 1.00 | 0.35 | -107.50 |
| Tick Load ~ Month | Mod21 | 8 | 236.16 | 0.71 | 0.70 | 0.25 | -109.93 |
| Tick Load ~ Month + Rep | Mod23 | 9 | 236.30 | 0.85 | 0.65 | 0.23 | -108.97 |
| Tick Load ~ Month + Sex | Mod22 | 9 | 238.18 | 2.73 | 0.25 | 0.09 | -109.91 |
| Tick Load ~ Rep \* (Avg\_VH + I(Avg\_VH^2)) | Mod8 | 7 | 239.57 | 4.12 | 0.13 | 0.04 | -112.67 |
| Tick Load ~ Avg\_VH + I(Avg\_VH^2) + Season | Mod18 | 6 | 242.93 | 7.48 | 0.02 | 0.01 | -115.38 |
| Tick Load ~ Rep + Avg\_VH + I(Avg\_VH^2) | Mod4 | 5 | 243.13 | 7.68 | 0.02 | 0.01 | -116.50 |
| Tick Load ~ Avg\_VH + I(Avg\_VH^2) | Mod17 | 4 | 243.22 | 7.78 | 0.02 | 0.01 | -117.57 |
| Tick Load ~ Month \* Rep | Mod25 | 14 | 243.88 | 8.44 | 0.01 | 0.01 | -107.51 |
| Tick Load ~ Sex + Avg\_VH + I(Avg\_VH^2) | Mod12 | 5 | 245.15 | 9.71 | 0.01 | 0.00 | -117.52 |
| Tick Load ~ Sex \* (Avg\_VH + I(Avg\_VH^2)) | Mod15 | 7 | 246.54 | 11.10 | 0.00 | 0.00 | -116.16 |
| Tick Load ~ Season | Mod19 | 4 | 249.06 | 13.61 | 0.00 | 0.00 | -120.49 |
| Tick Load ~ Rep + Season | Mod5 | 5 | 249.65 | 14.21 | 0.00 | 0.00 | -119.77 |
| Tick Load ~ Sex + Season | Mod13 | 5 | 250.98 | 15.54 | 0.00 | 0.00 | -120.43 |
| Tick Load ~ Rep \* Season | Mod9 | 7 | 252.14 | 16.69 | 0.00 | 0.00 | -118.96 |
| Tick Load ~ Sex \* Season | Mod16 | 7 | 252.44 | 17.00 | 0.00 | 0.00 | -119.11 |
| Tick Load ~ Rep + Sex | Mod2 | 4 | 254.70 | 19.25 | 0.00 | 0.00 | -123.31 |
| Tick Load ~ Rep | Mod1 | 3 | 254.93 | 19.48 | 0.00 | 0.00 | -124.44 |
| Tick Load ~ 1 | Mod20 | 2 | 255.51 | 20.07 | 0.00 | 0.00 | -125.74 |
| Tick Load ~ Sex | Mod10 | 3 | 255.80 | 20.35 | 0.00 | 0.00 | -124.87 |
| Tick Load ~ Rep \* Sex | Mod6 | 5 | 255.97 | 20.53 | 0.00 | 0.00 | -122.93 |
| Tick Load ~ Sex + Wght | Mod11 | 4 | 256.64 | 21.19 | 0.00 | 0.00 | -124.28 |
| Tick Load ~ Rep + Wght | Mod3 | 4 | 256.67 | 21.22 | 0.00 | 0.00 | -124.29 |
| Tick Load ~ Rep \* Wght | Mod7 | 5 | 258.21 | 22.77 | 0.00 | 0.00 | -124.05 |
| Tick Load ~ Sex \* Wght | Mod14 | 5 | 258.67 | 23.22 | 0.00 | 0.00 | -124.27 |

We inspected the 85% confidence intervals of the regression coefficients of the selected models.

Code

```
SD_final_best <- list(SD_m24=SD_m24, SD_m21=SD_m21, SD_m23=SD_m23)

SD_final_best_ci <- map2_df(names(SD_final_best), SD_final_best, get_ci)

 kbl(SD_final_best_ci,
    caption = "Selected models confidence interval table",
    digits = 2) %>% 
  kable_styling(bootstrap_options = c("striped", "hover"), full_width = F)
```

Selected models confidence interval table

| Parameter | Coefficient | CI\_low | CI\_high | Model | Informative |
| --- | --- | --- | --- | --- | --- |
| Month3 | -17.58 | NaN | NaN | SD\_m24 | no |
| Month5 | 0.78 | NaN | NaN | SD\_m24 | no |
| Month9 | 5.06 | NaN | NaN | SD\_m24 | no |
| Month10 | 5.31 | NaN | NaN | SD\_m24 | no |
| Month11 | 7.80 | NaN | NaN | SD\_m24 | no |
| Month12 | -10.04 | NaN | NaN | SD\_m24 | no |
| Avg\_VH | -0.14 | NaN | NaN | SD\_m24 | no |
| I(Avg\_VH^2) | 0.00 | NaN | NaN | SD\_m24 | no |
| Month3 | -21.85 | -39958.96 | 39915.26 | SD\_m21 | no |
| Month5 | 0.99 | -1.58 | 3.56 | SD\_m21 | no |
| Month9 | -4.60 | -6.99 | -2.20 | SD\_m21 | yes |
| Month10 | -3.97 | -6.03 | -1.91 | SD\_m21 | yes |
| Month11 | -1.94 | -3.84 | -0.04 | SD\_m21 | yes |
| Month12 | -21.80 | -12870.01 | 12826.40 | SD\_m21 | no |
| Month3 | -22.74 | -61226.50 | 61181.03 | SD\_m23 | no |
| Month5 | 0.97 | -1.52 | 3.46 | SD\_m23 | no |
| Month9 | -4.51 | -6.87 | -2.15 | SD\_m23 | yes |
| Month10 | -3.87 | -5.89 | -1.85 | SD\_m23 | yes |
| Month11 | -1.80 | -3.65 | 0.06 | SD\_m23 | no |
| Month12 | -21.98 | -14802.18 | 14758.22 | SD\_m23 | no |
| RepY | -1.16 | -2.38 | 0.06 | SD\_m23 | no |

Code

```
ci_plot(SD_final_best_ci)+ labs(title= "Sigmodon hispidus models 85%CI")
```

All models had fairly wide confidence intervals O were not possible to estimate in the SD\_m24 model.In all cases it was not possible to obtain a reliable estimate of the regression coefficients.

#### Selected models

Subsequently, we inspect the residuals of the models whose confidence intervals do not overlap 0.

Code

```
SD_res <- lapply(list(SD_m24, SD_m21, SD_m23), simulateResiduals, plot= TRUE)
```

Residual inspection of model Tick Load ~ Month + (Avg\_VH + I(Avg\_VH^2))

Residual inspection of model Tick Load ~ Month

Residual inspection of model Tick Load ~ Month + Rep

The goodness-of-fit tests show a good fit of the models except for SD\_m21, which contains only the month as an explanatory variable.

#### Prediction plots

Code

```
SD_Pred1 <- ggeffect(SD_m24, terms = c("Avg_VH", "Month"), ci_level = 0.85)

SD_Pred2 <- ggeffect(SD_m23, terms = c("Month","Rep"), ci_level = 0.85)

SD_pred1_plot <- ggplot()+
  geom_point(data = SIHI_D, aes(x= Avg_VH, y= n))+
  geom_ribbon(data= SD_Pred1, aes(x= x, y=predicted,
                                  ymin=conf.low, ymax=conf.high,
                                  fill=group), alpha= 0.6)+
  geom_line(data= SD_Pred1, aes(x= x, y= predicted, col= group),
            linewidth= 1)+
  labs(x= "Average vegetation height", y= "Expected tick load",
       fill= "Month",
       col= "Month")+
    guides(col= guide_legend(position = "inside"),
         fill= guide_legend(position = "inside"))+
    scale_color_viridis_d()+
    scale_fill_viridis_d()+
    theme_bw(base_size = 12)+
    theme(legend.position.inside = c(0.7, 0.8),
          legend.background = element_blank())


SD_pred2_plot <-ggplot()+
  geom_jitter(data=SIHI_D, aes(x= Month, y=n, col= Rep),
              alpha= 0.2,
              position = position_dodge(width = 0.5))+
  geom_pointrange(data = SD_Pred2, aes(x=x, y=predicted, 
                                   ymin= conf.low, ymax= conf.high,
                                   col=group), 
                  position = position_dodge(width = 0.5),
                  size=0.5, linewidth=1.2)+
  labs(y= "Expected tick load", 
       x= "Month", col= "Reproductive status")+
  guides( col=guide_legend(position = "inside"),
         fill= guide_legend(position = "inside"))+
  scale_color_manual(values = c("deepskyblue2", "dodgerblue4" ))+
  scale_fill_manual(values = c("deepskyblue2", "dodgerblue4" ))+
  theme_bw(base_size = 12)+
  theme(legend.position.inside = c(0.8, 0.8),
        legend.background = element_blank())


SD_pred1_plot 
SD_pred2_plot
```

-“Prediction plot of model Tick Load ~ Month + (Avg\_VH + I(Avg\_VH^2))” -“Prediction plot of model Tick Load ~ Month + Rep”

-“Prediction plot of model Tick Load ~ Month + (Avg\_VH + I(Avg\_VH^2))” -“Prediction plot of model Tick Load ~ Month + Rep”

# Final Plots

Code

```
(predplots <- (OD_pred1_plot+OD_pred2_plot+ 
  PD_pred1_plot+PD_pred2_plot)+
  plot_layout(ncol = 2, axes = "collect")+
   plot_annotation(tag_levels = 'A'))
```

Code

```
ggsave(plot = predplots, filename = "Figs/Load_predplots.png",
       width=9, height=9)

ggsave(plot = predplots, filename = "Figs/Load_predplots.svg",
       width=9, height=9)
```


##### Source Code

```
---
title: "Statistical modeling process for the analysis of the load of Dermacentor larvae in two species of small rodents."
format:
  html:
   message: false
   warning: false
   code-fold: true
   code-tools: true
   toc: true
   toc-depth: 3
   self-contained: true
   theme: journal
---

# Packages

List of packages used

```{r}
library(tidyverse)
library(glmmTMB)
library(performance)
library(modelbased)
library(DHARMa)
library(patchwork)
library(parameters)
library(AICcmodavg)
library(kableExtra)
library(ggeffects)
```

# Functions

Load custom functions for data organization, analysis and creation of graphs.

```{r}
source("Code/Functions_host.R")

```

# Host level data

Load the main data table

```{r}
T_coords <- read.csv("Data/Tcoords.csv") # coordinate table

#Main data table
H_data <- read.csv("Data/host_level.csv") %>%
  select(-X, -Count, -ID, -Trap, -Trapline, -Date ) %>% 
  #Select only the usable rodents
  filter(SM_Sp %in% c("ONLE", "PELE", "SIHI")) %>% 
  #Drop table mising values
  drop_na() %>%
  #Create year as factor
  mutate(year= as.factor(year),
         Month= as.factor(Month)) %>% 
  #Join with coordinates
  left_join(T_coords) %>% 
  #Create transect ID
  mutate(T_group= as.factor(gsub("[^0-9]", "", Transect) ))


```

# Description of variables

Description of the used variables

```{r}
#| echo: false
library(readxl)
Table_variables <- read_excel("Table variables.xlsx")

kbl(Table_variables,
    caption = "Description of the variables used to model tick presence and load on small mammal hosts in South Texas, USA.",
    digits = 3) %>% 
  kable_styling(bootstrap_options = c("striped", "hover"), full_width = F)

```

# Modeling

## ***O. leucogaster***

### ***Dermacentor*** **larvae**

```{r}
ONLE_D <- H_data %>% 
  filter(SM_Sp == "ONLE" & 
           Tick_gen %in% c("Dermacentor", "No tick") & 
           Tick_Age %in% c("Larvae", "No tick") )
```

#### Distribution

We selected the most appropriate distribution for the analysis. Because of the count data, we used models that included all variables and used the Poisson, Negative Binomial, and Tweedie distributions. We used the Akaike information criterion for small samples, zero inflated test, and the over dispersion parameter to select the most appropriate distribution.

```{r}
OD_dis <- distri(ONLE_D)
kbl(compare_performance(OD_dis, metrics = "AICc"), digits = 3) %>% 
    kable_styling(bootstrap_options = c("striped", "hover"), full_width = F)
  
```

```{r}
#| layout-ncol: 2
#| fig-cap: 
#|   -"Dispersion test for Poisson model"
#|   -"Dispersion test for Negative Binomial model"
#|   -"Dispersion test for Tweedie model"

testDispersion(OD_dis$PO)
testDispersion(OD_dis$NB)
testDispersion(OD_dis$TWD)

```


```{r}
#| layout-ncol: 2
#| fig-cap: 
#|   -"Zero inflation test for Poisson model"
#|   -"Zero inflation test  for Negative Binomial model"
#|  
testZeroInflation(OD_dis$PO)
testZeroInflation(OD_dis$NB)
```

Based on the test results, we choose  the Binomial Negative family model.

#### Random effects

To evaluate the random effects structure, models with all fixed variables and possible random structures were ranked: transect ID random effect and a model with no random effects. Models were ranked using the Akaike information criterion corrected for small samples (AICc).

```{r}
OD_random <-random_eff(ONLE_D, nbinom2())

kbl(compare_performance(OD_random, metrics = "AICc"), digits = 3) %>% 
    kable_styling(bootstrap_options = c("striped", "hover"), full_width = F)

```


Residual inspection

```{r}
OD_norandom_res <- simulateResiduals(OD_random$norandom, plot = T)
```

Evaluate any spatial correlation issues

```{r}
#| layout-ncol: 2
#| fig-cap: 
#|   -"Fall 2019 correlogram"
#|   -"Fall 2020 correlogram"
#|   -"Winter 2019 correlogram"
#|   -"Winter 2020 correlogram"
OD_audf <- get_sau_data(OD_norandom_res, ONLE_D)
OD_groups <- split(OD_audf, OD_audf$survey)
OD_corelog <- map(OD_groups, get_correlog)%>% compact()
OD_corelogbase <- map(OD_corelog, get_correlog_base)
OD_corelogplot <- map(OD_corelogbase, get_correlogplot)
OD_corelogplot$Fall_2019
OD_corelogplot$Fall_2020
OD_corelogplot$Winter_2019
OD_corelogplot$Winter_2020
```

According to the inspection of the residuals, the selected model showed adequate goodness-of-fit or spatial autocorrelation problems.

#### Host variables

Univariate models were created for the selection of host variables. The models were ranked using AICc. Variables from models with Delta <2 were used in the final model selection.

```{r}
OD_H <- get_H_models(ONLE_D, family =nbinom2(),zi = FALSE)
  
OD_H_sel <-aictab(OD_H, sort = T)

kbl(OD_H_sel,
    caption = "Model selection table for host variables",
    digits = 3) %>% 
  kable_styling(bootstrap_options = c("striped", "hover"), full_width = F)

```

#### Habitat variables

Univariate models were created for the selection of habitat variables. The models were ranked using AICc. Variables from models with Delta <2 were used in the final model selection.

```{r}
OD_Ha <- get_Hamodels(ONLE_D, family = nbinom2(),zi = FALSE)
OD_Ha_sel <- aictab(OD_Ha, sort = T)

kbl(OD_Ha_sel,
    caption = "Model selection table for habitat variables",
    digits = 3) %>% 
  kable_styling(bootstrap_options = c("striped", "hover"), full_width = F)
```

#### Weather variables

Univariate models were created for the selection of weather variables. The models were ranked using AICc. Variables from models with Delta <2 were used in the final model selection.

```{r}
OD_W <- get_Wmodels(ONLE_D, family = nbinom2(),zi = FALSE)
OD_W_sel <- aictab(OD_W, sort = T)

kbl(OD_W_sel,
    caption = "Model selection table for  weather variables",
    digits = 3) %>% 
  kable_styling(bootstrap_options = c("striped", "hover"), full_width = F)
```

#### Final selection

With the variables selected above, we generated candidate models of their possible interactions. We ranked them using AICc.

```{r}
OD_models <- list(
OD_m1 <- glmmTMB(n~ Sex,                       family =nbinom2, data = ONLE_D),
OD_m2 <- glmmTMB(n~ Sex+ Wght,                 family =nbinom2, data = ONLE_D),
OD_m3 <- glmmTMB(n~ Sex+ Avg_LC,               family =nbinom2, data = ONLE_D),
OD_m4 <- glmmTMB(n~ Sex+ Rain,               family =nbinom2, data = ONLE_D),
OD_m5 <- glmmTMB(n~ Sex+ Temp,               family =nbinom2, data = ONLE_D),
OD_m6 <- glmmTMB(n~ Rep* Wght,                 family =nbinom2, data = ONLE_D),
OD_m7 <- glmmTMB(n~ Sex* Avg_LC,               family =nbinom2, data = ONLE_D),
OD_m8 <- glmmTMB(n~ Sex* Rain,               family =nbinom2, data = ONLE_D),
OD_m9 <- glmmTMB(n~ Sex* Temp,               family =nbinom2, data = ONLE_D),

OD_m10 <- glmmTMB(n~ Wght,                      family =nbinom2, data = ONLE_D),
OD_m11 <- glmmTMB(n~ Wght+ Avg_LC,              family =nbinom2, data = ONLE_D),
OD_m12 <- glmmTMB(n~ Wght+ Rain,               family =nbinom2, data = ONLE_D),
OD_m13 <- glmmTMB(n~ Wght+ Temp,               family =nbinom2, data = ONLE_D),
OD_m14 <- glmmTMB(n~ Wght* Avg_LC,              family =nbinom2, data = ONLE_D),
OD_m15 <- glmmTMB(n~ Wght* Rain,              family =nbinom2, data = ONLE_D),
OD_m16 <- glmmTMB(n~ Wght* Temp,              family =nbinom2, data = ONLE_D),

OD_m17 <- glmmTMB(n~ Avg_LC,                    family =nbinom2, data = ONLE_D),
OD_m18 <- glmmTMB(n~ Avg_LC+ Rain,            family =nbinom2, data = ONLE_D),
OD_m19 <- glmmTMB(n~ Avg_LC+ Temp,            family =nbinom2, data = ONLE_D),
#OD_m20 <- glmmTMB(n~ Avg_LC* Rain,            family =nbinom2, data = ONLE_D),
OD_m21 <- glmmTMB(n~ Avg_LC* Temp,            family =nbinom2, data = ONLE_D),

OD_m22 <- glmmTMB(n~ Rain,                    family =nbinom2, data = ONLE_D),
OD_m23 <- glmmTMB(n~ Rain+ Temp,                    family =nbinom2, data = ONLE_D),
OD_m24 <- glmmTMB(n~ Rain* Temp,                    family =nbinom2, data = ONLE_D),

OD_m25 <- glmmTMB(n~ Temp,                    family =nbinom2, data = ONLE_D),

OD_m26 <- glmmTMB(n~ 1,                         family =nbinom2, data = ONLE_D),

OD_m27 <- glmmTMB(n~ Month,                         family =nbinom2, data = ONLE_D),
OD_m28 <- glmmTMB(n~ Month*Sex,                         family =nbinom2, data = ONLE_D),
#OD_m29 <- glmmTMB(n~ Month*Wght,                         family =nbinom2, data = ONLE_D),
#OD_m30 <- glmmTMB(n~ Month*Avg_LC,                         family =nbinom2, data = ONLE_D),
#OD_m31 <- glmmTMB(n~ Month*Rain,                         family =nbinom2, data = ONLE_D),
OD_m32 <- glmmTMB(n~ Month*Temp,                         family =nbinom2, data = ONLE_D)
 
)


OD_sel <- aictab(OD_models, sort = F)

OD_sel_table <- get_table_models(OD_models, OD_sel, "Load")


  kbl(OD_sel_table,
    caption = "Final model selection to identify drivers influencing tick load in Onychomys leucogaster",
    digits = 2) %>% 
  kable_styling(bootstrap_options = c("striped", "hover"), full_width = F)
```

We inspected the 85% confidence intervals of the regression coefficients of the selected models.

```{r}
OD_final_best <- list(OD_m14= OD_m14, OD_m3=OD_m3, OD_m18=OD_m18, OD_m17=OD_m17)

OD_final_best_ci <- map2_df(names(OD_final_best), OD_final_best, get_ci)

 kbl(OD_final_best_ci,
    caption = "Selected models confidence interval table",
    digits = 2) %>% 
  kable_styling(bootstrap_options = c("striped", "hover"), full_width = F)


```


```{r}
ci_plot(OD_final_best_ci)+ labs(title= "Onychomys leucogaster models 85%CI")
```

#### Selected models

Subsequently, we inspect the residuals of the models whose confidence intervals do not overlap 0.

```{r}
#| layout-ncol: 2
#| fig-cap: 
#|  - "Residual inspection of model Tick Load ~ Wght * Avg_LC"
#|  - "Residual inspection of model Tick Load ~ Sex + Avg_LC"
#|  - "Residual inspection of model Tick Load ~ Avg_LC"
OD_res <- lapply(list(OD_m14, OD_m3, OD_m17), simulateResiduals, plot= TRUE)

```

We build the summary table of the selected models

```{r}
OD_1_sum <- model_parameters(OD_m14, digits=2, ci= 0.85) %>% 
  mutate(Family= "Negative Binomial") %>% 
  select(-SE, -z, -df_error)

OD_2_sum <- model_parameters(OD_m3, digits=2, ci= 0.85) %>% 
  mutate(Family= "Negative Binomial") %>% 
  select(-SE, -z, -df_error)

OD_3_sum <- model_parameters(OD_m17, digits=2, ci= 0.85) %>% 
  mutate(Family= "Negative Binomial") %>% 
  select(-SE, -z, -df_error)

kbl(rbind(OD_1_sum, OD_2_sum, OD_3_sum),
    caption = "O. leocogaster - Dermacentor",
    digits = 3) %>% 
  kable_styling(bootstrap_options = c("striped", "hover"), full_width = F)
```

#### Prediction plots

```{r}
#| layout-ncol: 2
#| fig-cap: 
#|   -"Prediction plot of model Tick Load ~ Wght * Avg_LC"
#|   -"Prediction plot of model Tick Load ~ Sex + Avg_LC"
#|   -"Prediction plot of model Tick Load ~ Avg_LC"
     
OD_Pred1 <- ggeffect(OD_m14, terms = c( "Wght", "Avg_LC"), ci_level = 0.85)

OD_Pred2 <- ggeffect(OD_m3, terms = "Sex", ci_level = 0.85)

OD_Pred3 <- ggeffect(OD_m3, terms = "Avg_LC", ci_level = 0.85)


OD_pred1_plot <- ggplot()+
  geom_point(data = ONLE_D, aes(x= Wght, y= n))+
  geom_ribbon(data= OD_Pred1, aes(x= x, y=predicted,
                                  ymin=conf.low, ymax=conf.high,
                                  fill=group), alpha= 0.6)+
  geom_line(data= OD_Pred1, aes(x= x, y= predicted, col= group),
            linewidth= 1)+
  labs(x= "Host weitght (g)", y= "Expected tick load",
       fill= "Average litter cover (%)",
       col= "Average litter cover (%)")+
    guides(col= guide_legend(position = "inside"),
         fill= guide_legend(position = "inside"))+
    scale_color_viridis_d()+
    scale_fill_viridis_d()+
    theme_bw(base_size = 12)+
    theme(legend.position.inside = c(0.7, 0.8),
          legend.background = element_blank())

OD_pred2_plot <- catpred_plot(OD_Pred2, title = NULL, 
                               "Sex", ONLE_D, cat=Sex, col= "#A2CD5A")

OD_pred3_plot <- conpred_plot(OD_Pred3, title = NULL, 
                               "Average litter cover (%)",var = Avg_LC, ONLE_D, fill= "#A2CD5A")

OD_pred1_plot
OD_pred2_plot
OD_pred3_plot

```

## ***Peromyscus leucopus***

### ***Dermacentor*** larvae

```{r}
PELE_D <- H_data %>% 
  filter(SM_Sp == "PELE" & 
           Tick_gen %in% c("Dermacentor", "No tick") & 
           Tick_Age %in% c("Larvae", "No tick") )
```

#### Distribution

We selected the most appropriate distribution for the analysis. Because of the count data, we used models that included all variables and used the Poisson, Negative Binomial, and Tweedie distributions. We used the Akaike information criterion for small samples, zero inflated test, and the over dispersion parameter to select the most appropriate distribution.

```{r}
PD_dis <- distri(PELE_D)

kbl(compare_performance(PD_dis, metrics = "AICc"),
    digits = 3) %>% 
  kable_styling(bootstrap_options = c("striped", "hover"), full_width = F)
```

```{r}
#| layout-ncol: 2
#| fig-cap: 
#|   -"Dispersion test for Poisson model"
#|   -"Dispersion test for Negative Binomial model"
#|   -"Dispersion test for Tweedie model"

testDispersion(PD_dis$PO)
testDispersion(PD_dis$NB)
testDispersion(PD_dis$TWD)


```


```{r}
#| layout-ncol: 2
#| fig-cap: 
#|   -"Zero inflated test for Poisson model"
#|   -"Zero inflated for Negative Binomial model"


testZeroInflation(PD_dis$PO)
testZeroInflation(PD_dis$NB)
```

Based on the test results, we choose the Binomial Negative family model.

#### Random effects

To evaluate the random effects structure, models with all fixed variables and possible random structures were ranked: Season random intercept, season nested transect, non-nested transect and season, transect and a model with no random effects. Models were ranked using the Akaike information criterion corrected for small samples (AICc).

```{r}
PD_random <- random_eff(PELE_D, nbinom2)
kbl(compare_performance(PD_random, metrics = "AICc"),
    digits = 3) %>% 
  kable_styling(bootstrap_options = c("striped", "hover"), full_width = F)

```

Residual inspection

```{r}
PD_norandom_res <- simulateResiduals(PD_random$norandom, plot = T)
```

Evaluate any spatial correlation issues

```{r}
#| layout-ncol: 2
#| fig-cap: 
#|   -"Fall 2019 correlogram"
#|   -"Fall 2020 correlogram"
#|   -"Spring 2020 correlogram"
#|   -"Winter 2019 correlogram"
#|   -"Winter 2020 correlogram"
PD_audf <- get_sau_data(PD_norandom_res, PELE_D)
PD_groups <- split(PD_audf, PD_audf$survey)
PD_corelog <- map(PD_groups, get_correlog) %>% compact()
PD_corelogbase <- map(PD_corelog, get_correlog_base)
PD_corelogplot <- map(PD_corelogbase, get_correlogplot)
PD_corelogplot$Fall_2019
PD_corelogplot$Fall_2020
PD_corelogplot$Spring_2020
PD_corelogplot$Winter_2019
PD_corelogplot$Winter_2020

```

According to the inspection of the residuals, the selected model showed adequate goodness-of-fit or spatial autocorrelation problems.

#### Host variables

Univariate models were created for the selection of host variables. The models were ranked using AICc. Variables from models with Delta <2 were used in the final model selection.

```{r}
PD_H <-  get_H_models(PELE_D, family = nbinom2(), zi= FALSE)

PD_H_sel <- aictab(PD_H, sort = T)
kbl(PD_H_sel,
    caption = "Model selection table for host variables",
    digits = 3) %>% 
  kable_styling(bootstrap_options = c("striped", "hover"), full_width = F)

```

#### Habitat variables

Univariate models were created for the selection of habitat variables. The models were ranked using AICc. Variables from models with Delta <2 were used in the final model selection.

```{r}
PD_Ha <- get_Hamodels(PELE_D, family = nbinom2(), zi= FALSE)
PD_Ha_sel <- aictab(PD_Ha, sort = T)

  kbl(PD_Ha_sel,
    caption = "Model selection table for habitat variables",
    digits = 3) %>% 
  kable_styling(bootstrap_options = c("striped", "hover"), full_width = F)
```

#### Weather variables

Univariate models were created for the selection of weather variables. The models were ranked using AICc. Variables from models with Delta <2 were used in the final model selection.

```{r}
PD_W <- get_Wmodels(PELE_D, family = nbinom2(), zi= FALSE)
PD_W_sel <- aictab(PD_W, sort = T)
kbl(PD_W_sel,
    caption = "Model selection table for habitat variables",
    digits = 3) %>% 
  kable_styling(bootstrap_options = c("striped", "hover"), full_width = F)
```

#### Final selection

With the variables selected above, we generated candidate models of their possible interactions. We ranked them using AICc.

```{r}
PD_models <- list(

PD_m1  <- glmmTMB(n~ Rep,                       family =nbinom2, data = PELE_D),
PD_m2  <- glmmTMB(n~ Rep+ Sex,                  family =nbinom2, data = PELE_D),
PD_m3  <- glmmTMB(n~ Rep+ Avg_LC,               family =nbinom2, data = PELE_D),
PD_m4  <- glmmTMB(n~ Rep+ Temp,                 family =nbinom2, data = PELE_D),
PD_m5  <- glmmTMB(n~ Rep+ Temp+I(Temp^2),       family =nbinom2, data = PELE_D),
PD_m6  <- glmmTMB(n~ Rep* Sex,                  family =nbinom2, data = PELE_D),
PD_m7 <- glmmTMB(n~ Rep* Avg_LC,                family =nbinom2, data = PELE_D),
PD_m8 <- glmmTMB(n~ Rep* Temp,                  family =nbinom2, data = PELE_D),
PD_m9 <- glmmTMB(n~ Rep* (Temp+I(Temp^2)),      family =nbinom2, data = PELE_D),

PD_m10 <- glmmTMB(n~ Sex,                       family =nbinom2, data = PELE_D),
PD_m11 <- glmmTMB(n~ Sex+ Avg_LC,               family =nbinom2, data = PELE_D),
PD_m12 <- glmmTMB(n~ Sex+ Temp,                 family =nbinom2, data = PELE_D),
PD_m13 <- glmmTMB(n~ Sex+ Temp+ I(Temp^2),      family =nbinom2, data = PELE_D),
PD_m14 <- glmmTMB(n~ Sex* Avg_LC,               family =nbinom2, data = PELE_D),
PD_m15 <- glmmTMB(n~ Sex* Temp,                 family =nbinom2, data = PELE_D),
PD_m16 <- glmmTMB(n~ Sex* (Temp+I(Temp^2)),     family =nbinom2, data = PELE_D),

PD_m17 <- glmmTMB(n~ Avg_LC,                    family =nbinom2, data = PELE_D),
PD_m18 <- glmmTMB(n~ Avg_LC+ Temp,              family =nbinom2, data = PELE_D),
PD_m19 <- glmmTMB(n~ Avg_LC+ Temp+ I(Temp^2),   family =nbinom2, data = PELE_D),
PD_m20 <- glmmTMB(n~ Avg_LC* Temp,              family =nbinom2, data = PELE_D),
PD_m21 <- glmmTMB(n~ Avg_LC* (Temp+ I(Temp^2)), family =nbinom2, data = PELE_D),

PD_m22 <- glmmTMB(n~ Temp+ I(Temp^2),           family =nbinom2, data = PELE_D),

PD_m23 <- glmmTMB(n~ 1,                         family =nbinom2, data = PELE_D),

PD_m24 <- glmmTMB(n~ Month,                     family =nbinom2, data = PELE_D),
PD_m25 <- glmmTMB(n~ Month+Sex,                 family =nbinom2, data = PELE_D),
PD_m26 <- glmmTMB(n~ Month+Rep,                 family =nbinom2, data = PELE_D),
PD_m27 <- glmmTMB(n~ Month+Avg_LC,              family =nbinom2, data = PELE_D),
PD_m28 <- glmmTMB(n~ Month+Temp,                family =nbinom2, data = PELE_D),
PD_m29 <- glmmTMB(n~ Month+(Temp+ I(Temp^2)),   family =nbinom2, data = PELE_D),
PD_m30 <- glmmTMB(n~ Month*Sex,                 family =nbinom2, data = PELE_D),
PD_m31 <- glmmTMB(n~ Month*Rep,                 family =nbinom2, data = PELE_D),
PD_m32 <- glmmTMB(n~ Month*Avg_LC,              family =nbinom2, data = PELE_D),
PD_m33 <- glmmTMB(n~ Month*Temp,                family =nbinom2, data = PELE_D),
PD_m34 <- glmmTMB(n~ Month* (Temp+ I(Temp^2)),  family =nbinom2, data = PELE_D)

)


PD_sel <- aictab(PD_models, sort = F)

PD_sel_table <- get_table_models(PD_models, PD_sel, "Load")

  kbl(PD_sel_table,
    caption = "Final model selection to identify drivers influencing tick load in Peromyscus leucopus",
    digits = 2) %>% 
  kable_styling(bootstrap_options = c("striped", "hover"), full_width = F)

```

We inspected the 85% confidence intervals of the regression coefficients of the selected models.

```{r}
PD_final_best <- list(PD_m30=PD_m30, PD_m26=PD_m26, PD_m32=PD_m32)

PD_final_best_ci <- map2_df(names(PD_final_best), PD_final_best, get_ci)

 kbl(PD_final_best_ci,
    caption = "Selected models confidence interval table",
    digits = 2) %>% 
  kable_styling(bootstrap_options = c("striped", "hover"), full_width = F)


```

```{r}
ci_plot(PD_final_best_ci)+ labs(title= "Peromyscus leucopus models 85%CI")
```


#### Selected models

Subsequently, we inspect the residuals of the models whose confidence intervals do not overlap 0.

```{r}
#| layout-ncol: 2
#| fig-cap: 
#|  - "Residual inspection of model Tick Load ~ Month * Sex"
#|  - "Residual inspection of model Tick Load ~ Month + Rep"
#|  - "Residual inspection of model Tick Load ~ Month * Avg_LC"
PD_res <- lapply(list(PD_m30,PD_m26, PD_m32), simulateResiduals, plot= TRUE)


```

Model PD_m32  n ~ Month * Avg_LC Showed a deviaton of homogenity deviance

We build the summary table of the selected models

```{r}
PD_1_sum <- model_parameters(PD_m30, digits=2, ci= 0.85) %>% 
  mutate(Family= "Negative Binomial") %>% 
  select(-SE, -z, -df_error)

PD_2_sum <- model_parameters(PD_m26, digits=2, ci= 0.85) %>% 
  mutate(Family= "Negative Binomial") %>% 
  select(-SE, -z, -df_error)

kbl(rbind(PD_1_sum, PD_2_sum),
    caption = "P. leocopus - Dermacentor",
    digits = 3) %>% 
  kable_styling(bootstrap_options = c("striped", "hover"), full_width = F)
```

#### Prediction plots

```{r}
#| layout-ncol: 2
#| fig-cap: 
#|   -"Prediction plot of model Tick Load ~ Month * Sex"
#|   -"Prediction plot of model Tick Load ~ Month + Rep"


PD_Pred1 <- ggeffect(PD_m30, terms = c("Month", "Sex"), ci_level = 0.85)


PD_Pred2 <- ggeffect(PD_m26, terms = c("Month","Rep"), ci_level = 0.85)


PD_pred1_plot <-ggplot()+
  geom_jitter(data=PELE_D, aes(x= Month, y=n, col= Sex),
              alpha= 0.2,
              position = position_dodge(width = 0.5))+
  geom_pointrange(data = PD_Pred1, aes(x=x, y=predicted, 
                                   ymin= conf.low, ymax= conf.high,
                                   col=group), 
                  position = position_dodge(width = 0.5),
                  size=0.5, linewidth=1.2)+
  labs(y= "Expected tick load", 
       x= "Month", col= "Sex category")+
  guides( col=guide_legend(position = "inside"),
         fill= guide_legend(position = "inside"))+
  scale_color_manual(values = c("deepskyblue2", "dodgerblue4" ))+
  scale_fill_manual(values = c("deepskyblue2", "dodgerblue4" ))+
  theme_bw(base_size = 12)+
  theme(legend.position.inside = c(0.8, 0.8),
        legend.background = element_blank())

PD_pred2_plot <-ggplot()+
  geom_jitter(data=PELE_D, aes(x= Month, y=n, col= Rep),
              alpha= 0.2,
              position = position_dodge(width = 0.5))+
  geom_pointrange(data = PD_Pred2, aes(x=x, y=predicted, 
                                   ymin= conf.low, ymax= conf.high,
                                   col=group), 
                  position = position_dodge(width = 0.5),
                  size=0.5, linewidth=1.2)+
  labs(y= "Expected tick load", 
       x= "Month", col= "Reproductive activity")+
  guides( col=guide_legend(position = "inside"),
         fill= guide_legend(position = "inside"))+
  scale_color_manual(values = c("deepskyblue2", "dodgerblue4" ))+
  scale_fill_manual(values = c("deepskyblue2", "dodgerblue4" ))+
  theme_bw(base_size = 12)+
  theme(legend.position.inside = c(0.8, 0.8),
        legend.background = element_blank())

PD_pred1_plot
PD_pred2_plot

```

## ***Sigmodon hispidus***

### ***Dermacentor** larvae

```{r}
SIHI_D <- H_data %>% 
  filter(SM_Sp == "SIHI" & 
           Tick_gen %in% c("Dermacentor", "No tick") & 
           Tick_Age %in% c("Larvae", "No tick") )
```

#### Distribution

We selected the most appropriate distribution for the analysis. Because of the count data, we used models that included all variables and used the Poisson, Negative Binomial, and Tweedie distributions. We used the Akaike information criterion for small samples, zero inflated test, and the over dispersion parameter to select the most appropriate distribution.

```{r}
SD_dis <- distri(SIHI_D)


kbl(compare_performance(SD_dis, metrics = "AICc"),
    digits = 3) %>% 
  kable_styling(bootstrap_options = c("striped", "hover"), full_width = F)

```

```{r}
#| layout-ncol: 2
#| fig-cap: 
#|   -"Dispersion test for Poisson model"
#|   -"Dispersion test for Negative Binomial model"
#|   -"Dispersion test for Tweedie model"
testDispersion(SD_dis$PO)
testDispersion(SD_dis$NB)
testDispersion(SD_dis$TWD)


```


```{r}
#| layout-ncol: 2
#| fig-cap: 
#|   -"Zero inflated test for Poisson model"
#|   -"Zero inflated test for Negative Binomial model"

testZeroInflation(SD_dis$PO)
testZeroInflation(SD_dis$NB)
```


Based on the test results, we choose the Binomial Negative family model.

#### Random effects

To evaluate the random effects structure, models with all fixed variables and possible random structures were ranked: Season random intercept, season nested transect, non-nested transect and season, transect and a model with no random effects. Models were ranked using the Akaike information criterion corrected for small samples (AICc).

```{r}
SD_random <- random_eff(SIHI_D, nbinom2)

kbl(compare_performance(SD_random, metrics = "AICc"),
    digits = 3) %>% 
  kable_styling(bootstrap_options = c("striped", "hover"), full_width = F)

```

Residual inspection

```{r}
SD_norandom_res <- simulateResiduals(SD_random$norandom, plot = T)
```

Evaluate any spatial correlation issues

```{r}
#| layout-ncol: 2
#| fig-cap: 
#|   -"Fall 2019 correlogram"
#|   -"Fall 2020 correlogram"
#|   -"Spring 2020 correlogram"
#|   -"Winter 2019 correlogram"
#|   -"Winter 2020 correlogram"
SD_audf <- get_sau_data(SD_norandom_res, SIHI_D)
SD_groups <- split(SD_audf, SD_audf$survey)
SD_corelog <- map(SD_groups, get_correlog) %>% compact()
SD_corelogbase <- map(SD_corelog, get_correlog_base)
SD_corelogplot <- map(SD_corelogbase, get_correlogplot)
SD_corelogplot$Fall_2019
SD_corelogplot$Fall_2020
SD_corelogplot$Spring_2020
SD_corelogplot$Winter_2019
SD_corelogplot$Winter_2020


```

According to the inspection of the residuals, the selected model showed adequate goodness-of-fit or spatial autocorrelation problems.

#### Host variables

Univariate models were created for the selection of host variables. The models were ranked using AICc. Variables from models with Delta <2 were used in the final model selection.

```{r}
SD_H <-  get_H_models(SIHI_D, family = nbinom2(), zi= FALSE)

SD_H_sel <-aictab(SD_H, sort = T)

kbl(SD_H_sel,
    caption = "Model selection table for host variables",
    digits = 3) %>% 
  kable_styling(bootstrap_options = c("striped", "hover"), full_width = F)

```

#### Habitat variables

Univariate models were created for the selection of habitat variables. The models were ranked using AICc. Variables from models with Delta <2 were used in the final model selection.

```{r}
SD_Ha <- get_Hamodels(SIHI_D, family = nbinom2(), zi= FALSE)
SD_Ha_sel <- aictab(SD_Ha, sort = T)
  kbl(SD_Ha_sel,
    caption = "Model selection table for habitat variables",
    digits = 3) %>% 
  kable_styling(bootstrap_options = c("striped", "hover"), full_width = F)
```

#### Weather variables

Univariate models were created for the selection of weather variables. The models were ranked using AICc. Variables from models with Delta <2 were used in the final model selection.

```{r}
SD_W <- get_Wmodels(SIHI_D, family = nbinom2(), zi= FALSE)
SD_W_sel <-aictab(SD_W, sort = T)
  kbl(SD_W_sel,
    caption = "Model selection table for  weather variables",
    digits = 3) %>% 
  kable_styling(bootstrap_options = c("striped", "hover"), full_width = F)
```

#### Final selection

With the variables selected above, we generated candidate models of their possible interactions. We ranked them using AICc.

```{r}
SD_models <- list(

SD_m1 <- glmmTMB(n~ Rep,                        family =nbinom2, data = SIHI_D),
SD_m2 <- glmmTMB(n~ Rep+ Sex,                   family =nbinom2, data = SIHI_D),
SD_m3 <- glmmTMB(n~ Rep+ Wght,                  family =nbinom2, data = SIHI_D),
SD_m4 <- glmmTMB(n~ Rep+ Avg_VH+I(Avg_VH^2),    family =nbinom2, data = SIHI_D),
SD_m5 <- glmmTMB(n~ Rep+ Season,                family =nbinom2, data = SIHI_D),
SD_m6 <- glmmTMB(n~ Rep* Sex,                   family =nbinom2, data = SIHI_D),
SD_m7 <- glmmTMB(n~ Rep* Wght,                  family =nbinom2, data = SIHI_D),
SD_m8 <- glmmTMB(n~ Rep* (Avg_VH+I(Avg_VH^2)),  family =nbinom2, data = SIHI_D),
SD_m9 <- glmmTMB(n~ Rep* Season,                family =nbinom2, data = SIHI_D),


SD_m10 <- glmmTMB(n~ Sex,                       family =nbinom2, data = SIHI_D),
SD_m11 <- glmmTMB(n~ Sex+ Wght,                 family =nbinom2, data = SIHI_D),
SD_m12 <- glmmTMB(n~ Sex+ Avg_VH+I(Avg_VH^2),   family =nbinom2, data = SIHI_D),
SD_m13 <- glmmTMB(n~ Sex+ Season,               family =nbinom2, data = SIHI_D),
SD_m14 <- glmmTMB(n~ Sex* Wght,                 family =nbinom2, data = SIHI_D),
SD_m15 <- glmmTMB(n~ Sex* (Avg_VH+I(Avg_VH^2)), family =nbinom2, data = SIHI_D),
SD_m16 <- glmmTMB(n~ Sex* Season,               family =nbinom2, data = SIHI_D),

SD_m17 <- glmmTMB(n~ Avg_VH+I(Avg_VH^2),          family =nbinom2, data = SIHI_D),
SD_m18 <- glmmTMB(n~ Avg_VH+I(Avg_VH^2)+ Season,  family =nbinom2, data = SIHI_D),

SD_m19 <- glmmTMB(n~ Season,                      family =nbinom2, data = SIHI_D),

SD_m20 <- glmmTMB(n~ 1,                           family =nbinom2, data = SIHI_D),

SD_m21 <- glmmTMB(n~ Month,                       family =nbinom2, data = SIHI_D),
SD_m22 <- glmmTMB(n~ Month+Sex,                   family =nbinom2, data = SIHI_D),
SD_m23 <- glmmTMB(n~ Month+Rep,                   family =nbinom2, data = SIHI_D),
SD_m24 <- glmmTMB(n~ Month+(Avg_VH+I(Avg_VH^2)),  family =nbinom2, data = SIHI_D),
SD_m25 <- glmmTMB(n~ Month*Rep,                   family =nbinom2, data = SIHI_D)


)


SD_sel <- aictab(SD_models, sort = F)
SD_sel_table <- get_table_models(SD_models, SD_sel, "Load")


  kbl(SD_sel_table,
    caption = "Final model selection to identify drivers influencing tick Load in Sigmodon hispidus",
    digits = 2) %>% 
  kable_styling(bootstrap_options = c("striped", "hover"), full_width = F)

```
We inspected the 85% confidence intervals of the regression coefficients of the selected models.

```{r}
SD_final_best <- list(SD_m24=SD_m24, SD_m21=SD_m21, SD_m23=SD_m23)

SD_final_best_ci <- map2_df(names(SD_final_best), SD_final_best, get_ci)

 kbl(SD_final_best_ci,
    caption = "Selected models confidence interval table",
    digits = 2) %>% 
  kable_styling(bootstrap_options = c("striped", "hover"), full_width = F)


```


```{r}
ci_plot(SD_final_best_ci)+ labs(title= "Sigmodon hispidus models 85%CI")
```


All models had fairly wide confidence intervals O were not possible to estimate in the SD_m24 model.In all cases it was not possible to obtain a reliable estimate of the regression coefficients.


#### Selected models

Subsequently, we inspect the residuals of the models whose confidence intervals do not overlap 0.

```{r}
#| layout-ncol: 2
#| fig-cap: 
#|  - "Residual inspection of model Tick Load ~ Month + (Avg_VH + I(Avg_VH^2))"
#|  - "Residual inspection of model Tick Load ~ Month"
#|  - "Residual inspection of model Tick Load ~ Month + Rep"
SD_res <- lapply(list(SD_m24, SD_m21, SD_m23), simulateResiduals, plot= TRUE)


```

The goodness-of-fit tests show a good fit of the models except for SD_m21, which contains only the month as an explanatory variable.

#### Prediction plots

```{r}
#| layout-ncol: 2
#| fig-cap: 
#|   -"Prediction plot of model Tick Load ~ Month + (Avg_VH + I(Avg_VH^2))"
#|   -"Prediction plot of model Tick Load ~ Month + Rep"
SD_Pred1 <- ggeffect(SD_m24, terms = c("Avg_VH", "Month"), ci_level = 0.85)

SD_Pred2 <- ggeffect(SD_m23, terms = c("Month","Rep"), ci_level = 0.85)

SD_pred1_plot <- ggplot()+
  geom_point(data = SIHI_D, aes(x= Avg_VH, y= n))+
  geom_ribbon(data= SD_Pred1, aes(x= x, y=predicted,
                                  ymin=conf.low, ymax=conf.high,
                                  fill=group), alpha= 0.6)+
  geom_line(data= SD_Pred1, aes(x= x, y= predicted, col= group),
            linewidth= 1)+
  labs(x= "Average vegetation height", y= "Expected tick load",
       fill= "Month",
       col= "Month")+
    guides(col= guide_legend(position = "inside"),
         fill= guide_legend(position = "inside"))+
    scale_color_viridis_d()+
    scale_fill_viridis_d()+
    theme_bw(base_size = 12)+
    theme(legend.position.inside = c(0.7, 0.8),
          legend.background = element_blank())


SD_pred2_plot <-ggplot()+
  geom_jitter(data=SIHI_D, aes(x= Month, y=n, col= Rep),
              alpha= 0.2,
              position = position_dodge(width = 0.5))+
  geom_pointrange(data = SD_Pred2, aes(x=x, y=predicted, 
                                   ymin= conf.low, ymax= conf.high,
                                   col=group), 
                  position = position_dodge(width = 0.5),
                  size=0.5, linewidth=1.2)+
  labs(y= "Expected tick load", 
       x= "Month", col= "Reproductive status")+
  guides( col=guide_legend(position = "inside"),
         fill= guide_legend(position = "inside"))+
  scale_color_manual(values = c("deepskyblue2", "dodgerblue4" ))+
  scale_fill_manual(values = c("deepskyblue2", "dodgerblue4" ))+
  theme_bw(base_size = 12)+
  theme(legend.position.inside = c(0.8, 0.8),
        legend.background = element_blank())


SD_pred1_plot 
SD_pred2_plot 
```


# Final Plots

```{r}
#| fig-height: 8.5
#| fig-width: 9
(predplots <- (OD_pred1_plot+OD_pred2_plot+ 
  PD_pred1_plot+PD_pred2_plot)+
  plot_layout(ncol = 2, axes = "collect")+
   plot_annotation(tag_levels = 'A'))


ggsave(plot = predplots, filename = "Figs/Load_predplots.png",
       width=9, height=9)

ggsave(plot = predplots, filename = "Figs/Load_predplots.svg",
       width=9, height=9)
  
```
```
