# Supplementary material for: Different drivers, same tick: Effect of host traits, habitat, and climate on the infestation of three rodent species by larval Dermacentor ticks
Source: Int J Parasitol Parasites Wildl. 2025 Mar 7;26:101054. doi: 10.1016/j.ijppaw.2025.101054 (PMC11929885; doi:10.1016/j.ijppaw.2025.101054)
Supplement: Multimedia component 3 [file mmc3.docx]

**Appendix A. Supplementary material**

Supplementary File 1. Statistical modeling process in R for the analysis of the prevalence of *Dermacentor* tick larvae in three rodent species.

Supplementary File 2. Statistical modeling process in R for the analysis of the load of *Dermacentor* tick larvae in three rodent species.

Table S 1 Final model selection for evaluating variables influencing the prevalence of Dermacentor larvae in Onychomys leucogaster.

| **Formula** | **K** | **AICc** | **Δ AICc** | **ModelLik** | **AICcWt** | **LL** |
| --- | --- | --- | --- | --- | --- | --- |
| Tick Prevalence ~ Average litter cover | 2 | 59.59 | 0.00 | 1.00 | 0.20 | -27.74 |
| Tick Prevalence ~ Body weight * Average litter cover | 4 | 59.85 | 0.26 | 0.88 | 0.18 | -25.76 |
| Tick Prevalence ~ Reproductive status + Average litter cover | 3 | 60.30 | 0.72 | 0.70 | 0.14 | -27.05 |
| Tick Prevalence ~ Sex + Average litter cover | 3 | 60.40 | 0.81 | 0.67 | 0.14 | -27.10 |
| Tick Prevalence ~ Sex * Average litter cover | 4 | 60.89 | 1.31 | 0.52 | 0.11 | -26.28 |
| Tick Prevalence ~ Body weight + Average litter cover | 3 | 61.45 | 1.86 | 0.39 | 0.08 | -27.62 |
| Tick Prevalence ~ Reproductive status * Average litter cover | 4 | 62.32 | 2.73 | 0.25 | 0.05 | -26.99 |
| Tick Prevalence ~ Average litter cover + Season | 4 | 63.19 | 3.60 | 0.17 | 0.03 | -27.43 |
| Tick Prevalence ~ Average litter cover * Season | 6 | 65.49 | 5.90 | 0.05 | 0.01 | -26.39 |
| Tick Prevalence ~ Rain | 2 | 66.06 | 6.47 | 0.04 | 0.01 | -30.98 |
| Tick Prevalence ~ 1 | 1 | 66.58 | 6.99 | 0.03 | 0.01 | -32.27 |
| Tick Prevalence ~ Reproductive status + Sex | 3 | 66.97 | 7.38 | 0.03 | 0.01 | -30.38 |
| Tick Prevalence ~ Reproductive status | 2 | 67.21 | 7.62 | 0.02 | 0.00 | -31.56 |
| Tick Prevalence ~ Sex | 2 | 67.34 | 7.75 | 0.02 | 0.00 | -31.62 |
| Tick Prevalence ~ Rain + Season | 4 | 67.67 | 8.09 | 0.02 | 0.00 | -29.67 |
| Tick Prevalence ~ Season | 3 | 67.83 | 8.24 | 0.02 | 0.00 | -30.81 |
| Tick Prevalence ~ Reproductive status + Season | 4 | 68.12 | 8.53 | 0.01 | 0.00 | -29.89 |
| Tick Prevalence ~ Reproductive status * Sex | 4 | 68.39 | 8.81 | 0.01 | 0.00 | -30.03 |
| Tick Prevalence ~ Body weight | 2 | 68.57 | 8.98 | 0.01 | 0.00 | -32.24 |
| Tick Prevalence ~ Sex + Season | 4 | 69.17 | 9.58 | 0.01 | 0.00 | -30.42 |
| Tick Prevalence ~ Sex + Body weight | 3 | 69.22 | 9.63 | 0.01 | 0.00 | -31.51 |
| Tick Prevalence ~ Reproductive status + Body weight | 3 | 69.28 | 9.69 | 0.01 | 0.00 | -31.54 |
| Tick Prevalence ~ Reproductive status * Body weight | 4 | 69.44 | 9.85 | 0.01 | 0.00 | -30.55 |
| Tick Prevalence ~ Rain * Season | 5 | 69.85 | 10.26 | 0.01 | 0.00 | -29.67 |
| Tick Prevalence ~ Body weight + Season | 4 | 69.89 | 10.30 | 0.01 | 0.00 | -30.78 |
| Tick Prevalence ~ Month | 7 | 69.96 | 10.37 | 0.01 | 0.00 | -27.49 |
| Tick Prevalence ~ Reproductive status * Season | 6 | 70.13 | 10.54 | 0.01 | 0.00 | -28.71 |
| Tick Prevalence ~ Body weight * Season | 6 | 70.76 | 11.17 | 0.00 | 0.00 | -29.02 |
| Tick Prevalence ~ Sex * Season | 5 | 70.83 | 11.25 | 0.00 | 0.00 | -30.16 |
| Tick Prevalence ~ Sex * Body weight | 4 | 70.91 | 11.32 | 0.00 | 0.00 | -31.29 |
| Tick Prevalence ~ Month * Reproductive status | 12 | 78.68 | 19.09 | 0.00 | 0.00 | -25.94 |
| Tick Prevalence ~ Month * Sex | 12 | 78.75 | 19.16 | 0.00 | 0.00 | -25.97 |

Table S 2 Final model selection for evaluating variables influencing the prevalence of Dermacentor larvae in *Peromyscus leucopus*.

| **Formula** | **K** | **AICc** | **Δ AICc** | **ModelLik** | **AICcWt** | **LL** |
| --- | --- | --- | --- | --- | --- | --- |
| Tick Prevalence ~ Month | 7 | 357.24 | 0.00 | 1.00 | 0.58 | -171.45 |
| Tick Prevalence ~ Month * Reproductive status | 14 | 359.72 | 2.48 | 0.29 | 0.17 | -165.19 |
| Tick Prevalence ~ Month * Sex | 14 | 360.61 | 3.37 | 0.19 | 0.11 | -165.64 |
| Tick Prevalence ~ Month * Average litter cover | 14 | 361.33 | 4.09 | 0.13 | 0.08 | -166.00 |
| Tick Prevalence ~ Month * Body weight | 14 | 362.14 | 4.90 | 0.09 | 0.05 | -166.41 |
| Tick Prevalence ~ Month * Square (Body weight) | 21 | 363.90 | 6.66 | 0.04 | 0.02 | -159.45 |
| Tick Prevalence ~ Month * Square (Temperature) | 21 | 373.24 | 16.00 | 0.00 | 0.00 | -164.12 |
| Tick Prevalence ~ Average litter cover + Square (Temperature) | 4 | 380.87 | 23.63 | 0.00 | 0.00 | -186.37 |
| Tick Prevalence ~ Square (Average vegetation height) + Square (Temperature) | 5 | 381.71 | 24.47 | 0.00 | 0.00 | -185.76 |
| Tick Prevalence ~ Average vegetation height * Square (Temperature) | 6 | 381.79 | 24.55 | 0.00 | 0.00 | -184.77 |
| Tick Prevalence ~ Average litter cover * Square (Temperature) | 5 | 382.33 | 25.09 | 0.00 | 0.00 | -186.07 |
| Tick Prevalence ~ Average vegetation height + Square (Temperature) | 4 | 383.50 | 26.26 | 0.00 | 0.00 | -187.69 |
| Tick Prevalence ~ Square (Body weight) + Square (Temperature) | 5 | 385.01 | 27.77 | 0.00 | 0.00 | -187.41 |
| Tick Prevalence ~ Reproductive status * Square (Temperature) | 6 | 385.15 | 27.91 | 0.00 | 0.00 | -186.45 |
| Tick Prevalence ~ Reproductive status + Square (Temperature) | 4 | 386.91 | 29.67 | 0.00 | 0.00 | -189.39 |
| Tick Prevalence ~ Square (Temperature) | 3 | 388.36 | 31.12 | 0.00 | 0.00 | -191.15 |
| Tick Prevalence ~ Sex + Square (Temperature) | 4 | 388.65 | 31.41 | 0.00 | 0.00 | -190.26 |
| Tick Prevalence ~ Sex * Square (Temperature) | 6 | 389.11 | 31.87 | 0.00 | 0.00 | -188.42 |
| Tick Prevalence ~ Body weight + Square (Temperature) | 4 | 390.11 | 32.87 | 0.00 | 0.00 | -190.99 |
| Tick Prevalence ~ Body weight * Square (Temperature) | 6 | 393.05 | 35.81 | 0.00 | 0.00 | -190.40 |
| Tick Prevalence ~ Reproductive status + Square (Average vegetation height^2) | 4 | 400.64 | 43.40 | 0.00 | 0.00 | -196.26 |
| Tick Prevalence ~ Reproductive status + Average vegetation height | 3 | 402.01 | 44.77 | 0.00 | 0.00 | -197.97 |
| Tick Prevalence ~ Reproductive status * Average litter cover | 4 | 402.36 | 45.12 | 0.00 | 0.00 | -197.12 |
| Tick Prevalence ~ Reproductive status + Average litter cover | 3 | 402.84 | 45.60 | 0.00 | 0.00 | -198.39 |
| Tick Prevalence ~ Reproductive status * Average vegetation height | 4 | 403.65 | 46.41 | 0.00 | 0.00 | -197.76 |
| Tick Prevalence ~ Reproductive status * Square (Average vegetation height) | 6 | 404.02 | 46.78 | 0.00 | 0.00 | -195.88 |
| Tick Prevalence ~ Square (Body weight) + Average vegetation height | 4 | 404.52 | 47.28 | 0.00 | 0.00 | -198.20 |
| Tick Prevalence ~ Body weight * Average litter cover | 4 | 404.59 | 47.35 | 0.00 | 0.00 | -198.23 |
| Tick Prevalence ~ Sex + Square (Average vegetation height) | 4 | 404.61 | 47.37 | 0.00 | 0.00 | -198.25 |
| Tick Prevalence ~ Reproductive status * Body weight | 4 | 404.79 | 47.55 | 0.00 | 0.00 | -198.33 |
| Tick Prevalence ~ Square (Body weight) * Average litter cover | 5 | 404.81 | 47.57 | 0.00 | 0.00 | -197.31 |
| Tick Prevalence ~ Square (Body weight) + Square (Average vegetation height) | 5 | 404.87 | 47.63 | 0.00 | 0.00 | -197.35 |
| Tick Prevalence ~ Square (Average vegetation height) | 3 | 405.02 | 47.78 | 0.00 | 0.00 | -199.47 |
| Tick Prevalence ~ Average vegetation height | 2 | 405.36 | 48.12 | 0.00 | 0.00 | -200.66 |
| Tick Prevalence ~ Sex + Average vegetation height | 3 | 405.42 | 48.18 | 0.00 | 0.00 | -199.68 |
| Tick Prevalence ~ Sex * Square (Body weight) | 6 | 405.81 | 48.57 | 0.00 | 0.00 | -196.78 |
| Tick Prevalence ~ Sex + Average litter cover | 3 | 405.81 | 48.57 | 0.00 | 0.00 | -199.87 |
| Tick Prevalence ~ Average litter cover | 2 | 405.84 | 48.60 | 0.00 | 0.00 | -200.90 |
| Tick Prevalence ~ Square (Body weight) + Average litter cover | 4 | 405.84 | 48.60 | 0.00 | 0.00 | -198.86 |
| Tick Prevalence ~ Sex * Body weight | 4 | 405.94 | 48.70 | 0.00 | 0.00 | -198.91 |
| Tick Prevalence ~ Reproductive status + Body weight | 3 | 406.30 | 49.06 | 0.00 | 0.00 | -200.12 |
| Tick Prevalence ~ Reproductive status + Square (Body weight) | 4 | 406.36 | 49.12 | 0.00 | 0.00 | -199.12 |
| Tick Prevalence ~ Body weight + Square (Average vegetation height) | 4 | 406.58 | 49.34 | 0.00 | 0.00 | -199.23 |
| Tick Prevalence ~ Average vegetation height * Average litter cover | 4 | 406.61 | 49.37 | 0.00 | 0.00 | -199.24 |
| Tick Prevalence ~ Body weight + Average vegetation height | 3 | 406.69 | 49.45 | 0.00 | 0.00 | -200.31 |
| Tick Prevalence ~ Average vegetation height + Average litter cover | 3 | 406.71 | 49.47 | 0.00 | 0.00 | -200.32 |
| Tick Prevalence ~ Square (Average vegetation height) + Average litter cover | 4 | 407.03 | 49.79 | 0.00 | 0.00 | -199.45 |
| Tick Prevalence ~ Body weight + Average litter cover | 3 | 407.03 | 49.79 | 0.00 | 0.00 | -200.48 |
| Tick Prevalence ~ Sex * Average vegetation height | 4 | 407.06 | 49.82 | 0.00 | 0.00 | -199.47 |
| Tick Prevalence ~ Square (Body weight) * Average vegetation height | 6 | 407.45 | 50.21 | 0.00 | 0.00 | -197.59 |
| Tick Prevalence ~ Sex * Average litter cover | 4 | 407.68 | 50.44 | 0.00 | 0.00 | -199.78 |
| Tick Prevalence ~ Square (Body weight) | 3 | 407.75 | 50.51 | 0.00 | 0.00 | -200.84 |
| Tick Prevalence ~ Sex * Square (Average vegetation height) | 6 | 408.01 | 50.77 | 0.00 | 0.00 | -197.88 |
| Tick Prevalence ~ Sex + Square (Body weight) | 4 | 408.16 | 50.92 | 0.00 | 0.00 | -200.02 |
| Tick Prevalence ~ Body weight * Average vegetation height | 4 | 408.42 | 51.18 | 0.00 | 0.00 | -200.15 |
| Tick Prevalence ~ Reproductive status * Square (Body weight) | 6 | 408.44 | 51.20 | 0.00 | 0.00 | -198.09 |
| Tick Prevalence ~ Reproductive status * Sex | 4 | 408.50 | 51.26 | 0.00 | 0.00 | -200.19 |
| Tick Prevalence ~ Reproductive status | 2 | 408.72 | 51.48 | 0.00 | 0.00 | -202.34 |
| Tick Prevalence ~ Sex | 2 | 409.12 | 51.88 | 0.00 | 0.00 | -202.54 |
| Tick Prevalence ~ Body weight | 2 | 409.19 | 51.95 | 0.00 | 0.00 | -202.58 |
| Tick Prevalence ~ 1 | 1 | 409.24 | 52.00 | 0.00 | 0.00 | -203.62 |
| Tick Prevalence ~ Sex + Body weight | 3 | 409.37 | 52.13 | 0.00 | 0.00 | -201.65 |
| Tick Prevalence ~ Reproductive status + Sex | 3 | 409.62 | 52.38 | 0.00 | 0.00 | -201.77 |

Table S 3 Final model selection for evaluating variables influencing the prevalence of Dermacentor larvae in *Sigmodon hispidus*.

| **Formula** | **K** | **AICc** | **Δ AICc** | **ModelLik** | **AICcWt** | **LL** |
| --- | --- | --- | --- | --- | --- | --- |
| Tick Prevalence ~ Reproductive status + Square (Average vegetation height) | 4 | 160.51 | 0.00 | 1.00 | 0.40 | -76.21 |
| Tick Prevalence ~ Square (Average vegetation height) * Season | 7 | 162.08 | 1.57 | 0.46 | 0.18 | -73.93 |
| Tick Prevalence ~ Square (Average vegetation height) + Season | 5 | 162.23 | 1.72 | 0.42 | 0.17 | -76.05 |
| Tick Prevalence ~ Square (Average vegetation height) | 3 | 162.96 | 2.45 | 0.29 | 0.12 | -78.45 |
| Tick Prevalence ~ Month + Reproductive status | 8 | 163.24 | 2.73 | 0.26 | 0.10 | -73.47 |
| Tick Prevalence ~ Month | 7 | 166.52 | 6.01 | 0.05 | 0.02 | -76.15 |
| Tick Prevalence ~ Month * Reproductive status | 13 | 171.87 | 11.36 | 0.00 | 0.00 | -72.56 |
| Tick Prevalence ~ Reproductive status + Season | 4 | 172.83 | 12.32 | 0.00 | 0.00 | -82.38 |
| Tick Prevalence ~ Reproductive status | 2 | 174.81 | 14.30 | 0.00 | 0.00 | -85.39 |
| Tick Prevalence ~ Season | 3 | 174.97 | 14.46 | 0.00 | 0.00 | -84.46 |
| Tick Prevalence ~ Reproductive status * Season | 6 | 176.20 | 15.69 | 0.00 | 0.00 | -82.02 |
| Tick Prevalence ~ 1 | 1 | 176.77 | 16.26 | 0.00 | 0.00 | -87.38 |

Table S 4 Selected model estimates with 85% confidence intervals for analyzing the prevalence of *Dermacentor* tick larvae in two rodent species. Variables with CI that did not overlap 0 were considered in the interpretation.

| **Model** | **Parameter** | **Coefficient** | **Lower CI** | **Upper CI** | **p** | **Effects** |
| --- | --- | --- | --- | --- | --- | --- |
| ***Onychomys leucogaster*** | | | | | | |
| Tick Prevalence ~ Weight * Average litter cover | Intercept | -12.96 | -18.68 | -7.23 | 0.00 | fixed |
|  | Weight | 0.21 | 0.07 | 0.34 | 0.03 | fixed |
|  | Average litter cover | 0.33 | 0.17 | 0.49 | 0.00 | fixed |
|  | Weight* Average litter cover | -0.01 | -0.01 | 0.00 | 0.02 | fixed |
| Tick Prevalence ~ Average litter cover | Intercept | -4.84 | -6.28 | -3.40 | 0.00 | fixed |
|  | Average litter cover | 0.08 | 0.04 | 0.12 | 0.00 | fixed |
| ***Peromyscus leucopus*** | | | | | | |
| Tick Prevalence ~ Month | Intercept | 0.17 | -0.32 | 0.66 | 0.61 | fixed |
|  | Month3 | -0.38 | -1.11 | 0.35 | 0.45 | fixed |
|  | Month5 | -1.27 | -2.04 | -0.51 | 0.02 | fixed |
|  | Month9 | -3.89 | -5.42 | -2.35 | 0.00 | fixed |
|  | Month10 | -2.25 | -2.96 | -1.55 | 0.00 | fixed |
|  | Month11 | -0.24 | -0.81 | 0.34 | 0.55 | fixed |
|  | Month12 | -0.87 | -1.69 | -0.04 | 0.13 | fixed |

Table S 5 Final model selection for evaluating variables influencing the load of Dermacentor larvae in Onychomys leucogaster.

| **Formula** | **K** | **AICc** | **Δ AICc** | **ModelLik** | **AICcWt** | **LL** |
| --- | --- | --- | --- | --- | --- | --- |
| Tick Load ~ Body weight * Average litter cover | 5 | 82.7 | 0 | 1 | 0.28 | -36.1 |
| Tick Load ~ Sex + Average litter cover | 4 | 82.98 | 0.28 | 0.87 | 0.24 | -37.3 |
| Tick Load ~ Average litter cover + Rain | 4 | 83.76 | 1.06 | 0.59 | 0.17 | -37.7 |
| Tick Load ~ Average litter cover | 3 | 84.46 | 1.76 | 0.42 | 0.12 | -39.1 |
| Tick Load ~ Sex * Average litter cover | 5 | 85.15 | 2.45 | 0.29 | 0.08 | -37.3 |
| Tick Load ~ Average litter cover + Temperature | 4 | 86.57 | 3.87 | 0.14 | 0.04 | -39.1 |
| Tick Load ~ Body weight + Average litter cover | 4 | 86.57 | 3.87 | 0.14 | 0.04 | -39.1 |
| Tick Load ~ Average litter cover * Temperature | 5 | 88.65 | 5.95 | 0.05 | 0.01 | -39.1 |
| Tick Load ~ Sex | 3 | 92.26 | 9.56 | 0.01 | 0 | -43 |
| Tick Load ~ Sex + Rain | 4 | 92.4 | 9.69 | 0.01 | 0 | -42 |
| Tick Load ~ Rain | 3 | 92.6 | 9.9 | 0.01 | 0 | -43.2 |
| Tick Load ~ 1 | 2 | 93.05 | 10.35 | 0.01 | 0 | -44.5 |
| Tick Load ~ Rain + Temperature | 4 | 93.27 | 10.57 | 0.01 | 0 | -42.5 |
| Tick Load ~ Body weight + Rain | 4 | 93.58 | 10.88 | 0 | 0 | -42.6 |
| Tick Load ~ Sex + Body weight | 4 | 93.76 | 11.06 | 0 | 0 | -42.7 |
| Tick Load ~ Sex + Temperature | 4 | 93.95 | 11.25 | 0 | 0 | -42.8 |
| Tick Load ~ Body weight | 3 | 94.03 | 11.33 | 0 | 0 | -43.9 |
| Tick Load ~ Temperature | 3 | 94.13 | 11.43 | 0 | 0 | -44 |
| Tick Load ~ Sex * Rain | 5 | 94.57 | 11.87 | 0 | 0 | -42 |
| Tick Load ~ Rain * Temperature | 5 | 95.44 | 12.74 | 0 | 0 | -42.5 |
| Tick Load ~ Body weight + Temp | 4 | 95.48 | 12.78 | 0 | 0 | -43.6 |
| Tick Load ~ Month | 8 | 95.62 | 12.92 | 0 | 0 | -39.2 |
| Tick Load ~ Body weight * Rain | 5 | 95.75 | 13.05 | 0 | 0 | -42.6 |
| Tick Load ~ Sex * Temperature | 5 | 95.92 | 13.22 | 0 | 0 | -42.7 |
| Tick Load ~ Body weight * Temp | 5 | 97.41 | 14.71 | 0 | 0 | -43.5 |
| Tick Load ~ Reproductive status * Body weight | 5 | 98.09 | 15.39 | 0 | 0 | -43.8 |
| Tick Load ~ Month * Sex | 13 | 102.2 | 19.45 | 0 | 0 | -36.4 |
| Tick Load ~ Month * Temperature | 13 | 103.7 | 20.99 | 0 | 0 | -37.2 |

Table S 6 Final model selection for evaluating variables influencing the load of Dermacentor larvae in *Peromyscus leucopus*.

| **Formula** | **K** | **AICc** | **Δ AICc** | **ModelLik** | **AICcWt** | **LL** |
| --- | --- | --- | --- | --- | --- | --- |
| Tick Load ~ Month * Sex | 15 | 839.18 | 0 | 1 | 0.26 | -403.83 |
| Tick Load ~ Month + Reproductive status | 9 | 839.49 | 0.31 | 0.86 | 0.22 | -410.46 |
| Tick Load ~ Month * Average litter cover | 15 | 839.85 | 0.67 | 0.72 | 0.19 | -404.16 |
| Tick Load ~ Month | 8 | 841.64 | 2.46 | 0.29 | 0.08 | -412.6 |
| Tick Load ~ Month + Sex | 9 | 841.79 | 2.61 | 0.27 | 0.07 | -411.61 |
| Tick Load ~ Month + Average litter cover | 9 | 842.23 | 3.05 | 0.22 | 0.06 | -411.83 |
| Tick Load ~ Month * Reproductive status | 15 | 843.05 | 3.87 | 0.14 | 0.04 | -405.76 |
| Tick Load ~ Month + Temperature | 9 | 843.39 | 4.21 | 0.12 | 0.03 | -412.41 |
| Tick Load ~ Month * Temperature | 15 | 843.47 | 4.29 | 0.12 | 0.03 | -405.97 |
| Tick Load ~ Month + Square (Temperature) | 10 | 844.35 | 5.17 | 0.08 | 0.02 | -411.83 |
| Tick Load ~ Month * Square (Temperature) | 22 | 857.43 | 18.25 | 0 | 0 | -405.07 |
| Tick Load ~ Average litter cover+ Square (Temperature) | 5 | 869.97 | 30.78 | 0 | 0 | -429.89 |
| Tick Load ~ Reproductive status * Square (Temperature) | 7 | 873.22 | 34.04 | 0 | 0 | -429.44 |
| Tick Load ~ Average litter cover * Square (Temperature) | 7 | 873.55 | 34.37 | 0 | 0 | -429.6 |
| Tick Load ~ Average litter cover + Temperature | 4 | 873.67 | 34.48 | 0 | 0 | -432.77 |
| Tick Load ~ Sex * Square (Temperature) | 7 | 875 | 35.82 | 0 | 0 | -430.33 |
| Tick Load ~ Average litter cover * Temperature | 5 | 875.72 | 36.54 | 0 | 0 | -432.77 |
| Tick Load ~ Reproductive status * Temp | 5 | 877.03 | 37.85 | 0 | 0 | -433.42 |
| Tick Load ~ Sex * Temperature | 5 | 877.51 | 38.33 | 0 | 0 | -433.67 |
| Tick Load ~ Sex + Temperature | 4 | 877.85 | 38.67 | 0 | 0 | -434.86 |
| Tick Load ~ Sex + Square (Temperature) | 5 | 877.96 | 38.78 | 0 | 0 | -433.89 |
| Tick Load ~ Reproductive status + Square (Temperature) | 5 | 878.72 | 39.54 | 0 | 0 | -434.27 |
| Tick Load ~ Reproductive status + Temperature | 4 | 878.79 | 39.61 | 0 | 0 | -435.33 |
| Tick Load ~ Square (Temperature) | 4 | 880.37 | 41.18 | 0 | 0 | -436.12 |
| Tick Load ~ Reproductive status * Average litter cover | 5 | 889.38 | 50.19 | 0 | 0 | -439.6 |
| Tick Load ~ Reproductive status + Average litter cover | 4 | 890.7 | 51.52 | 0 | 0 | -441.29 |
| Tick Load ~ Sex + Average litter cover | 4 | 891.25 | 52.07 | 0 | 0 | -441.56 |
| Tick Load ~ Reproductive status * Sex | 5 | 892.71 | 53.53 | 0 | 0 | -441.26 |
| Tick Load ~ Sex * Average litter cover | 5 | 892.77 | 53.59 | 0 | 0 | -441.29 |
| Tick Load ~ Sex | 3 | 894.13 | 54.94 | 0 | 0 | -444.03 |
| Tick Load ~ Reproductive status + Sex | 4 | 894.26 | 55.07 | 0 | 0 | -443.07 |
| Tick Load ~ Reproductive status | 3 | 896 | 56.82 | 0 | 0 | -444.96 |
| Tick Load ~ Average litter cover | 3 | 896.58 | 57.39 | 0 | 0 | -445.25 |
| Tick Load ~ 1 | 2 | 900.39 | 61.21 | 0 | 0 | -448.18 |

Table S 7 Final model selection for evaluating variables influencing the load of Dermacentor larvae in *Sigmodon hispidus*.

| **Formula** | **K** | **AICc** | **Δ AICc** | **ModelLik** | **AICcWt** | **LL** |
| --- | --- | --- | --- | --- | --- | --- |
| Tick Load ~ Month + Square (Average vegetation height) | 10 | 235.45 | 0 | 1 | 0.35 | -107.5 |
| Tick Load ~ Month | 8 | 236.16 | 0.71 | 0.7 | 0.25 | -109.93 |
| Tick Load ~ Month + Reproductive status | 9 | 236.3 | 0.85 | 0.65 | 0.23 | -108.97 |
| Tick Load ~ Month + Sex | 9 | 238.18 | 2.73 | 0.25 | 0.09 | -109.91 |
| Tick Load ~ Reproductive status * Square (Average vegetation height) | 7 | 239.57 | 4.12 | 0.13 | 0.04 | -112.67 |
| Tick Load ~ Square (Average vegetation height) + Season | 6 | 242.93 | 7.48 | 0.02 | 0.01 | -115.38 |
| Tick Load ~ Reproductive status + Square (Average vegetation height) | 5 | 243.13 | 7.68 | 0.02 | 0.01 | -116.5 |
| Tick Load ~ Square (Average vegetation height) | 4 | 243.22 | 7.78 | 0.02 | 0.01 | -117.57 |
| Tick Load ~ Month * Reproductive status | 14 | 243.88 | 8.44 | 0.01 | 0.01 | -107.51 |
| Tick Load ~ Sex + Square (Average vegetation height) | 5 | 245.15 | 9.71 | 0.01 | 0 | -117.52 |
| Tick Load ~ Sex * Square (Average vegetation height) | 7 | 246.54 | 11.1 | 0 | 0 | -116.16 |
| Tick Load ~ Season | 4 | 249.06 | 13.61 | 0 | 0 | -120.49 |
| Tick Load ~ Reproductive status + Season | 5 | 249.65 | 14.21 | 0 | 0 | -119.77 |
| Tick Load ~ Sex + Season | 5 | 250.98 | 15.54 | 0 | 0 | -120.43 |
| Tick Load ~ Reproductive status * Season | 7 | 252.14 | 16.69 | 0 | 0 | -118.96 |
| Tick Load ~ Sex * Season | 7 | 252.44 | 17 | 0 | 0 | -119.11 |
| Tick Load ~ Reproductive status + Sex | 4 | 254.7 | 19.25 | 0 | 0 | -123.31 |
| Tick Load ~ Reproductive status | 3 | 254.93 | 19.48 | 0 | 0 | -124.44 |
| Tick Load ~ 1 | 2 | 255.51 | 20.07 | 0 | 0 | -125.74 |
| Tick Load ~ Sex | 3 | 255.8 | 20.35 | 0 | 0 | -124.87 |
| Tick Load ~ Reproductive status * Sex | 5 | 255.97 | 20.53 | 0 | 0 | -122.93 |
| Tick Load ~ Sex + Body weight | 4 | 256.64 | 21.19 | 0 | 0 | -124.28 |
| Tick Load ~ Reproductive status + Body weight | 4 | 256.67 | 21.22 | 0 | 0 | -124.29 |
| Tick Load ~ Reproductive status * Body weight | 5 | 258.21 | 22.77 | 0 | 0 | -124.05 |
| Tick Load ~ Sex * Body weight | 5 | 258.67 | 23.22 | 0 | 0 | -124.27 |

Table S 8 Selected model estimates with 85% confidence intervals for analyzing the load of Dermacentor tick larvae in two rodent species. Variables with CI that did not overlap 0 were considered in the interpretation

| **Model** | **Parameter** | **Coefficient** | **Lower CI** | **Upper CI** | **p** | **Component** | **Effects** |
| --- | --- | --- | --- | --- | --- | --- | --- |
| ***Onychomys leucogaster*** | | | | | | | |
| Tick Load ~ Body weight * Average litter cover | Intercept | -15.84 | -24.31 | -7.36 | 0.01 | conditional | fixed |
|  | Body weight | 0.27 | 0.08 | 0.47 | 0.04 | conditional | fixed |
|  | Average litter cover | 0.47 | 0.23 | 0.72 | 0.01 | conditional | fixed |
|  | Body weight: Average litter cover | -0.01 | -0.02 | 0.00 | 0.02 | conditional | fixed |
|  | Intercept | 0.23 | 0.10 | 0.53 | NA | dispersion | fixed |
| Tick Load ~ Sex + Average litter cover | Intercept | -4.89 | -6.56 | -3.23 | 0.00 | conditional | fixed |
|  | Sex Male | -2.00 | -3.60 | -0.39 | 0.07 | conditional | fixed |
|  | Average litter cover | 0.11 | 0.06 | 0.16 | 0.00 | conditional | fixed |
|  | Intercept | 0.18 | 0.08 | 0.41 | NA | dispersion | fixed |
| Tick Load ~ Average litter cover | Intercept | -5.33 | -7.05 | -3.60 | 0.00 | conditional | fixed |
|  | Average litter cover | 0.11 | 0.06 | 0.16 | 0.00 | conditional | fixed |
|  | Intercept | 0.14 | 0.06 | 0.30 | NA | dispersion | fixed |
| ***Peromyscus leucopus*** | | | | | | | |
| Tick Load ~ Month * Sex | Intercept | 0.80 | 0.04 | 1.57 | 0.13 | conditional | fixed |
|  | Month3 | -0.80 | -2.15 | 0.55 | 0.39 | conditional | fixed |
|  | Month5 | -1.33 | -2.42 | -0.25 | 0.08 | conditional | fixed |
|  | Month9 | -20.38 | -6632.92 | 6592.17 | 1.00 | conditional | fixed |
|  | Month10 | -1.61 | -2.56 | -0.67 | 0.01 | conditional | fixed |
|  | Month11 | -0.80 | -1.72 | 0.12 | 0.21 | conditional | fixed |
|  | Month12 | -1.31 | -2.57 | -0.05 | 0.13 | conditional | fixed |
|  | Sex Male | 0.49 | -0.47 | 1.44 | 0.46 | conditional | fixed |
|  | Month3: Sex Male | 0.16 | -1.42 | 1.74 | 0.89 | conditional | fixed |
|  | Month5:Sex Male | -0.59 | -2.07 | 0.90 | 0.57 | conditional | fixed |
|  | Month9:Sex Male | 15.79 | -6596.76 | 6628.34 | 1.00 | conditional | fixed |
|  | Month10:Sex Male | -2.10 | -3.47 | -0.72 | 0.03 | conditional | fixed |
|  | Month11:Sex Male | 0.81 | -0.33 | 1.95 | 0.31 | conditional | fixed |
|  | Month12:Sex Male | 0.88 | -0.73 | 2.50 | 0.43 | conditional | fixed |
|  | Intercept | 0.31 | 0.25 | 0.39 | NA | dispersion | fixed |
| Tick Load ~ Month + Reproductive status | Intercept | 0.77 | 0.25 | 1.30 | 0.03 | conditional | fixed |
|  | Month3 | -0.63 | -1.35 | 0.09 | 0.21 | conditional | fixed |
|  | Month5 | -1.52 | -2.29 | -0.76 | 0.00 | conditional | fixed |
|  | Month9 | -5.03 | -6.61 | -3.45 | 0.00 | conditional | fixed |
|  | Month10 | -2.53 | -3.18 | -1.88 | 0.00 | conditional | fixed |
|  | Month11 | -0.31 | -0.88 | 0.27 | 0.44 | conditional | fixed |
|  | Month12 | -0.73 | -1.53 | 0.06 | 0.19 | conditional | fixed |
|  | Reproductive statusY | 0.61 | 0.20 | 1.02 | 0.03 | conditional | fixed |
|  | Intercept | 0.29 | 0.23 | 0.36 | NA | dispersion | fixed |
